# Engineering Ultrastable Intrinsic Radicals: Graphene Quantum Dots With NIR‐II Emission for Dynamic Deep‐Tissue Bioimaging

**DOI:** 10.1002/advs.76970

**Published:** 2026-08-03

**Authors:** Qin Xu, Yijie Hou, Bingzhe Wang, Tesen Zhang, Yupeng Liu, Shuaiqi Li, Maohua Chen, Guichuan Xing, Dongbo Guo, Songnan Qu

**Affiliations:** ^1^ State Key Laboratory of Digital Medical Engineering Key Laboratory of Biomedical Engineering of Hainan Province School of Biomedical Engineering Hainan University Sanya China; ^2^ Joint Key Laboratory of the Ministry of Education Institute of Applied Physics and Materials Engineering University of Macau Taipa Macau SAR China; ^3^ Interdisciplinary Institute of Medical Engineering Fuzhou University Fuzhou China

**Keywords:** deep‐tissue bioimaging, graphene quantum dots, intrinsic radicals, NIR‐II, open‐shell

## Abstract

Stable radicals in carbon‐based materials have witnessed great consideration in the field of photonic and quantum information technologies, yet their development has been hindered by inherent instability arising from high reactivity. Here, we demonstrate the stabilization of intrinsic radicals within graphene quantum dots (GQDs) synthesized via two‐dimensional polymerization of perylene derivatives. Numerous radicals are stably localized at periodically arranged lattice defects in bilayer graphene‐like plates, where the induced structural distortions and intralayer oscillations minimize interlayer interactions, thus enabling exceptional radical stability across a broad temperature range (100–500 K). This configuration generates an intrinsic radical defect (IRD) state with an absorption band extending beyond 780 nm. Via experimental and theoretical investigations, we reveal that the radical‐related singly occupied molecular orbital (SOMO) level facilitates exciton dissociation and diffusion from *π*‐conjugated domains to IRD domains with an efficient charge transfer process. This process yields efficient second near‐infrared (NIR‐II) photoluminescence with a quantum yield of 1.8%. Leveraging this property, the GQDs facilitate high‐resolution NIR‐II fluorescence imaging of vessels, tumors, and lymph nodes in mice. This discovery boosts the applications of carbon‐based radicals in photonic technologies.

## Graphical abstract

Stable radicals are attractive for photonic and quantum information technologies but are often limited by instability. We stabilize intrinsic radicals in graphene quantum dots via 2D polymerization of perylene derivatives, where bilayer defect confinement suppresses quenching (100–500 K). The resulting IRD state absorbs >780 nm, enables SOMO‐mediated charge transfer, and yields NIR‐II emission (PLQY 1.8%) for high‐resolution in vivo imaging.

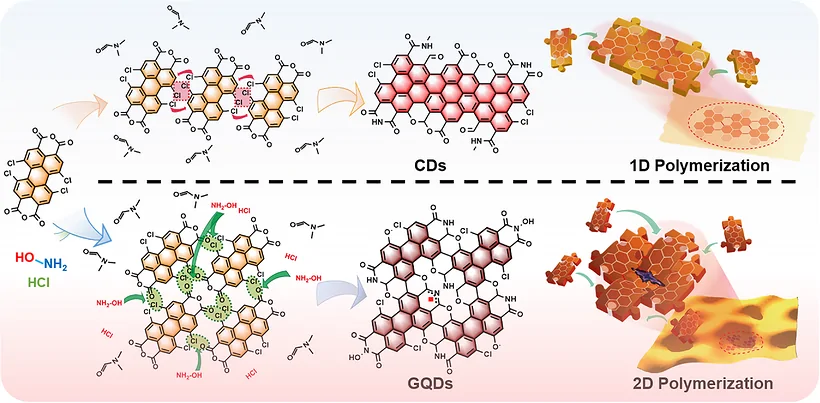

## 1 Introduction

Open‐shell radicals, characterized by their unpaired electrons, novel spin states, and metal‐free nature, represent a cutting‐edge research direction at the intersection of photonics and quantum information technology [1, 2, 3]. Due to the energy level of the singly occupied molecular orbital (SOMO) is lower than the lowest unoccupied molecular orbital (LUMO) of a comparable closed‐shell system, open‐shell radicals inherently exhibit narrow bandgaps, rendering them promising candidates for generating doublet emission in the near‐infrared (NIR) region [4, 5, 6]. Nevertheless, their significant chemical reactivity, coupled with limitations in scalability, long‐term stability, and production efficiency, has impeded applications in photonic operations and information storage [7, 8, 9]. To enhance the stability of radical materials, prevalent strategies typically include shielding the radicals through deliberately increased steric hindrance [7, 10, 11, 12], promoting delocalization of the unpaired electron across extended *π*‐conjugated backbones, or localizing radicals at topological defect sites [13, 14, 15, 16].

Recent advances in the directional synthesis of nanographene from molecular precursors have brought this area to a prominent research focus in the field of open‐shell materials [17, 18, 19]. However, currently reported radicals in nanographene were primarily confined to zigzag and armchair edges [20]. Nevertheless, the precise engineering of ordered radical arrays in graphene via chemical functionalization or scanning probe‐assisted dehydrogenation faces significant challenges, including reliance on expensive instrumentation and limited scalability [21]. Graphene quantum dots (GQDs), a distinct subclass of carbon dots (CDs), feature single‐ or multi‐layered graphene core structures [22, 23, 24, 25, 26, 27]. These GQDs demonstrate not only tunable fluorescence and biocompatibility but also enable cost‐effective, large‐scale preparation [28, 29, 30]. Our previous work revealed that post‐oxidation treatment of CDs introduces oxygen‐related defects into the carbon‐based core, resulting in stable NIR luminescence [31]. Trapping radicals at defects within sp^2^ domains presents a viable radical stabilization strategy via engineered *π*‐electron topologies [32, 33, 34]. Nevertheless, the synthesis of graphene nanomaterials incorporating high‐density radicals and exhibiting long‐term stability across a wide temperature range remains unreported.

Perylene derivatives, a type of large *π*‐conjugated molecules, have been demonstrated as precursors for synthesizing bandgap‐tunable GQDs [35, 36, 37]. However, stable NIR luminescence originating from a radical state hasn't been achieved in perylene‐derived GQDs. Here, we realize intrinsic radical state in GQDs with excellent stability across a wide temperature range, by trapping radicals at periodic N, O defects in a graphene‐like lattice. Using a solvothermal two‐dimensional polymerization of 1,6,7,12‐tetrachloroperylene tetracarboxylic acid dianhydride (PDI‐Cl) with hydroxylamine hydrochloride, we generate high‐content radicals in the resulting GQDs (Figure 1a and Figure S1). Periodic N, O doping induced structural distortions and oscillations in the bilayer graphene plates, which in turn stabilized the radicals localized at the defects by minimizing interlayer interactions. This structural modulation enhanced radical absorption in the 700–1000 nm range and promotes NIR emission. Experimental and theoretical analyses demonstrate that these intrinsic radicals significantly reduced the bandgap, facilitating exciton dissociation and diffusion from the *π*‐conjugation domains to the IRD domains. This process produces NIR fluorescence peaking at 855 nm with a tail extending to 1300 nm under 808 nm excitation. Following micelle encapsulation, the modified GQDs enabled high‐spatial‐resolution real‐time NIR‐II imaging of blood vessels, tumors, and lymph nodes in mice. The solvothermal two‐dimensional polymerization for fabricating ultrastable radicals might boost the applications of carbon‐based radical in photonics and spintronics.

**FIGURE 1:**
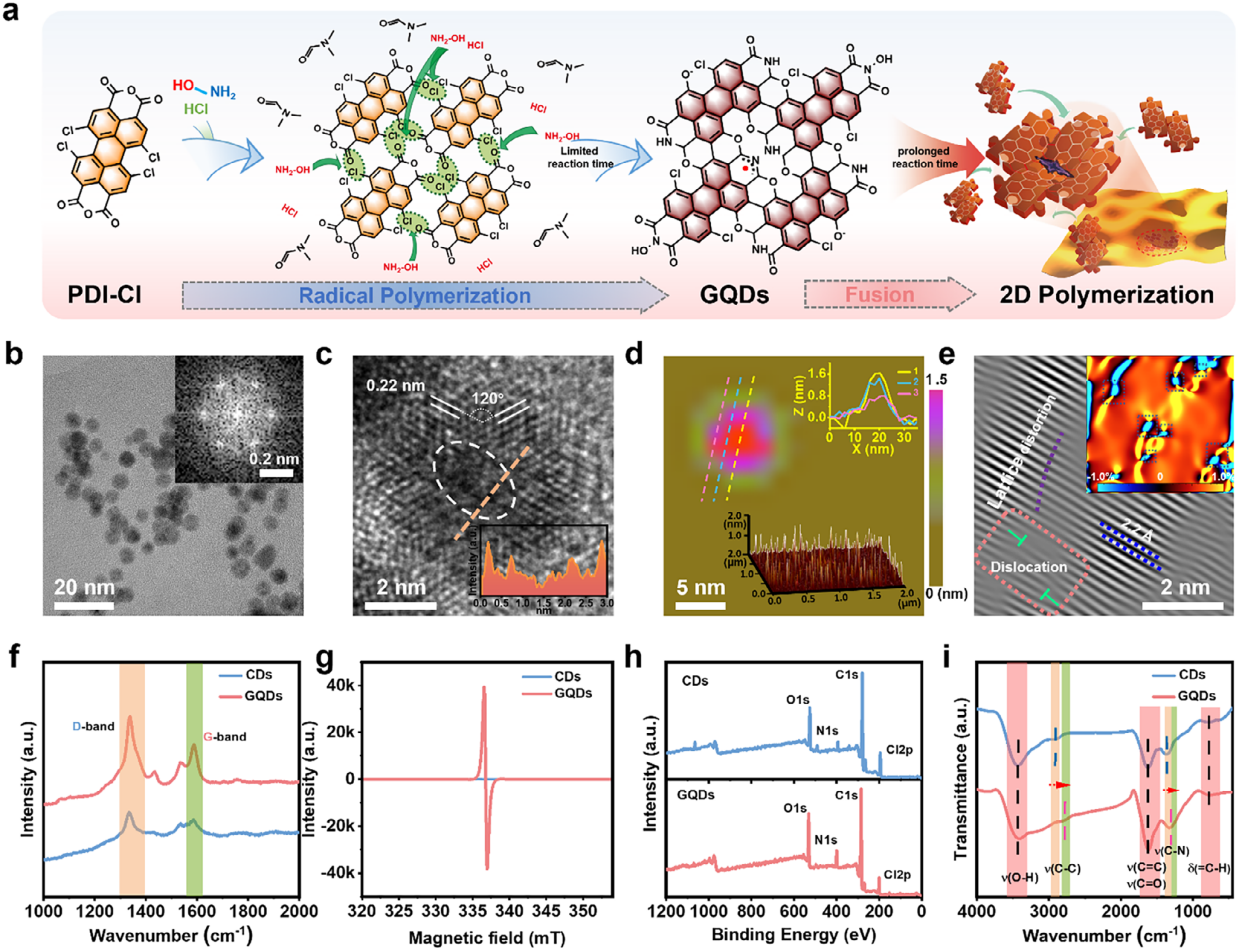
Synthesis mechanism, morphology, and structural characterizations. (a) Schematic illustration of the synthesis and possible formation mechanisms of GQDs. (b) TEM images of GQDs (inset: FFT of HRTEM image). (c) HRTEM images of GQDs, the white dashed lines denote defect sites (inset: Atomic intensity profile along the orange dashed line). (d) AFM images of GQDs (inset: the height profile along the lines and 3D maps of overall view). (e) IFFT patterns of HRTEM (inset: horizontal normal strain component (ε_xx_) derived from strain field mapping using geometric phase analysis (GPA)). (f) Raman, (g) EPR, (h) XPS, and (i) FT‐IR spectra of CDs and GQDs.

## 2 Results and Discussion

### 2.1 Morphology and Architecture of GQDs With Confined Radicals

The morphological and structural characterizations of GQDs were carried out by field emission transmission electron microscopy (TEM) and atomic force microscopy (AFM). TEM images indicated that GQDs had uniform diameters of 4–6 nm (Figure 1b and Figure S2). High‐resolution TEM (HRTEM) imaging of the GQDs revealed a concave interior surrounded by a continuous graphene‐like lattice, with a fringe spacing of ∼0.22 nm corresponding to the (100) plane of graphene (Figure 1c). AFM images of GQDs exhibited a central height of ∼1.3 nm that decreased radially to the edge, revealing a bowl‐like configuration with an apparent thickness of approximately one to two molecular layers (Figure 1d). Notably, we observed considerable lattice defects (including dislocations and vacancies) within the core regions of GQDs, consistent with the low crystallinity revealed by XRD, which were attributable to lattice distortions induced by heteroatom doping. Inverse fast Fourier transform (IFFT) analysis of these disordered lattice regions further identified numerous point and linear defects as the key cause of significant lattice geometric strain (Figure 1e and Figure S3). For comparison, the traditional CDs were prepared under the same method but without adding the hydroxylamine hydrochloride (Figure S1). The CDs exhibited polycrystalline graphene‐like core structures with diameters and heights of 6–8 nm, consistent with a spherical morphology (Figures S4–S6).

Raman spectra analysis indicated that the D bands (∼1350 cm^−1^) and G bands (∼1580 cm^−1^) for both types of CDs corresponded to the in‐plane vibrational modes of disordered sp^3^ domains and graphitized sp^2^ domains (Figure 1f), respectively [38]. GQDs displayed a higher D‐to‐G band intensity ratio (ID/IG = 1.25) than CDs (1.08). Meanwhile, the GQDs exhibited a strong electron paramagnetic resonance (EPR) signal at a g‐value of ∼2.004, arising from a high abundance of radicals (Figure 1g). In contrast, the EPR signal from CDs was negligible. These results collectively demonstrate that a high content of radicals can be realized via the rational design and fabrication of the GQDs.

To elucidate the chemical structure of the GQDs, X‐ray photoelectron spectroscopy (XPS), nuclear magnetic resonance (NMR) spectroscopy, and Fourier transform infrared (FT‐IR) spectroscopy were employed. XPS analysis revealed that the N atom contents of GQDs and CDs were approximately 7.5% and 4.9%, respectively, indicating that enhanced N‐doping in GQDs was facilitated by hydroxylamine hydrochloride (Figure 1h and Table S1). Compared to CDs, the high‐resolution C1s, N1s, O1s, and Cl2p spectra of the GQDs showed increased intensities for C─N (285.9 eV in C1s; 399.6 eV, and 400.4 eV in N1s) and N─N (401.4 eV in N1s) bonds (Figure S7). According to previous reports, the polymerization of perylene derivatives can proceed via one‐ or two‐dimensional (1D or 2D) growth [35, 36]. ^1^H, ^1^ ^3^C, and 2D NMR data reveal that upon introduction of hydroxylamine hydrochloride, the nucleophilic hydroxylamine activates the carbonyl groups on PDI‐Cl, initiating a condensation reaction between the carbonyl and chlorine atoms (Figures S8–S16). As the reaction proceeds, typical singlet signals corresponding to N–OH and NH groups are observed at 12.2, 11.1, and 9.6 ppm, respectively (Figures S10 and S16). Concurrently, ^1^H–^1^H correlations between C–H and imino protons observed in 2D COSY NMR, together with periodic shifts in the mass‐to‐charge (m/z) ratios of GQDs in mass spectrometry, further substantiate the two‐dimensional polymerization of PDI‐Cl into GQDs (Figures S12, S17, and S18). This process drives the formation and confinement of radicals within the core region, resulting in a unique lattice structure characterized by a disordered center and an ordered periphery (Figure 1c). In contrast, CDs exhibit disordered m/z peaks, which combined with lattice boundaries observed under TEM and irregular hexagonal FFT patterns, suggest a 1D polymerization of PDI‐Cl (Figures S4 and S19), followed by random assembly into spherical carbon cores [39, 40]. Moreover, as the reaction progresses, GQDs undergo isotropic expansion within the two‐dimensional plane (Figure S20), indicating their potential for achieving controllable periodic in‐plane radical confinement.

To further corroborate the presence of radicals at defect sites, EPR measurements were performed on monodisperse GQDs in DMSO, revealing a distinct hyperfine triplet splitting characteristic of nitrogen‐centered radicals. Combined with the elucidated chemical structure, this observation identifies these N‐radicals as originating from the product of carbonyl condensation at the carbon core center. Subsequent modulation of the proton environment and solvent–solute interaction network through methanol addition promotes exchange interactions (*H*_Ex_), leading to broadening of the radical signal into a single Lorentzian peak (Figure S21). Notably, this phenomenon was not observed for CDs. Thus far, the chemical structures of the CDs and GQDs have been clearly elucidated.

To elucidate the inner lattice strain in GQDs, we combined simulated atomic bond lengths with geometric phase analysis (GPA). As shown in a model of an optimized 2D polymerized PDI‐Cl segment using density functional theory (DFT), the bond lengths around O and N atoms in the graphene‐like structure exhibited pronounced elongation (>1.45 Å, marked in red), probably induced by the repulsion effect between localized radicals and non‐bonding electron pairs in the *π*‐conjugation system (Figure 2e) [41]. Consequently, the intrinsic lattice strain with intersecting dislocation sites was introduced in GQDs, forming symmetric compression‐tension strain pairs (GPA, Figure 1e inset) [42]. This considerable lattice strain in GQDs also induced redshifts in C─C/C─N stretching vibrations relative to CDs (FT‐IR, Figure 1i). Collectively, these analyses reveal that the 2D growth of periodically arranged PDI‐Cl units induces lattice distortions while stabilizing periodic radicals within the N, O co‐doped regions of the graphene‐like structure.

**FIGURE 2:**
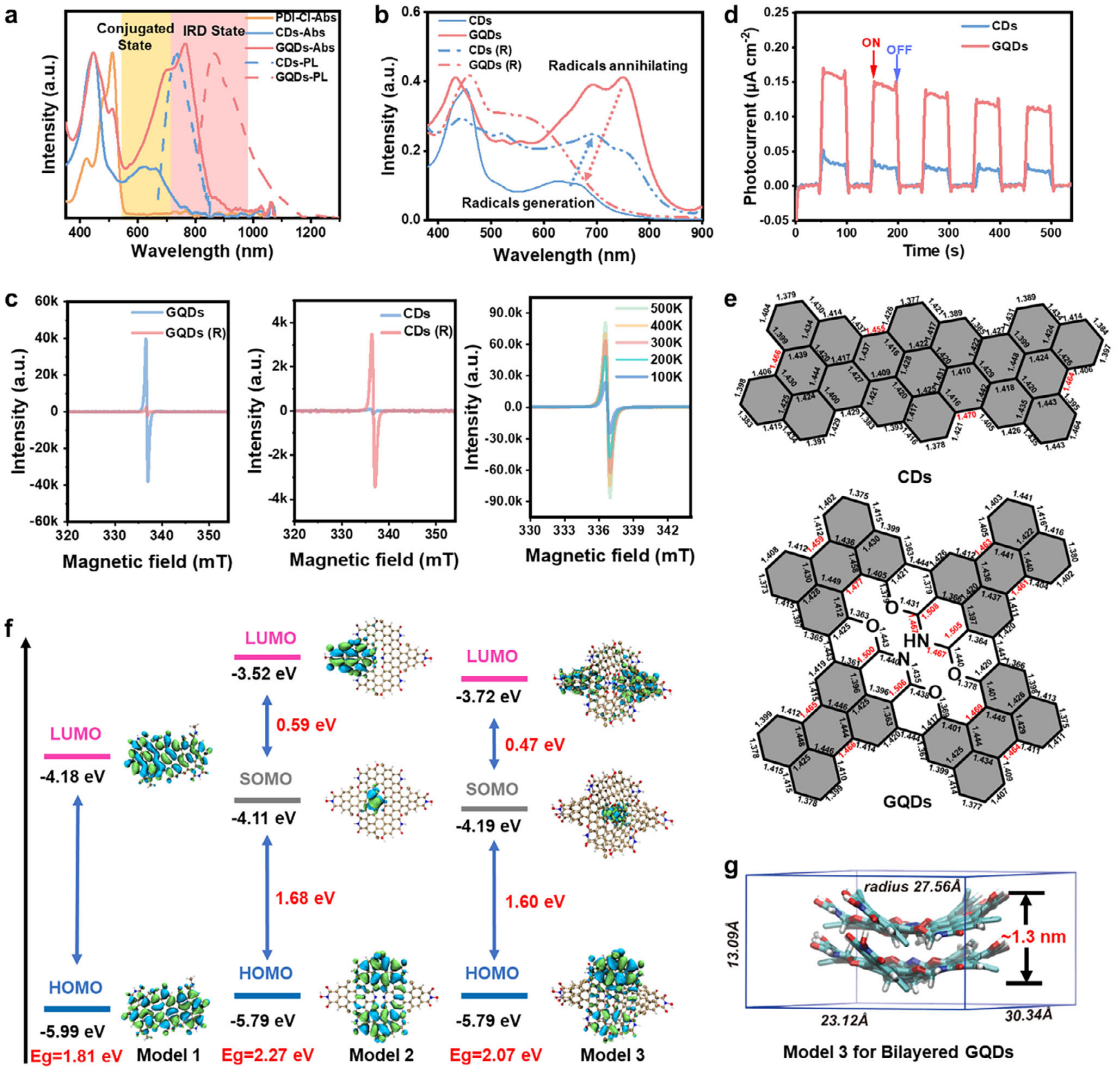
Optical properties characterization and simulations. (a) The absorption and NIR emission spectra of PDI‐Cl, CDs, and GQDs in DMSO. (b) Absorption spectra of CDs and GQDs before and after adding radicals via p‐chloranil treatment. (c) The EPR spectra of GQDs and CDs with and without adding radicals via p‐chloranil treatment, and temperature‐dependent EPR spectra of GQDs. (d) Photocurrent response of CDs and GQDs under 808 nm laser irradiation at 1 W cm^−2^. (e) DFT‐calculated bond lengths (Å) at conjugated topological interfaces (gray filled rings) in 1D polymerized PDI‐Cl (CDs) and 2D polymerized PDI‐Cl (GQDs). (f) Molecular structures and calculated HOMOs, SOMO, and LUMOs of simulated single‐layer 1D polymerized PDI‐Cl segment in CDs (model 1), simulatedsingle‐layerr 2D polymerized PDI‐Cl segment in GQDs (model 2), and simulated bilayer 2D polymerized PDI‐Cl segment in GQDs (model 3). (g) Simulated configuration of model 3 for bilayer GQDs.

### 2.2 Optical Properties and Dynamics

The photophysical characteristics of CDs and GQDs were comparatively analyzed through absorption and emission spectra. PDI‐Cl exhibited a molecular‐state absorption band centered at 500 nm (Figure 2a and Figures S22 and S23). CDs displayed an extended *π*‐conjugated absorption band from 550 to 780 nm. In contrast, GQDs showed diminished absorption at 450 nm and intensified broadband absorption from 600 to 1000 nm. Under 808 nm excitation, GQDs exhibited a primary emission at ∼855 nm with tailing to 1300 nm, demonstrating a ∼125 nm redshift relative to CDs, which peaked at 730 nm. Moreover, the high molar extinction coefficients and brightness ensured sufficient signal intensity during NIR‐II imaging (Figure S23 and Table S2). Based on HRTEM and EPR results, the long‐wavelength absorption beyond 700 nm might arise from the high‐content radicals rather than the extended sp^2^ domains.

To confirm the redshift in absorption and emission, a radical‐generation method was employed by treating with potassium t‐butoxide in tetrahydrofuran, followed by oxidation of the resulting carbanions with p‐chloranil [16]. The radical‐added CDs (CDs(R)) exhibited a new absorption band at about 700 nm, demonstrating that the emerging NIR absorption band originates from the incorporated radicals (Figure 2b). Conversely, the 750 nm absorbance of GQDs decreased upon introduction of external radicals, suggesting that radical annihilation might occur between the radicals in GQDs and external radicals from p‐chloranil treatment. These phenomena were further demonstrated by the diminished EPR signal of GQDs and enhanced EPR signal of CDs after introducing external radicals (Figure 2c). Furthermore, comparative ^1^H NMR analysis of GQDs and GQDs(R) elucidated that the integration of NH/N─OH increased after introducing external radicals, accompanied by near‐infrared fluorescence quenching (Figures S24 and S25). These observations support the rationality of the localization of radicals at core nitrogen sites within the carbon framework. Considering the radical absorption band in the NIR region as the main absorption band of GQDs, it is reasonable to assign it as intrinsic‐radical defect (IRD) state.

Notably, the intrinsic radical signal of GQDs remained stable over a broad temperature range from 100 to 500 K and increased with rising temperature, which is attributed to thermally promoted spin delocalization (Figure 2c). Consistent with this observation, GQDs retain comparable fluorescence stability to that of CDs even after prolonged storage under radical‐quenching conditions, such as in the presence of water and O_2_ (Figure S26). Quantitative EPR (qEPR) combined with SpinCount software showed that the absolute number of unpaired electron spins per GQD is approximately 0.7, indicating a high radical content in GQDs (Figure S27 and Table S3) [43]. These results further confirm that a large density of radicals was stabilized in GQDs during 2D polymerization of PDI‐Cls, facilitated by hydroxylamine hydrochloride.

CDs exhibited dominant green emission under 450 nm excitation with a PLQY of 21.20% (Figure S28 and Table S4). In contrast, GQDs exhibited multiple emission centers from green to red and NIR regions (Figure S29). GQDs showed red‐shifted and intensified NIR‐II emission with a high PLQY of 1.8% in DMSO at a concentration of 30 µg mL^−^^1^ under 808 nm excitation, whose integrated emission intensity in 900–1300 nm was 16.6‐fold higher than that of CDs under the same measurement conditions (Figure S30). It should be noted that the NIR emission of GQDs could be effectively excited in a wide spectral range from blue to NIR light, indicating effective energy/charge transfer occurs from the *π*‐conjugated domains to the intrinsic radical domains. This conclusion is strongly supported by the substantial photocurrent response observed in GQDs (Figure 2d) [44].

### 2.3 Quantum‐Chemical Simulations

The bandgap levels of CDs and GQDs were further investigated by XPS and solid‐state absorption spectra [45, 46]. The solid‐state absorption spectra indicated that the bandgaps of CDs and GQDs were 1.85 and 1.63 eV, respectively (Figures S31–S33). The gap narrowing is consistent with the observation of a redshift in the absorption of GQDs (Figure 2a). Meanwhile, the theoretical calculations using density functional theory (DFT) were also conducted. The geometrically optimized structures, as well as the distribution of lowest unoccupied molecular orbital (LUMO), highest occupied molecular orbital (HOMO), and singly occupied molecular orbital (SOMO) for three molecular structures in a vacuum were illustrated in Figure 2f and Figure S34. Model 2 for GQDs exhibited a larger HOMO‐LUMO gap than model 1 for CDs, which was likely due to restricted charge delocalization from disrupted *π*‐conjugation by the N, O dopants in the graphene‐like lattice (Figure S35) [47]. Meanwhile, we constructed a radical‐free molecular configuration of GQDs (model 4, radical‐induced bonding) to verify the bandgap for the conjugated domains. The calculation results showed that its HOMO and LUMO levels aligned closely with radical configuration of model 2 for GQDs, demonstrating that HOMO‐LUMO bandgap is strongly correlated with the conjugated domains in the overall framework (Figure S36). The emergence of SOMO level was attributed to the IRD state, yielding an inherently narrow bandgap with SOMO‐HOMO of 1.68 eV, which is narrower than the LUMO‐HOMO bandgap of model 1 for CDs (1.81 eV).

A bilayer 2D polymerized PDI‐Cl segment (model 3) for GQDs was also simulated [48]. This model exhibited a bowl‐like structure with a vertical height of ∼1.3 nm, which aligns well with the height of GQDs observed by AFM (Figure 2g and Figure S37). Simultaneously, the extended conjugated structure of CDs predominantly exhibited robust interlayer *π*–*π* interactions by interaction region indicator (IRI) analysis, whereas GQDs displayed weakened interactions due to the oscillatory configuration and spatial hindrance (Figure S38). DFT analysis of model 3 revealed reduced energy levels, potentially due to the spatial charge transfer effect between the graphene‐like layers. The LUMO‐HOMO and SOMO‐HOMO energy levels of model 3 for bilayer GQDs were decreased to 2.07 and 1.60 eV, respectively. Notably, the electron density of the SOMO level was still localized around the N, O‐doped areas in both model 2 and model 3 (Figure 2f).

### 2.4 Mechanisms of Exciton Dynamics

To elucidate the mechanism of exciton generation, diffusion, and dissociation, as well as excited‐state dynamics of radicals in the oscillatory graphene‐like structure, time‐correlated single‐photon counting (TCSPC), and ultrafast transient absorption (TA) spectroscopies were performed. The TCSPC results showed that CDs had a longer decay lifetime in green emission (5.05 ns) than that of PDI‐Cl (0.18 ns) and GQDs (2.42 ns) (Figure 3a). The shortened lifetime in GQDs is consistent with energy/charge transfer from the *π*‐conjugated domains to the intrinsic radical defects. In comparison with decay curves monitored at 540, 600, 700, 800, and 850 nm under 510 nm excitation, GQDs displayed asynchronous decay kinetics (Figure 3b). The prolonged rising edge of the 850 nm curve intersected the decaying edge of the 540 nm curve, indicating energy/charge transfer from the conjugation domains (green emission center) to the IRD domains (NIR emission center). The radiative recombination rate (k_r_) and non‐radiative recombination rate (k_nr_) were convoluted by τ such that τ^−1^ = k_r_ + k_nr_, which characterized the exciton transition (Table S5) [49]. The calculated k_r_ of 540 nm emissions from CDs and GQDs were determined to be 4.16 × 10^7^ and 3.72 × 10^5^ s^−1^, respectively. It can be inferred that the sharply declined k_r_ of GQDs (∼112‐fold) indicated the intrinsic radicals significantly suppressed radiative recombination in the conjugated emission center.

**FIGURE 3:**
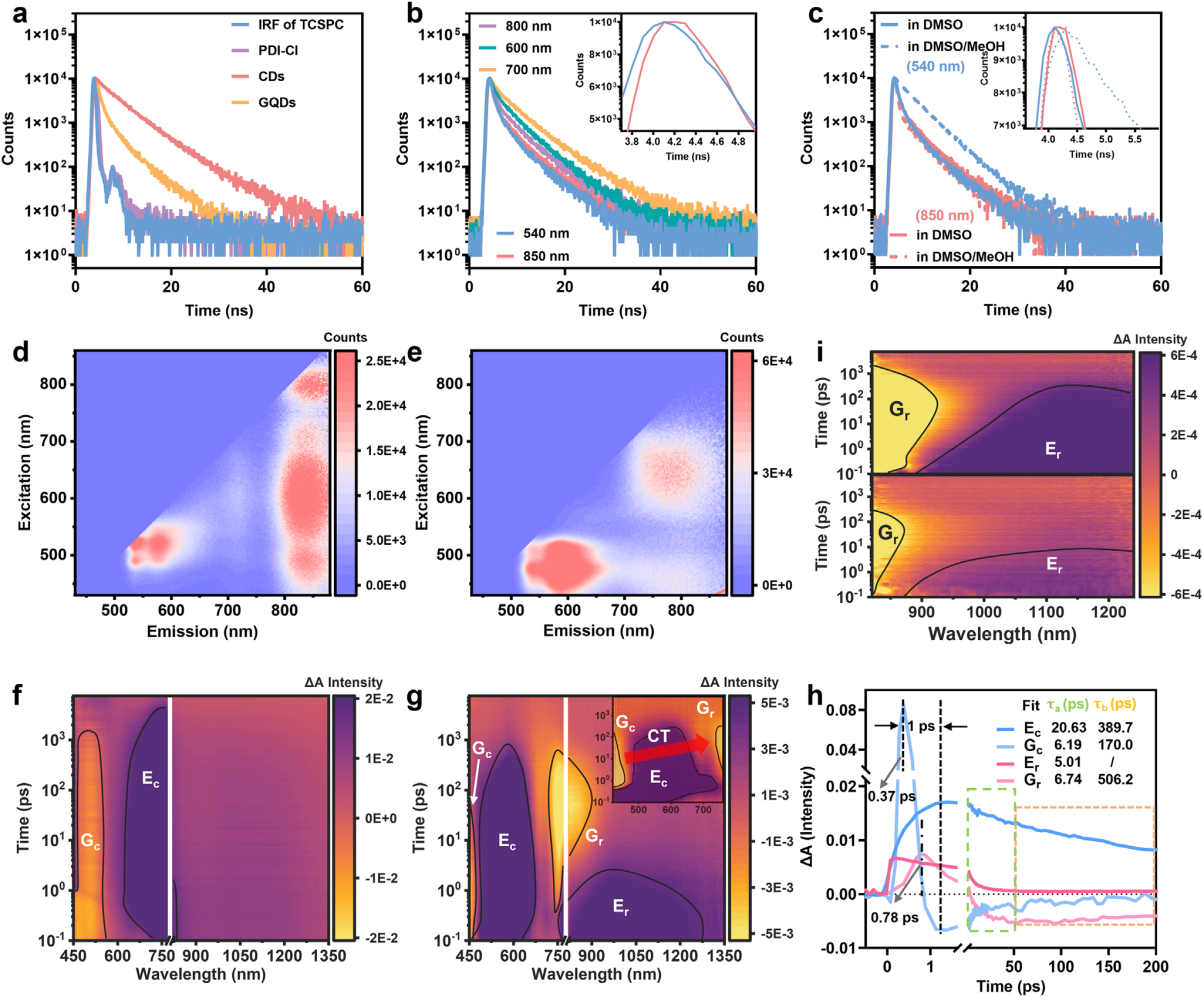
The investigation and characterization of photophysical properties. (a) Decay kinetics (excited at 510 nm and monitored at 540 nm; IRF: instrument response function) of CDs and GQDs in DMSO at 60 µg mL^−1^. (b) Decay kinetics (excited at 510 nm) of GQDs were monitored at 540, 600, 700, 800, and 850 nm in DMSO at 60 µg mL^−1^ (inset: local detail map on decay kinetics at 540 and 850 nm). (c) Decay kinetics (excited at 510 nm) of GQDs in DMSO and DMSO/methanol solutions at 60 µg mL^−1^ (inset: local detail map on decay kinetics). Excitation‐emission maps of GQDs in (d) DMSO solution and (e) DMSO/methanol solutions. 2D pseudo‐color maps of the TA spectra of (f) CDs and (g) GQDs at a pump wavelength of 400 nm (inset: TA spectra of (g) GQDs with a pump wavelength of 380 nm). (h)TA kinetic traces of GQDs collected at 440 nm (G_c_), 600 nm (E_c_), 760 nm (G_r_), and 970 nm (E_r_) at a pump wavelength of 400 nm. (i) 2D pseudo‐color maps of the TA spectra of GQDs at a pump wavelength of 700 nm in DMSO (top) and DMSO/methanol (bottom).

Then, methanol was introduced as a radical scavenger to further elucidate the inner charge transfer mechanisms. The decay curves of CDs displayed uniform decay kinetics, even in the presence of methanol treatment (Figure S39 and Table S6). However, fluorescence from GQDs exhibited prolonged lifetime at 540 nm in methanol solutions, indicating that the CT process was significantly impeded due to the radicals scavenged by methanol molecules (Figure 3c and Figures S40 and S41). Excitation‐emission maps of GQDs in methanol also indicated that the NIR emission was significantly weakened with narrowed excitation window (Figure 3d,e and Figure S42), indicating that exciton diffusion from the conjugated domains to the IRD domains was unfavorable in methanol.

In the TA spectrum of CDs, we assigned the negative signals at 450–550 nm and positive signals at 600–750 nm to ground‐state bleaching (GSB, G_c_) and excited‐state absorption (ESA, E_c_) from the conjugated state, respectively (Figure 3f and Figures S43–S45). In contrast, two bands of negative signals at 400–460 nm and 680–860 nm and two bands of positive signals at 470–660 nm and 750–1300 nm were observed in the TA spectrum of GQDs. Global fitting of GQDs reveals that the shorter wavelength GSB and ESA signals could be assigned from the conjugated state (Figure 3g and Figure S46). Interestingly, the longer wavelength GSB signal at 780 nm, which could be assigned from IRD state (G_r_), emerged with the decay of the G_c_ signal, indicating a charge transfer process that happened between G_c_ and G_r_. (Figure 3g inset and Figure S47) [50]. This CT process observed in TA corresponds to the growth time in TCSPC (Figure S48).

To investigate the exciton dissociation, we further fitted four traces (G_c_: 440 nm, E_c_: 600 nm, G_r_: 760 nm, and E_r_: 970 nm) of GQDs and two traces of CDs (E_c_: 500 nm, G_r_: 750 nm) (Figure 3h and Figure S49). Photoexcitation initially generated excitons (τ = 0.37 ps), followed by rapid dissociation (∼1 ps). Compared with the G_c_ signal in GQDs, the G_r_ signal at 0.78 ps was weak and likely originated from radical‐state exciton formation. In contrast, no such phenomenon was observed in the CDs. Subsequently, excited‐state dynamics were resolved into: (i) an ultrafast CT process (τ_a_, 2–40 ps) and (ii) a relatively slower exciton trapping process (τ_b_, 40–2000 ps), each fitted with single‐exponential decays. The decay lifetimes of G_c_ (6.19 ps) and G_r_ (6.74 ps) in GQDs were nearly identical, further demonstrating exciton diffusion process from the *π*‐conjugated domains to the IRD domains. Notably, GQDs exhibited prolonged dissociation times but accelerated relaxation times compared to CDs, potentially arising from their enlarged HOMO‐LUMO gaps and efficient exciton trapping by IRD domains (Figure 2f and Figure S49). This was corroborated by the shorter fluorescence lifetimes of GQDs observed in TCSPC measurements (Figure 3a). These findings were further verified by the significantly attenuated TA signals of radical states and accelerated lifetime decay observed under 700 nm excitation following methanol‐mediated radical scavenging (Figure 3i and Figure S50). Therefore, TA data elucidated the exciton dynamics of GQDs (generation → diffusion → trapping → recombination) under excitation, further illustrating that the effective CT between the *π*‐conjugated domains and the IRD domains drives pronounced near‐infrared emission [51].

Time‐dependent density functional theory (TD‐DFT) calculations and electron–hole analyses were conducted to reveal radical‐mediated excited dynamics, in which the D_index_ and Δσ index were employed to analyze the distribution of electrons and holes. Radical‐free model of GQDs (model 4) exhibited localized Frenkel excitons of electron density within conjugated domains (Figure 4a) [52, 53]. Meanwhile, its interrupted conjugation caused blue‐shifted absorption (460 nm), aligning with the experimental 450 nm peaks (Figures 2a and 4b). Radical‐containing model of GQDs (model 2), however, showed charge‐transfer‐dominated excitation (CTE) via HOMO‐1 → SOMO transitions (Figure 4c). Ground‐state electron density delocalized in edge conjugated domains, while excitation triggered exciton migration to radical cores (Movie S1 and Figures S51–S53). Benefiting from the stable radicals localized in periodic N, O‐doped graphene‐like structure, GQDs exhibited a significant redshift in the absorption spectrum (fitted peak: 661 nm, corresponding to the range at 600–1000 nm), corroborating radical‐generated NIR absorption/emission in GQDs (Figure 4d and Figure S54).

**FIGURE 4:**
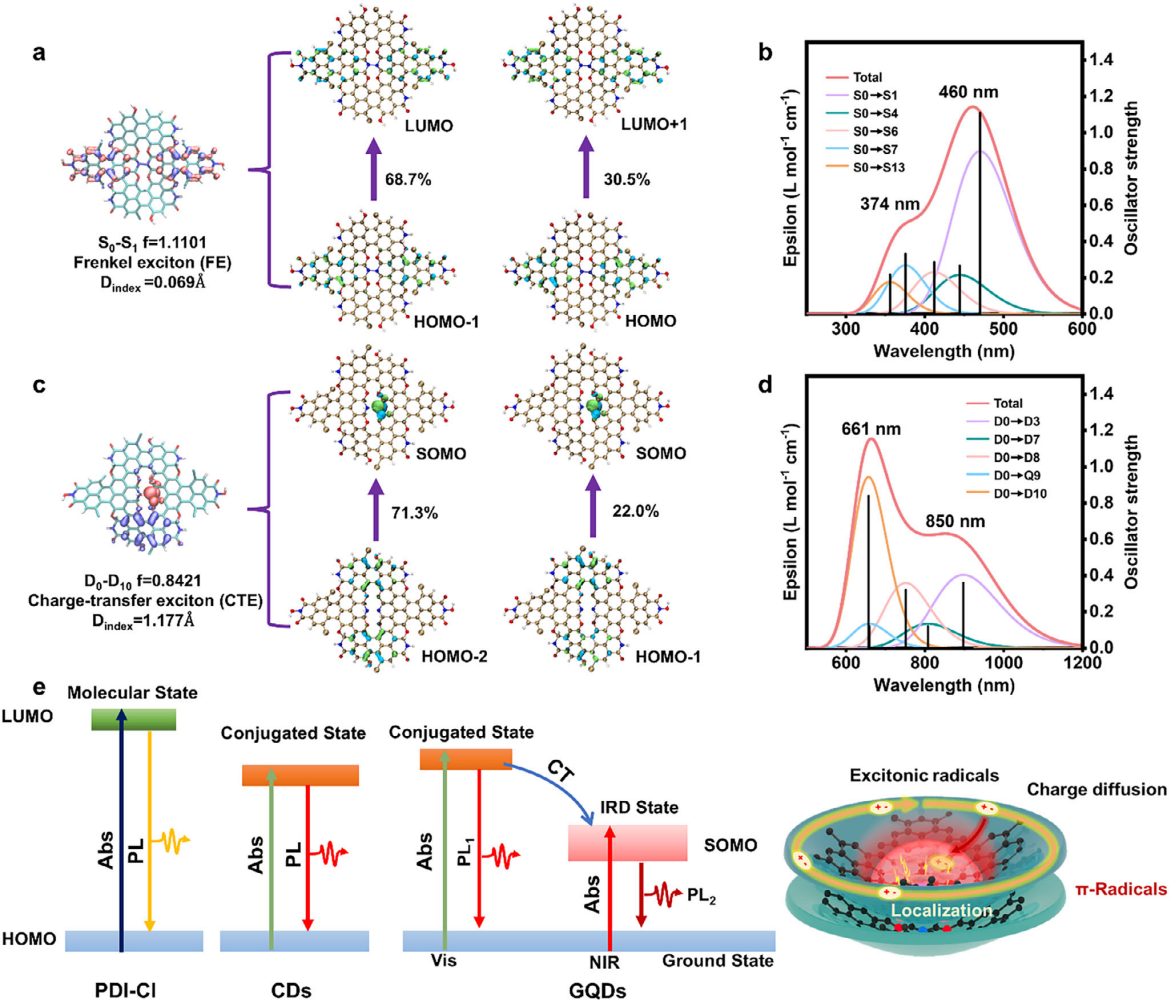
Radical excitation dynamics and mechanisms. Electron–hole distribution and orbital contributions of (a) model 4 (S_0_ → S_1_) and (c) model 2 (D_0_ → D_10_) of GQDs. Simulated absorption spectra and oscillator strength of (b) model 4 and (d) model 2 of GQDs. (e) Schematic diagram of the electronic transitions of GQDs in the excited state.

Hence, we propose a possible mechanism for the radical‐related NIR absorption and emission in GQDs (Figure 4e). PDI‐Cl can only be excited by visible light. Despite both CDs and GQDs have extended sp^2^ domains, large sp^2^ domains alone do not enable the absorption in the NIR region. For GQDs, the 2D polymerization of PDI‐Cl can form an oscillating bilayer graphene, in which intrinsic radicals localize within the periodic defects in a wide temperature range, leading to strong radical‐related absorption in the NIR region. These radicals serve as exciton trappers to facilitate a robust CT process: upon excitation, conjugated‐domain excitons dissociate into charge carriers, migrate to N, O‐defect sites in the graphene lattice and are trapped by radicals, resulting in excitonic‐radical emission in the NIR‐II region. These findings provide new ideas for studying the generation and relaxation dynamics of intrinsic radicals in periodic N, O‐doped graphene and establish an important theoretical basis for the construction and detection of thermodynamically stable radicals in semiconductor systems.

### 2.5 Real‐Time In Vivo NIR‐II Imaging

Considering that the radicals can be scavenged in proton solvents the NIR fluorescence from GQDs was significantly quenched in aqueous solution (Figure S55). Furthermore, GQDs exhibit good solubility only in common organic solvents, such as DMSO, but not in water (Figure S56). Thus, GQDs were further encapsulated with DSPE‐mPEG to prevent contact with water, forming GQD micelles with a size of approximately 50 nm (Figure 5a,b and Figure S57). With the help of DSPE‐mPEG, GQD micelles also displayed bright NIR‐II fluorescence in aqueous solution under 808 nm excitation (Figure 5c and Figure S58). Moreover, the NIR fluorescence from CDs, GQDs, and GQD micelles exhibited superior stability to that from commercial dye IR‐820 under irradiation with xenon lamp or 808 nm laser (Figure S59). The biocompatibility and biosafety assessments were performed through cytotoxicity, routine blood analysis, and Hematoxylin and eosin (H&E) staining. Negligible hemolysis in GQD micelles (< 5%) at 2 mg mL^−1^ indicated that they have a low impact on red blood cells (Figure S60). Moreover, there was no significant cytotoxicity in GQD micelles on L929 cells even up to 1.0 mg mL^−1^ (Figure S61). Then, we also evaluated biotoxicity of GQD micelles in vivo. H&E staining also suggested no remarkable damage and inflammatory lesions were observed in the organs of mice treated (Figure S62). Over 14 days, mice treated with GQD micelles at the imaging dose (1.0 mg mL^−^^1^) maintained stable body weight, comparable to that of the PBS‐treated control group (Figure S63). These observations demonstrated that GQD micelles have excellent biocompatibility.

**FIGURE 5:**
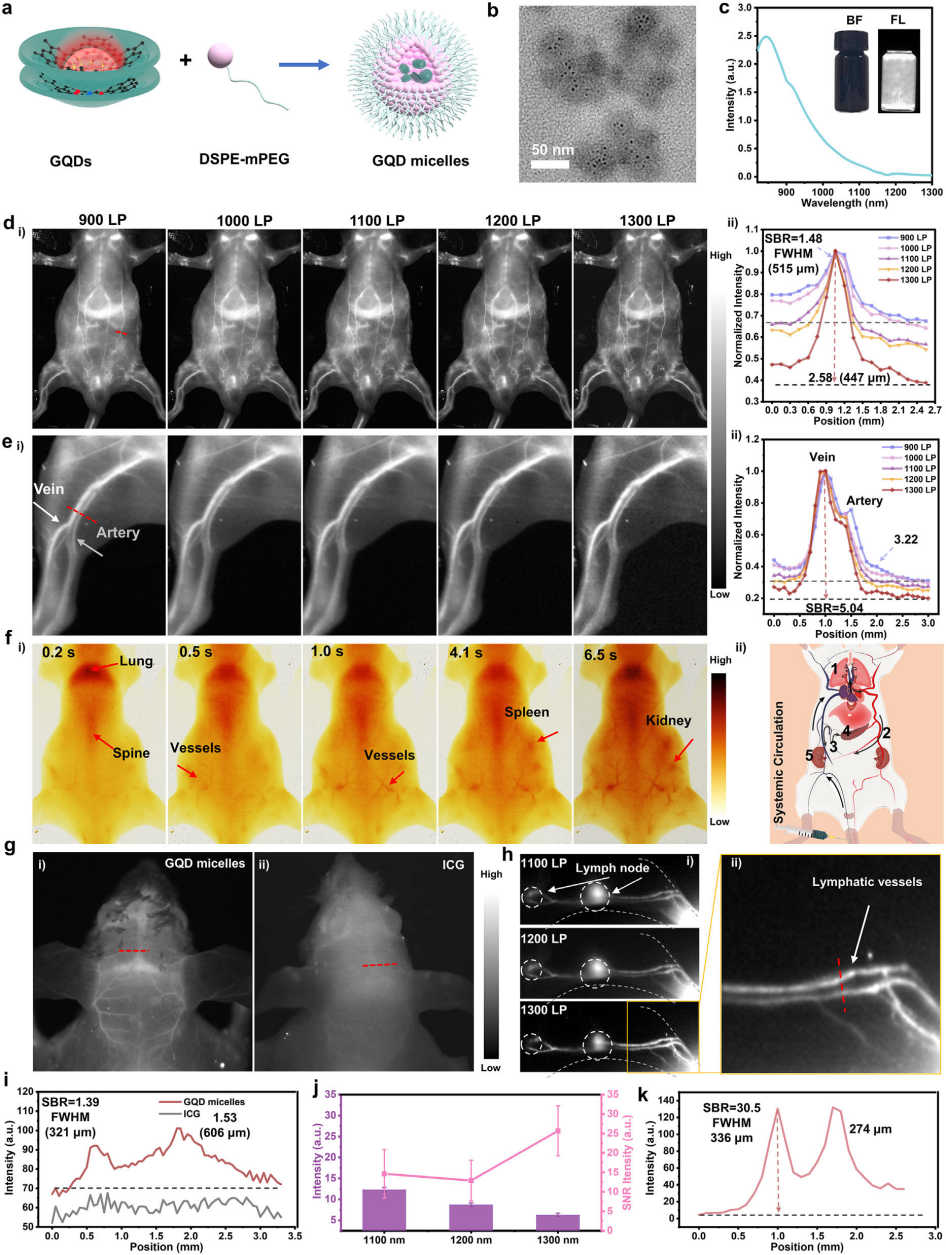
Synthesis mechanism and NIR­II fluorescence imaging. (a) Schematic illustration of the micelle formation process. (b) TEM image and (c) NIR fluorescence spectrum (inset: bright‐field and fluorescence image under 808 nm excitation) of the GQD micelles. (d) Supine view (i) NIR fluorescence image and (ii) fluorescence intensity profile along the red dashed line after intravenous injection of 100 µL GQD micelles (1.0 mg mL^−1^ in PBS) via the tail vein. (e) Mouse hindlimb (i) NIR fluorescence image and (ii) corresponding intensity profile. (f) The (i) Time‐lapse dorsal view imaging at representative time points and (ii) schematic diagram of metabolic clearance post‐injection (excitation: 808 nm, long‐pass filter: 1300 nm). (g) Cerebral fluorescence images after injection of (i) GQD micelles and (ii) ICG, respectively. (h) Lymph node (i) fluorescence images and (ii) magnified view of lymphatic vessels following footpad injection. (i) fluorescence intensity profiles along the red tangents of g. (j) Lymph node signal intensity and (k) fluorescence intensity profile along the tangent of the lymphatic vessel in (h).

Vascular networks play a crucial role in nutrient delivery and in sustaining the physiological integrity of organs throughout the body. High‐resolution angiography can enhance surgical precision by enabling clearer identification of vascular lesions across multiple organ systems and by providing reliable assessment of postoperative blood flow restoration [54, 55, 56]. Therefore, we performed in vivo angiography under 808 nm laser excitation using an indium‐gallium‐arsenide (InGaAs) camera, enabling high‐resolution imaging across the 950–1600 nm spectral range of the NIR‐II/SWIR window (Figure S64). The murine hindlimb and systemic vasculature were clearly delineated across a range of long‐pass (LP) filters (Figure 5d,e). Gaussian fitting of the cross‐sectional fluorescence intensity profiles demonstrated the superior performance of the 1300 nm LP filter over the 900 nm LP filter. This approach provided enhanced vessel delineation with suppressed background noise, yielding a 1.74‐fold improvement in the signal‐to‐background ratio (SBR) and a 68 µm reduction in the full width at half maximum (FWHM). The higher resolution provided by the 1300 nm LP filter enabled clear differentiation of arterial and venous vessels in the hindlimb. Furthermore, in contrast to the commercial dye indocyanine green (ICG), GQD micelles enabled clear, non‐invasive visualization of the cerebral vasculature through the intact skull and scalp (Figure 5g,i). This transcranial imaging capability achieved remarkable spatial resolution for microvessels, as evidenced by an FWHM of 76 µm. These results confirm the capability of GQDs for high‐precision vascular imaging and highlight their potential in the detection and tracing of vascular‐related diseases.

Pathophysiological changes in animals, such as abnormal vital signs, altered blood flow velocity, and fluctuations in tissue perfusion, often manifest rapidly and elude detection by conventional imaging modalities [57]. High‐frame‐rate (HFR) dynamic imaging enables the capture of these transient anomalies, facilitating real‐time diagnostic assessment. Utilizing GQD micelles, we achieved real‐time visualization of deep‐tissue dynamics in whole‐body murine models. In dorsal imaging, lung lobes were illuminated first (0.20 s post‐injection), followed by the spine (Figure 5f and Movie S2). Subsequently, mesenteric vasculature, spleen, and kidneys were visualized within 10 s, mapping the systemic circulation of GQD micelles. The majority of GQD micelles gradually accumulated in the liver within 1 h and entered intestinal metabolism at 3 h (Figure S65). Ex vivo tissue imaging at 12 h showed that most of the GQD micelles accumulated in the intestinal region, supporting their potential as real‐time imaging probes for colorectal‐related diseases. Collectively, radical‐engineered GQDs represented a promising platform for real‐time NIR‐II imaging and theranostics.

The reliable visualization of lymphatic and vascular systems is essential for assessing their physiological function and monitoring regenerative processes in biomedical research. This is particularly true for the lymphatic system, where the precise visualization and excision of lymphatic vessels (LVs) and lymph nodes (LNs) are of great significance in cancer detection and treatment. However, real‐time visualization of the complex lymphatic network in vivo remains challenging. The excellent fluorescent properties of GQDs allow for ultrahigh‐resolution imaging of LNs and LVs with remarkable SBR of 25.7 and 30.5, respectively (Figures 5h,j,k and S66), which was the highest among all CDs‐based lymph imaging. Furthermore, tumor imaging plays a critical role in both diagnosis and therapy, contributing to more effective cancer management and improved survival rates. Mice intravenously injected with GQD micelles showed peak tumor accumulation at 6 h, with a tumor‐to‐normal tissue (T/NT) ratio of 2.36, which is attributed to the enhanced permeability and retention effect (Figure S67). Ex vivo imaging of organs and tumors collected at 0.5, 3, 6, 12, and 24 h post‐injection confirmed that the maximum GQD accumulation occurred at 6 h (with tumor volumes ranging from 0.43 to 0.66 cm^3^ during the study period; Figures S68–S70), highlighting their potential for clinical application in tumor diagnosis.

## 3 Conclusion

In summary, we demonstrated a type of intrinsic radical through fabricating bowl‐like configuration of graphene quantum dots in a 2D polymerization way, achieving wide temperature range stability and excitonic‐radical NIR‐II emission with a PLQY of 1.8%. These ultrastable radicals were localized within periodically arranged defects in GQDs induced periodic distortions in a bilayer graphene‐like structure, generating intralayer oscillations that stabilized the radicals by minimizing interlayer interactions, thus contributing to a SOMO level with intrinsic‐radical defect (IRD) state in an enhanced absorption band exceeding 780 nm. Optical characterizations and theoretical calculations revealed exciton dissociation and diffusion from sp^2^ domains to intrinsic‐radical defects upon NIR excitation, achieving high excitonic‐radical emission in the NIR‐II region. The optimized emission from GQD micelles facilitates real‐time angiography of whole‐body vasculature, hindlimbs, cerebral structures, and lymph nodes, while further enabling fluorescence‐guided deep‐tissue dynamic imaging. Especially, GQD micelles exhibit more possibilities for remarkable spatial resolution of microvessels than commercial dye ICG.

The intrinsic radicals in GQDs are featured with simple synthesis and wide‐range temperature stability. We have performed extensive structural and chemical characterization for intrinsic radicals in GQDs. This comprehensive analysis confirms that the ultrastable radicals and their associated emission are well‐established. Notably, GQDs comprise a distribution of ordered, periodic homologous oligomers (an inherent characteristic of two dimensional polymerization) that share identical repetitive structural units and optical properties. This compositional heterogeneity does not detract from our central conclusion: the synthesis of an ultrastable, radical‐rich carbon nanostructure with defined chemical connectivity. This finding addresses key challenges associated with the highly reactive zigzag‐edge radicals typically found in traditional nanographene fabricated using AFM‐STEM techniques, enabling the scale synthesis of radical‐based nanographene and further advancing thermally‐stable NIR luminescent devices. These findings establish a valuable framework and offer inspiration for developing next‐generation radical semiconductor in photonics and spintronics. We anticipate this research will facilitate the development of 2D‐topology carbon‐based semiconductor systems featuring high‐density radicals and contribute to progress in the field of real‐time NIR‐II bioimaging.

## Author Contributions

D.G. and S.Q. planed and supervised the project. Q.X., Y.H., and D.G. designed and carried out the detailed experiments, participated in result analysis and wrote the whole paper. Y.H. and Q.X. prepared the materials and carried out the characterization measurements. B.W., T.Z, and G.X. assisted in the measurement and analysis of transient absorption spectra. Y.H., Q.X., S.L., and M.C. assisted with imaging in vivo. All authors participated in result analysis and wrote the whole paper.

## Conflicts of Interest

The authors declare no conflicts of interest.

## Supporting information




**Supporting File 1**: advs76970‐sup‐0001‐SuppMat.pdf.

**Supporting File 2**: advs76970‐sup‐0002‐MovieS1.avi.

**Supporting File 3**: advs76970‐sup‐0003‐MovieS2.avi.

## Data Availability

The data that support the findings of this study are available on request from the corresponding author. The data are not publicly available due to privacy or ethical restrictions.
