# Supplementary material for: Engineering Ultrastable Intrinsic Radicals: Graphene Quantum Dots With NIR‐II Emission for Dynamic Deep‐Tissue Bioimaging
Source: Adv Sci (Weinh). 2026 Aug 3;13(60):e76970. doi: 10.1002/advs.76970 (PMC13430622; doi:10.1002/advs.76970)
Supplement: Supplementary file 1 [file ADVS-13-e76970-s001.pdf]

Supporting Information

Engineering Ultrastable Intrinsic Radicals: Graphene Quantum Dots with NIR-II Emission for Dynamic Deep-Tissue Bioimaging

*Qin Xu[a, #], Yijie Hou[a, #], Bingzhe Wang[b], Tesen Zhang[c], Yupeng Liu[b], Shuaiqi Li[a], Maohua Chen[a], Guichuan Xing[b], Dongbo Guo[a, *], and Songnan Qu[b, *]*

[a]State Key Laboratory of Digital Medical Engineering, Sanya Research Institute of Hainan University, School of Biomedical Engineering, Hainan University, Sanya, 572025, China

[b]Joint Key Laboratory of the Ministry of Education, Institute of Applied Physics and Materials Engineering, University of Macau, Taipa, Macau SAR, 999067, China

[c]Interdisciplinary Institute of Medical Engineering, Fuzhou University, Fuzhou, 350108, China.

#These authors contributed equally: Qin Xu, Yijie Hou.

*Corresponding author. Email: dongboguo@hainanu.edu.cn (G. D); songnanqu@um.edu.mo (S. Q);

Table of Contents

1. **Materials and methods**·····1

2. **Structural and morphological analysis**·····11

3. **Fluorescence characterization**·····35

4. **Quantitative analysis of radicals**·····36

5. **Bandgap measurement**·····40

6. **Theoretical calculations**·····43

7. **Photophysical properties analysis**·····48

8. **Determination of exciton transfer**·····60

9. **Characterization of GQD micelles**·····64

10. ***In vivo* fluorescence characterization**·····72

11. **References**·····82

1. Materials and methods

Materials

1,6,7,12-tetrachloroperylene tetracarboxylic acid dianhydride (PDI-Cl), hydroxylamine hydrochloride, and N,N-dimethylformamide (DMF) were employed as starting materials for the synthesis of both types of carbon dots (CDs and GQDs). All commercial chemicals were of analytical grade and were used as received unless otherwise specified. All reactions sensitive to air or moisture were conducted under a nitrogen atmosphere using standard Schlenk techniques.

Characterization

Morphological characteristics and high dimensional size of nanoparticles can be achieved by using field emission transmission electron microscopy (Thermo Scientific Talos F200X G2) and atomic force microscopy (Bruker Dimension Icon), respectively. Raman spectra of the materials were obtained by high-resolution micro-confocal Raman spectroscopy (HORIBA LabRAM HR Evolution). NMR spectroscopy was performed on purified samples using a Bruker Avance NEO 400 MHz spectrometer. Visible and near-infrared photoluminescence spectra were obtained using spectrofluorometer (FLS1000 and Fluorolog-QM). Near-infrared fluorescence imaging of mice was performed using a bioimaging system (NIRvana HS) equipped with multi-channel long-pass filters, under a constant 808 nm laser power of 0.3 W cm-2. Electron paramagnetic resonance (EPR) spectroscopy measurements were carried out on a Bruker Magnettech ESR5000 spectrometer. All the animal experimental procedures were conducted according to the guidelines of the Regional Ethics Committee for Animal Experiments and the Care Regulations, and also approved by the Institutional Animal Care and Use Committee of the Hainan University (protocol NO. HNUAUCC-2023-00103). According to the guidelines, mice were sacrificed when the largest tumor dimension exceeded 1500 mm^3 (subcutaneous tumor model) or body weight loss attained 20% (orthotopic tumor model).

Theoretical simulation

The calculations were conducted in part of the high-performance computing cluster (HPCC) supported by the Information and Communication Technology Office (ICTO) of the University of Macau.

All calculations were performed on the Gaussian 16 package [1]. The constructed molecular model was subjected to density functional theory (DFT) calculations, and the B3LYP functional was used in conjunction with the D3(BJ) dispersion correction. Geometry optimization was performed using the 6-31G (d) basis set. The oscillator strengths from Time-dependent density functional theory (TD-B3LYP/cc-pVDZ) calculations for the transient species. The geometric optimization and reaction energy profile calculation on the dimer was performed using the same basis set as described above. The maps for electron density and electrostatic potential, distribution of hole-electron in the electron excitation process, and non-covalent interactions of the dimer was calculated using Multiwfn (version 3.7), and visualization by VMD software package [2, 3].

Transient Absorption (TA) measurement

Visible-near-infrared femtosecond transient absorption (TA) spectroscopy is used to probe photoexcited carrier dynamics and spectral evolution processes on ultrafast timescales. For the visible spectral range, the excitation light pulses are generated by a Coherent Astrella-1 K-F Ultrafast Ti: sapphire Amplifier (1 kHz, <100 fs) and captured by the Ultrafast System HELIOS TA spectrometer. The Vis-NIR TA measurements of the sample in DMSO were conducted in

a 1 mm optical path quartz cuvette. By using methanol as a radical scavenger (10% v/v), the charge carrier transfer processes were investigated.

Statistical analysis

The near-infrared fluorescence emission intensities of CDs and GQDs were normalized by Origin. The data were presented as mean ± SD. The statistical significance of differences among groups was assessed using the Student's t-test. A P value less than 0.05 was considered statistically significant for differences between experimental groups and control groups.

Data availability

The authors declare that the data supporting the findings of this study are available within the article and its Supplementary Information. Extra data are available from the corresponding authors upon request. Source data are provided with this paper.

NMR spectroscopy

The crude products were subjected to meticulous purification by column chromatography. ^{1}H and ^{13}C NMR spectra of the purified compounds were recorded on Bruker Avance NEO 400M spectrometer. Chemical shifts δ are given in parts per million (ppm) and J (coupling constants) in Hertz (Hz). For all multiplicities, the following abbreviations were used: s = singlet, d = doublet, dd = doublet of doublets, t = triplet, m = multiplet, br = broad. Solvent signals used for calibration of ^{1}H NMR: in deuterated Dimethyl sulfoxide (DMSO-*d6*) δ = 2.50 ppm, in H_2O δ = 3.33 ppm. Solvent signals used for ^{13}C NMR: in DMSO-*d6* δ = 77.2 ppm.

Fluorescence characterization

All fluorescence spectra were tested in organic solutions, except for researching the influence of mixed solvents on fluorescence intensity.

The visible and near-infrared Steady-state fluorescence spectra were measured using the 920 and InGaAs detectors of Fluorolog-QM, respectively. The fluorescence stability (30 µg mL^{-1}, DMSO) was verified by detecting the fluorescence intensity changes of the sample solutions after irradiation with a xenon lamp (500 W m^{-2}) and an 808 nm laser (1 W cm^{-2}). Meanwhile, we systematically investigated the fluorescence properties of the materials across a gradient of solvent polarities. Fluorescence imaging of all samples under 450, 675, and 808 nm excitation at various exposure times and with different long-pass filters (540, 740, and 840 nm) was collected using a bioimaging system, respectively.

Integrating sphere-based photoluminescence quantum yield (PLQY, Φ_{fl}) determination: The PLQY in the visible and near-infrared (NIR) regions were calibrated using rhodamine B (RhB, Φ_{fl} = 70% in ethanol) and new indocyanine green (IR-820, Φ_{fl} = 4.1% in ethanol) as reference standards, respectively. A 3 mL aliquot of the sample solution in DMSO and an equivalent volume of pure DMSO (solvent blank) were sequentially loaded into the Fluorolog-QM integrating sphere. Emission and excitation spectra were recorded, and Φ_{fl} was calculated using equation.

$$\Phi_{fl} = \frac{I_{em} - I_{ref2}}{I_{ex} - I_{ref1}} \times 100\% \qquad \text{Eq. (S1)}$$

Where I_{em} is the integral value of the fluorescence spectrum of the sample solution, and I_{ref2} is the integral value of the fluorescence spectrum of the pure solvent in this range. I_{ex} is the integral value of the excitation band of the sample solution, and I_{ref1} is the integral value of the pure solvent excitation spectrum in this range. The photoluminescence quantum yield of the sample can be obtained by calculation.

Fluorescence lifetime measurement

The fluorescence lifetime was recorded using an Edinburgh Instruments spectrofluorometer (FLS1000). Upon excitation with a pulsed diode light source (NanoLED), the time-correlated single photon counting (TCSPC) signals of the sample and their precursors (PDI-Cl) at different emission wavelengths were collected and an instrument response function (IRF) determined with a Ludox silica particle.

The decay lifetimes collected at each emission wavelength can be described by the following exponential decay formula (ExpDec).

$$R_{(t)} = B_1 e^{\left(-\frac{t}{\tau_1}\right)} + \cdots + B_n e^{\left(-\frac{t}{\tau_n}\right)} \quad (n \text{ takes 2 or 3}) \qquad \text{Eq. (S2)}$$

Among them, $R_{(t)}$ is the fluorescence intensity as a function of time, t is the time parameter of the instrument test, τ_n is the fluorescence lifetime of the nth decay component and B_n is the fractional amplitude of that component.

According to the fitting of each fluorescence lifetime, different fitting parameters are selected so that its confidence parameter R-square is within the range of 0.8-1.3, and the average fluorescence lifetime of the tested material is calculated by the following equation.

$$\tau = \frac{B_1\tau_1^2 + \cdots + B_n\tau_n^2}{B_1\tau_1 + \cdots + B_n\tau_n} \quad (n \text{ takes 2 or 3}) \qquad \text{Eq. (S3)}$$

Electron Paramagnetic Resonance (EPR) detection

Electron Paramagnetic Resonance (EPR) spectroscopy is a well-known quantitative magnetic analytical method for free radicals. By detecting the spin-flip absorption signals of unpaired electrons under excitation by electromagnetic radiation of specific frequencies, this technique elucidates the microscopic magnetic properties of materials. The intrinsic spin magnetic moments of unpaired electrons within radical molecules enable their effective detection and precise quantification via EPR.

5 mg of the powdered sample was loaded into an EPR quartz tube. The radical species and their relative intensities within the material were analyzed using an X-band (9-10 GHz) EPR spectrometer. The radical concentration in the powdered GQDs samples was quantified using quantitative EPR (qEPR) combined with SpinCount software for spectral fitting[4]. In the qEPR measurements, 2,2,6,6-tetramethylpiperidinyl-1-oxide (TEMPO) with a known radical concentration was employed as the standard reference. The absolute number of unpaired electron spins in the samples was determined by analyzing the EPR signal amplitudes. The mass fraction of radicals in the GQDs samples was directly calculated from the ratio of the peak areas between the sample and the standard, eliminating the need for empirical equation calibration. The simplified qEPR equation used in this study is expressed as:

$$n_s = \frac{n_p A_s}{A_p} \qquad \text{Eq. (S4)}$$

Where ns and np represent the spin numbers of the test sample (GQDs) and the standard (TEMPO), respectively, while As and Ap denote their corresponding EPR peak areas. This equation is modified as follows:

$$x_s = x_p \frac{A_s W_p M_s}{A_p W_s M_p} \qquad \text{Eq. (S5)}$$

In the equations, x_s and x_p denote the purity of the test sample (GQDs) and the standard sample (TEMPO), respectively. Ws and W_p represent their weights, A_s and A_p correspond to the integrated EPR peak areas of the test sample and the standard sample, respectively. M_s and M_p are their molar masses. Additionally, the spin number in the sample was directly calculated using the SpinCount software and cross-validated against the qEPR results. Furthermore, the temperature-dependent in situ radical signals of GQDs were systematically investigated across a broad temperature range (100-500 K). This was achieved through precise thermal regulation

using a control system equipped with a liquid nitrogen reservoir and a nitrogen dewar for stable measurements.

To resolve the hyperfine splitting of the radicals, electron paramagnetic resonance (EPR) measurements were conducted on samples dispersed in 1 mL of DMSO containing 100 μg of the material. The transition from hyperfine splitting to spin-exchange averaging was captured by the addition of 5% v/v anhydrous methanol to the solvent.

Transient Photocurrent (TPC) measurement mechanism

Transient Photocurrent analysis is an effective method for detecting instantaneous current responses induced by photoexcitation-induced carrier transport in materials, enabling the evaluation of charge separation and migration dynamics. Photoelectrochemical measurements were performed using a commercial electrochemical workstation (CHI 760F) with a standard three-electrode configuration, comprising four principal components: an excitation light source, photodetector, preamplifier, and digital signal processor. The electrolyte consisted of acetonitrile containing 0.1 M tetrabutylammonium hexafluorophosphate ((n-Bu)4NPF6) as supporting electrolyte. Indium tin oxide (ITO) glass served as the working electrode, while Ag/Ag^+ and platinum foil acted as the reference and counter electrodes, respectively. Excitons dissociate into charge carriers that migrate from edge states to central radical species. In organic semiconductors, TPC signal intensity correlates positively with photogenerated carrier density, providing a quantitative measure of charge transfer efficiency and interfacial carrier dynamics.

Electronic excitation and relaxation in radicals

Excited-state exciton dynamics in the radical model were analyzed using the hole-electron correlation and natural transition orbital (NTO) modules integrated into the Multiwfn software[5,6]. Photophysical characteristics, including absorption and emission behavior, were evaluated by examining the five lowest-energy excited states.

Hole-electron analysis, which characterizes electronic excitation as a hole-to-electron transition, was evaluated by graphical representations to spatially resolve differences between hole and electron distributions. This enables direct identification of excitation mechanisms, such as localized, collective, charge-transfer, or hybridized processes.

For TDDFT, the hole and electron are expressed as follows:

$$\rho^{hole}(r) = \rho^{hole}_{(loc)}(r) + \rho^{hole}_{(cross)}(r) \quad \text{Eq. (S6)}$$

$$\rho^{hole}_{(loc)}(r) = \sum_{i \to a} (w_i^a)^2 \varphi_i \varphi_i - \sum_{a \to i} \left(w'^a_i\right)^2 \varphi_i \varphi_i \quad \text{Eq. (S7)}$$

$$\rho^{hole}_{(cross)}(r) = \sum_{i \to a} \sum_{j \neq i \to a} w_i^a w_j^a \varphi_i \varphi_j - \sum_{a \to i} \sum_{j \neq a \to i} w'^a_i w'^a_j \varphi_i \varphi_j \quad \text{Eq. (S8)}$$

$$\rho^{ele}(r) = \rho^{ele}_{(loc)}(r) + \rho^{ele}_{(cross)}(r) \quad \text{Eq. (S9)}$$

$$\rho^{ele}_{(loc)}(r) = \sum_{i \to a} (w_i^a)^2 \varphi_a \varphi_a - \sum_{a \to i} \left(w'^a_i\right)^2 \varphi_a \varphi_a \quad \text{Eq. (S10)}$$

$$\rho^{ele}_{(cross)}(r) = \sum_{i \to a} \sum_{i \to b \neq a} w_i^a w_j^a \varphi_a \varphi_b - \sum_{a \to i} \sum_{b \neq a \to i} w'^a_i w'^a_j \varphi_a \varphi_b \quad \text{Eq. (S11)}$$

Here, r denotes the coordinate vector, φ represents the orbital wavefunction, i or j are occupied orbital index, and a or b are unoccupied orbital indices. Thus, the summation over $i \to a$ enumerates all electronic excitation configurations, whereas the summation over $a \to i$ corresponds to de-excitation configurations. The hole distribution ρ^{hole} and the electron distribution ρ^{ele} are decomposed into localized and cross-configurational contributions to quantify spatial charge transfer dynamics.

The contribution of a molecular orbital pair to the electron and hole is defined as follows:

$$\Theta_i^{hole} = \sum_a \left[(w_i^a)^2 - \left(w'^a_i\right)^2\right] \quad \text{Eq. (S12)}$$

$$\Theta_i^{ele} = \sum_i \left[(w_i^a)^2 - \left(w'^a_i\right)^2\right] \quad \text{Eq. (S13)}$$

Where a and i are orbital indices.

The distance between the hole and electron centroids (the points of most central distribution of the electron wavefunction overall) is quantified using the D_{index}:

$$D_X = |X_{ele} - X_{hole}| \quad D_Y = |Y_{ele} - Y_{hole}| \quad D_Z = |Z_{ele} - Z_{hole}| \quad \text{Eq. (S14)}$$

$$D_{index} = \sqrt{(D_X)^2 + (D_Y)^2 + (D_Z)^2} \quad \text{Eq. (S15)}$$

The term X_{hole} corresponds to the X-coordinate of the hole centroid, derived by integrating the product of the hole density distribution function (ρ^{hole}) and the X-coordinate variable over the entire three-dimensional space. The difference between the excited-state and ground-state dipole moments can be determined directly by computing the spatial separation between the hole and electron centroid positions.

Animals and tumor model

All BALB/c mice, aged 5 weeks, were purchased from SPF (Beijing) biotechnology Company Limited. A subset of mice received a subcutaneous injection of 20 μL of 4T1 cell suspension (RRID: CVCL_0125; 5×10^7 cells mL^{-1}) into the dorsal flank to establish a tumor-bearing model. Upon reaching a tumour volume of approximately 500 mm^3, the mice were subjected to experimental procedures.

***In vivo* vascular and tumor imaging**

In vivo imaging was performed on 5- to 7-week-old depilated BALB/c mice. For systemic vasculature imaging, mice were intravenously injected with GQD micelles (100 μL, 1 mg/mL) via the tail vein and imaged using a NIRvana HS bioimaging system. Time-lapse images were acquired under 808 nm laser excitation (0.3 W/cm^2) with long-pass filters set at 900, 1000, 1100, 1200, and 1300 nm. Dynamic video recording was initiated simultaneously with injection to capture the real-time circulation. Tracking of the popliteal and sciatic lymph nodes, along with the observation of inter-nodal drainage, was achieved by footpad injection of GQD micelles. To benchmark the imaging performance, the commercial NIR dye ICG was used for comparative brain imaging in mice.

The 4T1 tumor-bearing BALB/c mice were used as an animal model to investigate biodistribution through *in vivo* and *ex vivo* imaging. The GQD micelles (100 μL, 1 mg mL^{-1}) were intravenously injected into 4T1 tumor-bearing BALB/c mice. Then, fluorescence images (excitation: 808 nm, long-pass filter: 1100 nm) of mice were collected at various time points using a bioimaging system (NIRvana HS). For *ex vivo* imaging, another group of tumor-bearing BALB/c mice was intravenously injected with the GQD micelles. Mice were euthanized at different time points to retrieve their heart, liver, spleen, lung, kidney, and tumor. The bioimaging system was used to capture images. Throughout the experiments, none of the animals exhibited any indications of acute toxicological effects. The fluorescence intensity was analyzed using ImageJ software.

Statistical analysis

The near-infrared fluorescence emission intensities of CDs and GQDs were normalized by Origin. The data were presented as mean ± SD. The statistical significance of differences among groups was assessed using the Student's t test. In all cases, significance was defined as $p \leq 0.05$.

Synthesis details and Characterizations

In this study, 1,6,7,12-tetrachloroperylene tetracarboxylic acid dianhydride (PDI-Cl) was used as the precursor to synthesize two types of graphene quantum dots (CDs and GQDs) without or with hydroxylamine hydrochloride.

Synthesis for CDs

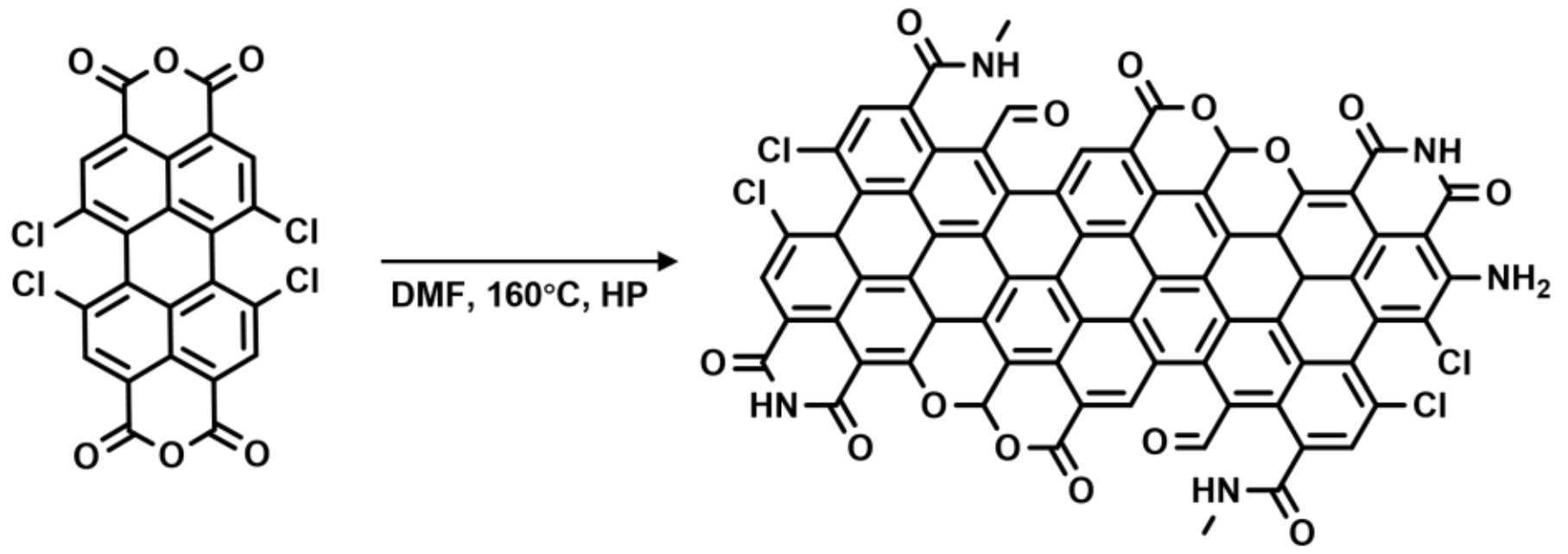

In a typical synthesis of carbon dots, PDI-Cl (0.1 g, 0.19 mmol, MW = 530.1 g·mol^{-1}) was dissolved in 30 mL of DMF under ultrasonication for 10 min to form a clear precursor solution. The solution was then transferred into a 50 mL Teflon-lined autoclave and heated a constant temperature of 160 °C for 5 h. After the reaction, the resulting mixtures were purified by dialysis against deionized water, followed by lyophilization. Further purification was carried out by C18 reverse-phase silica gel column chromatography using a mixture of dichloromethane and methanol (5:1, v/v) as the eluent. The collected product fractions were concentrated via rotary evaporation to to yield the final CDs products. ^{1}H NMR (400 MHz, DMSO) δ = 9.14 (t, J = 9.3 Hz, 1H), 9.05 (d, J = 9.6 Hz, 2H), 8.56 (s, 2H), 8.45 (d, J = 19.3 Hz, 1H), 8.17 (d, J = 12.4 Hz, 3H), 7.97 (d, J = 15.3 Hz, 3H), 7.91 (d, J = 8.5 Hz, 1H), 7.85 (d, J = 9.6 Hz, 2H), 3.43 (s, 1H), 3.13 (d, J = 27.1 Hz, 2H), 2.90 (d, J = 10.9 Hz, 3H), 2.74 (d, J = 6.1 Hz, 1H), 1.22 (s, 2H), 0.82 (s, 1H). ^{13}C NMR (101 MHz, DMSO) δ 177.03, 167.57, 159.19, 134.76, 132.75, 131.21, 129.73.

Synthesis for GQDs

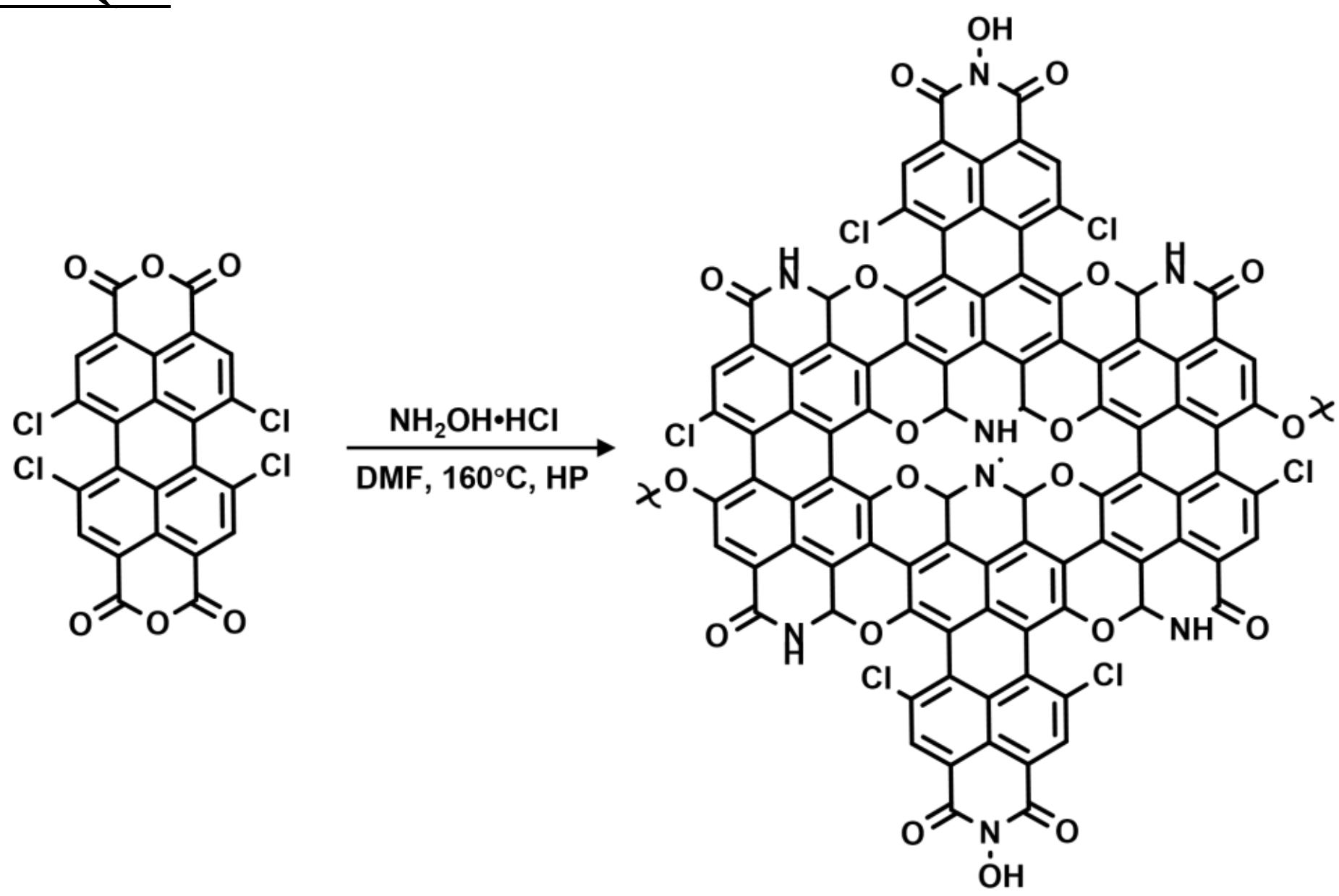

For GQD synthesis, PDI-Cl (0.1 g, 0.19 mmol, MW = 530.1 g·mol^{-1}) and hydroxylamine hydrochloride (0.1 g, 1.44 mmol, MW = 69.49 g mol^{-1}) was dissolved in 30 mL of DMF under ultrasonication for 10 min to form a clear precursor solution. The solution was then transferred into a 50 mL Teflon-lined autoclave and heated a constant temperature of 160 °C for 5 h. After the reaction, the resulting mixtures were purified by dialysis against deionized water, followed by lyophilization. Further purification was carried out by C18 reverse-phase silica gel column chromatography using a mixture of dichloromethane and methanol (5:1, v/v) as the eluent. The collected product fractions were concentrated via rotary evaporation to yield the final GQDs products. ^{1}H NMR (400 MHz, DMSO) δ = 12.19 (s, 2H), 11.12 (s, 7H), 9.59 (s, 1H), 8.58 (s, 8H), 8.51 (s, 4H), 8.41 (d, J = 16.4 Hz, 1H), 8.35 – 8.25 (m, 1H), 7.99 (d, J = 22.6 Hz, 1H), 7.56 (d, J = 28.3 Hz, 1H), 3.73 (s, 1H), 3.13 (d, J = 25.3 Hz, 5H), 2.93 (d, J = 36.5 Hz, 1H), 2.78 – 2.57 (m, 2H), 2.33 (s, 1H), 1.22 (s, 2H), 0.85 (s, 1H), 0.06 (s, 1H). ^{13}C NMR (101 MHz, DMSO) δ 162.48, 159.36, 134.23, 131.61, 130.92, 127.78, 124.13.

To gain insight into the GQD formation mechanism, we monitored the evolution of the products by employing different reaction times (1, 3, and 10 h). The corresponding products were then isolated and analyzed. With the exception of the formation mechanism investigation, all studies regarding the properties of the GQDs and the related mechanistic explorations were conducted using the purified product obtained after a 5 h reaction.

Synthesis for CDs(R) and GQDs(R)

The synthesis of radicals from precursors has been previously reported in the literature[6,7]. Both CDs(R) and GQDs(R) were prepared according to a general procedure for radical generation in carbon-based materials.

The typical protocol is described as follows: under an argon atmosphere and protected from light, the carbon quantum dot sample (10.0 mg) was dissolved in anhydrous tetrahydrofuran (THF, 40 mL). An excess amount of potassium tert-butoxide (10.0 mg) was then added, upon which the solution immediately turned wine-red. The reaction mixture was stirred at room temperature in the dark for 2 hours, followed by the addition of p-chloranil (20.0 mg). After stirring for another 2 hours, the reaction was quenched. The solvent was removed under reduced pressure, and the crude product was purified by reversed-phase silica gel column chromatography using a dichloromethane/methanol mixture (5:1, v/v) as the eluent. The final product was obtained as a solid powder after solvent evaporation under vacuum.

2. Structural and morphological analysis

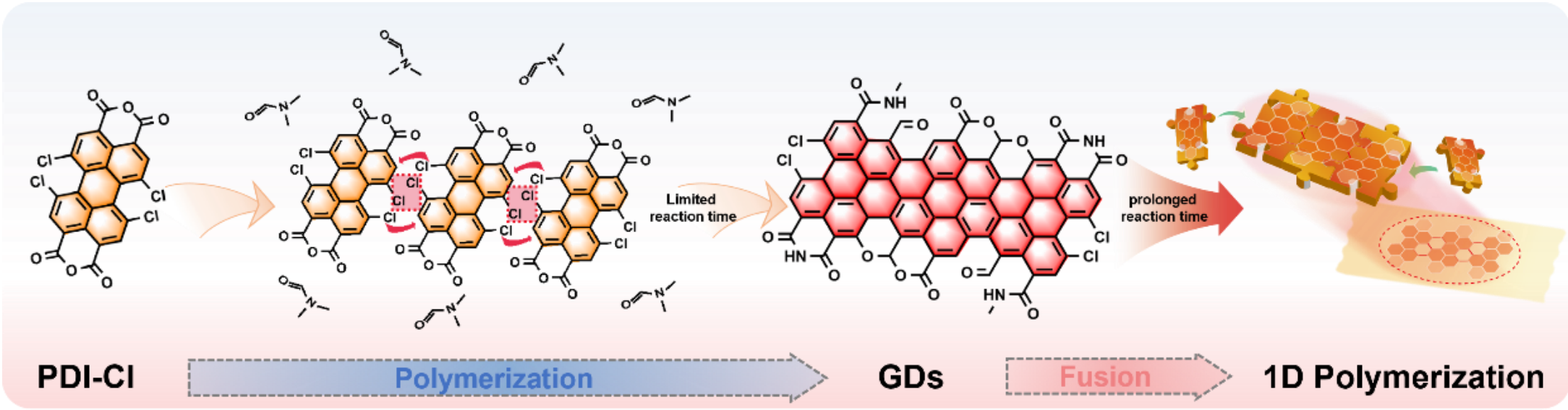

Figure S1. Schematic of the synthesis of CDs. Schematic illustration of the synthesis and possible formation mechanisms of CDs.

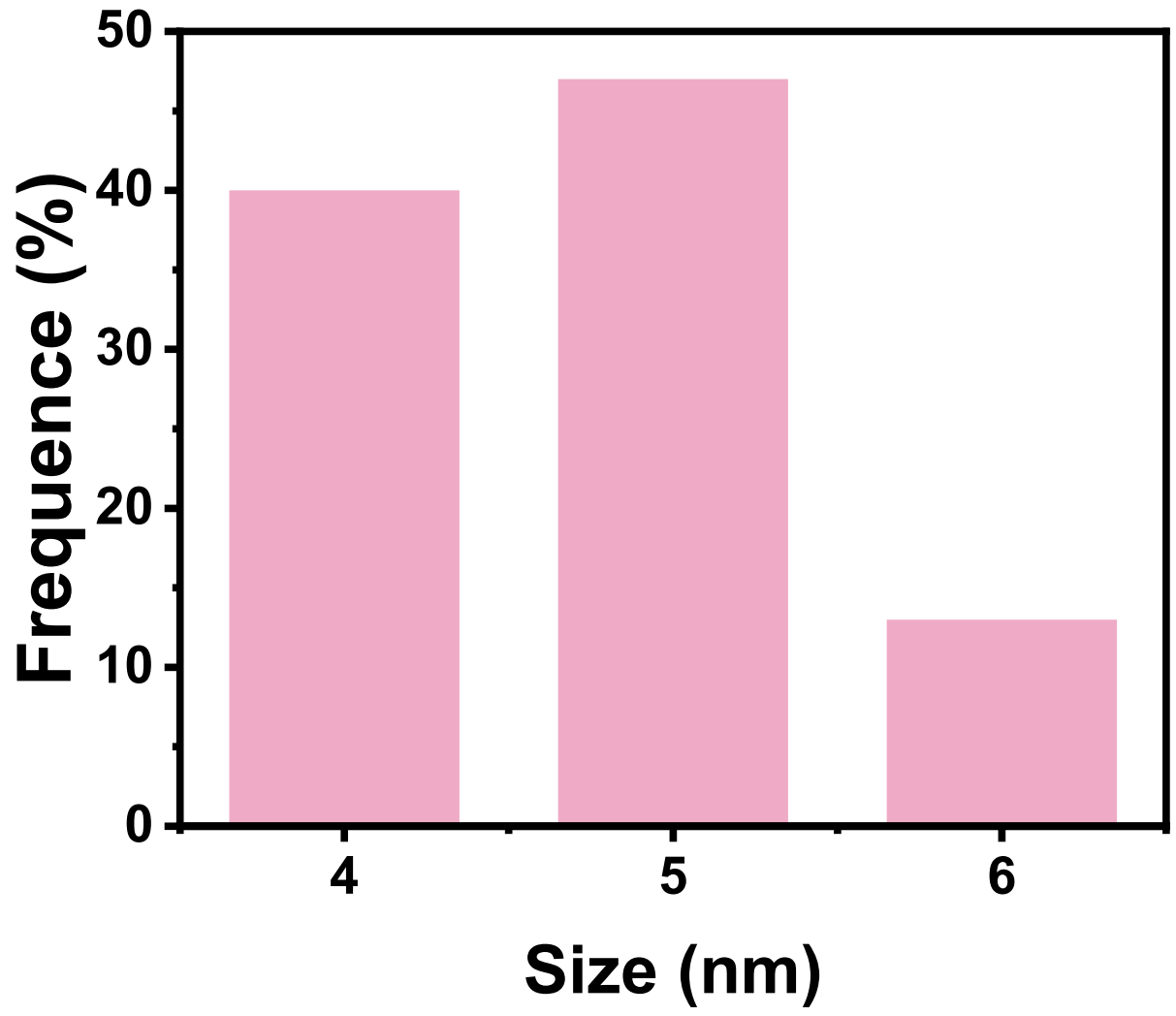

Figure S2. The size distributions of GQDs.

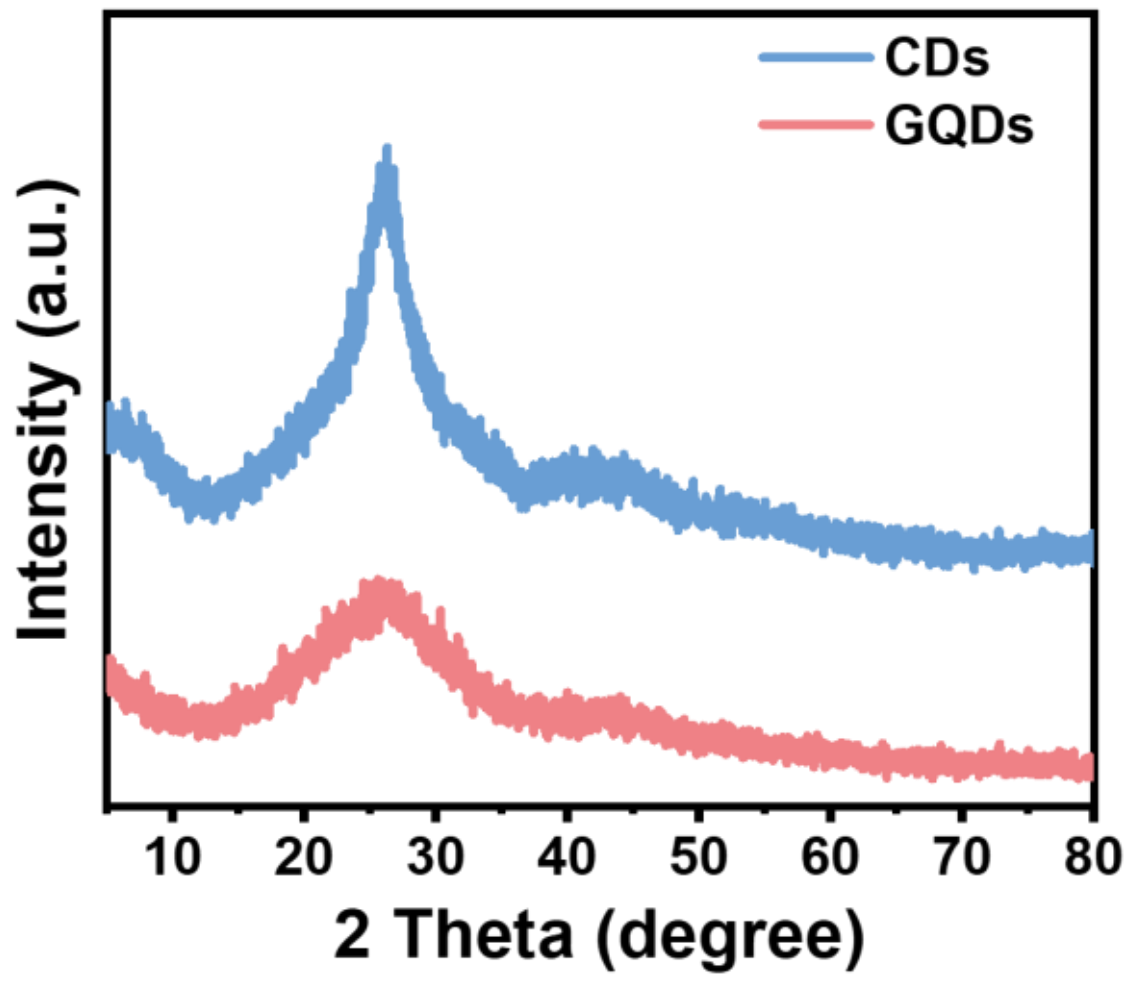

Figure S3. Defect concentration analysis of CDs and GQDs. XRD patterns of CDs and GQDs. Crystalline phase analysis of the powdered materials was performed using X-ray diffraction. Both materials exhibited distinct diffraction peaks at 26° and 42°, corresponding to the (002) and (100) planes of graphene, respectively.

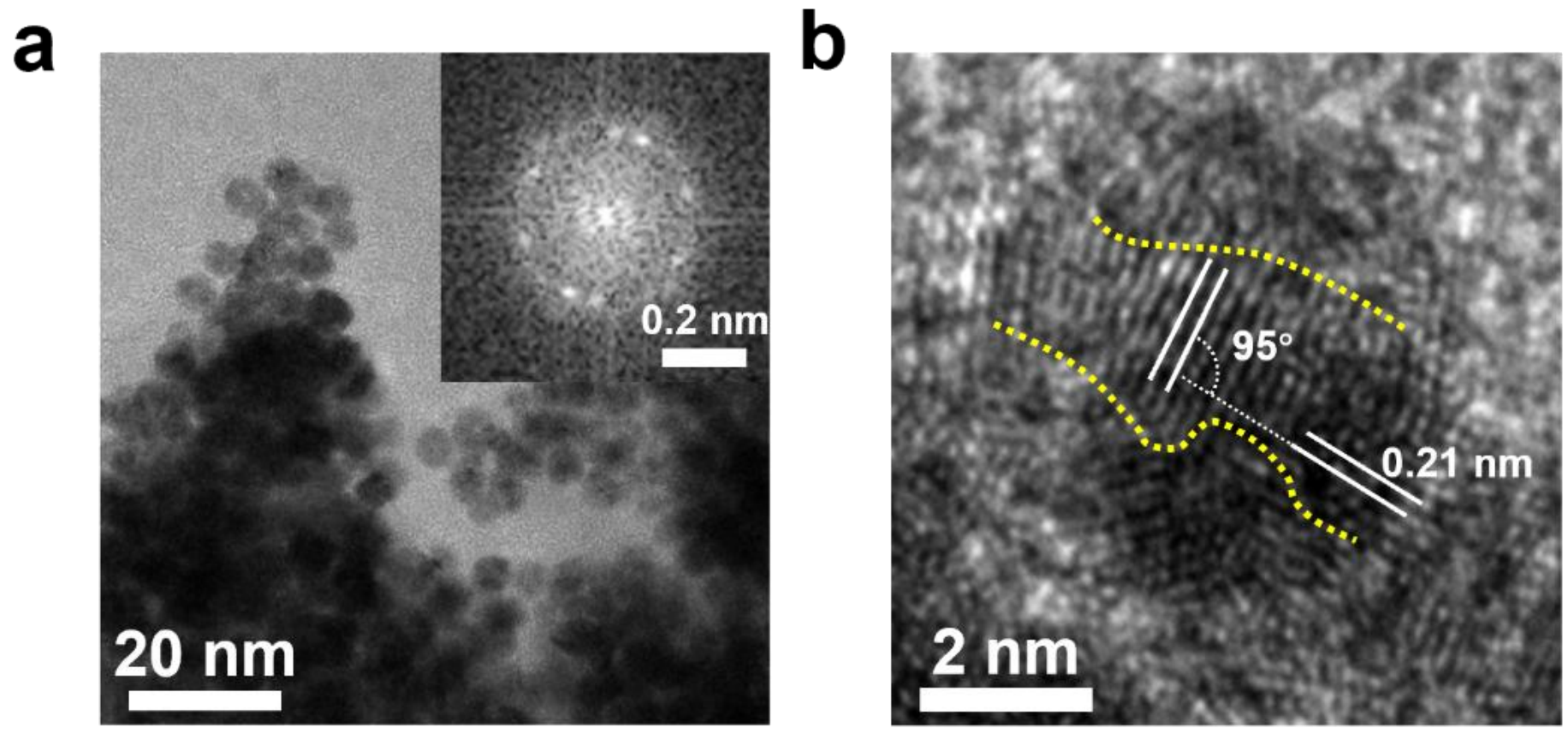

Figure S4. Morphology and structure of CDs. (a) TEM and (b) HRTEM images of CDs, the yellow lines demarcate grain boundaries (inset: FFT of HRTEM image). The FFT pattern of the CDs exhibits faint diffraction spots arranged in a hexagonal lattice. This can be attributed to the disordered structure beyond the grain boundary (marked by the yellow dashed line), while indicating one-dimensional growth of perylene-based molecules in a "side-by-side" configuration.

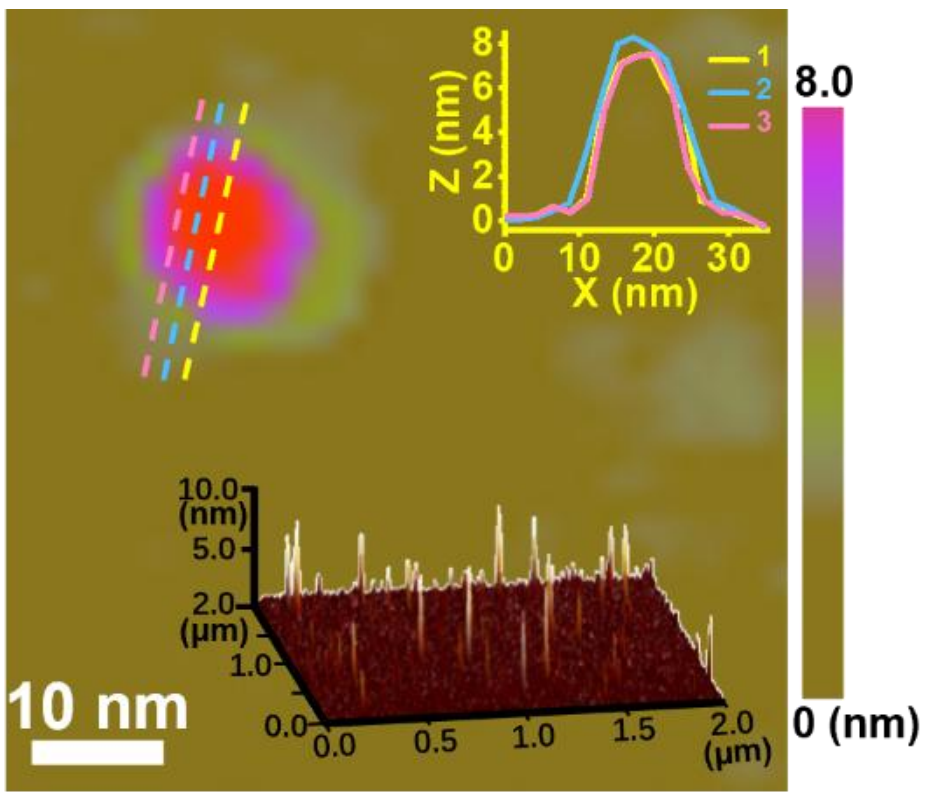

Figure S5. Height analysis of CDs. AFM images of CDs (inset: the height profile along the lines and 3D maps of overall view). Height profiles measured along the dashed lines, showing nearly identical topographic heights between the center and edge of the structures. This uniform height distribution is indicative of a flat-stacked, columnar morphology.

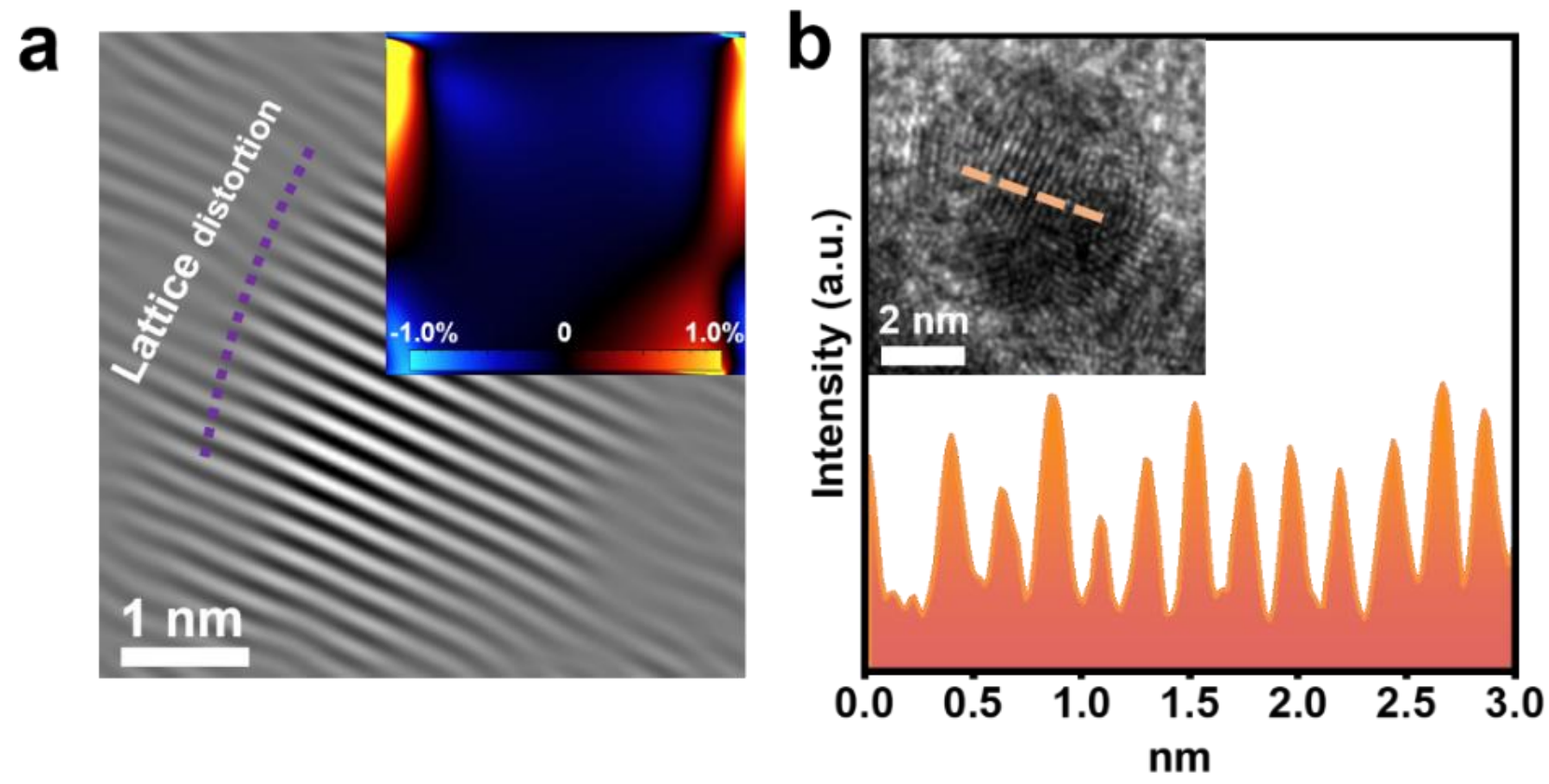

Figure S6. Lattice strain and atomic height profile of CDs. The (a) IFFT patterns and (b) atomic intensity profile along the dashed line in regions of HRTEM image for CDs. In the GPA map, tensile (red) and compressive (blue) strains are displayed. Structural characterization reveals that the core of CDs exhibited clear and ordered Moiré patterns with a regular strain field distribution. The relatively uniform atomic height indicated its flat carbon layer plane.

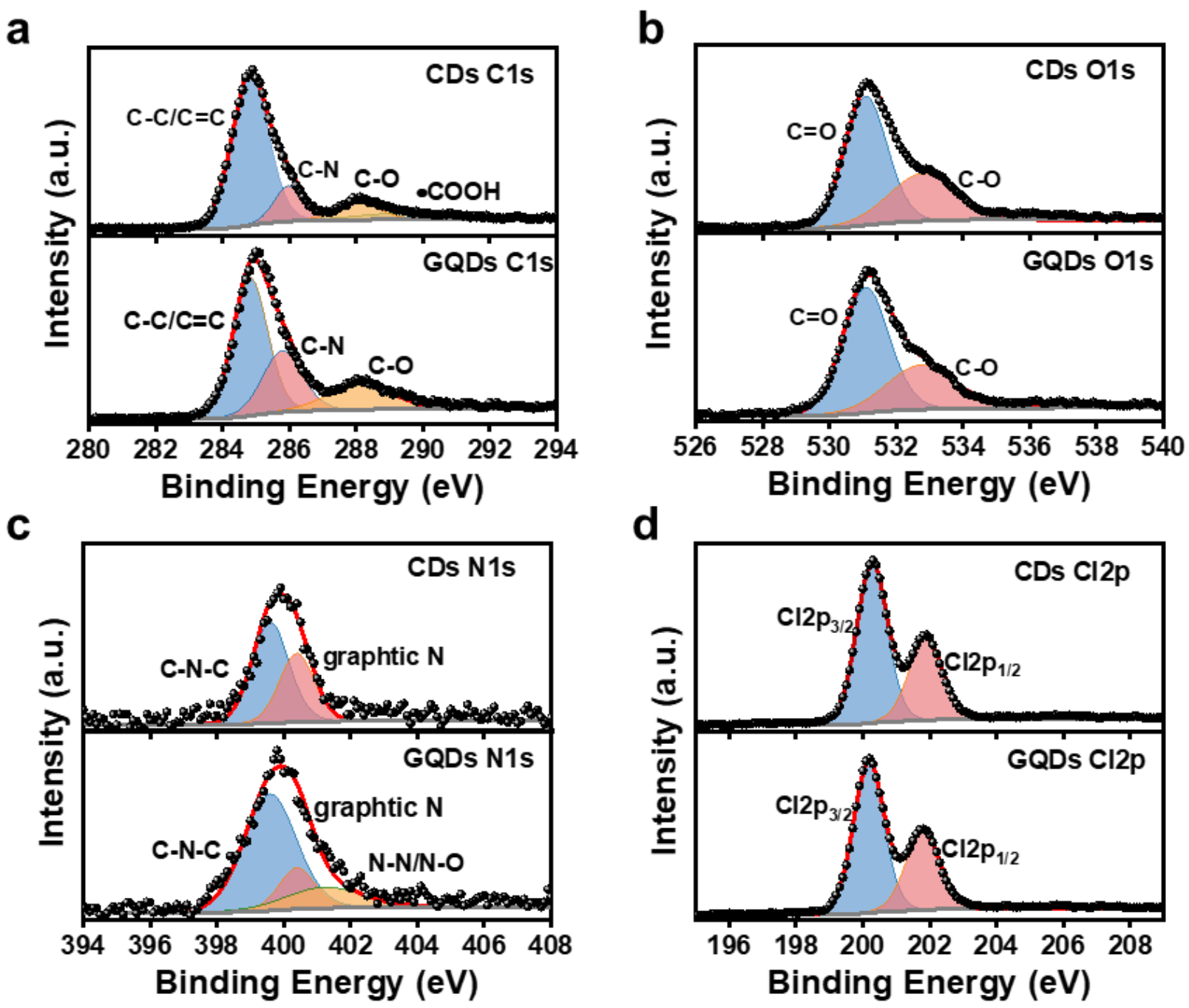

Figure S7. Structural Composition Analysis of CDs and GQDs. High-resolution (a) C1s, (b) O1s, (c) N1s, and (d) Cl2p XPS spectra of the CDs and GQDs. As shown in Figure S3, GQDs exhibited significantly enhanced intensities for C-N (285.9 eV) and C-O (288.1 eV) bonds compared to CDs, while showing no distinct carboxyl peak. This observation clearly demonstrated successful fusion of carboxyl groups from PDI-Cl and effective N/O elemental doping within the graphene lattice (see Table S1).

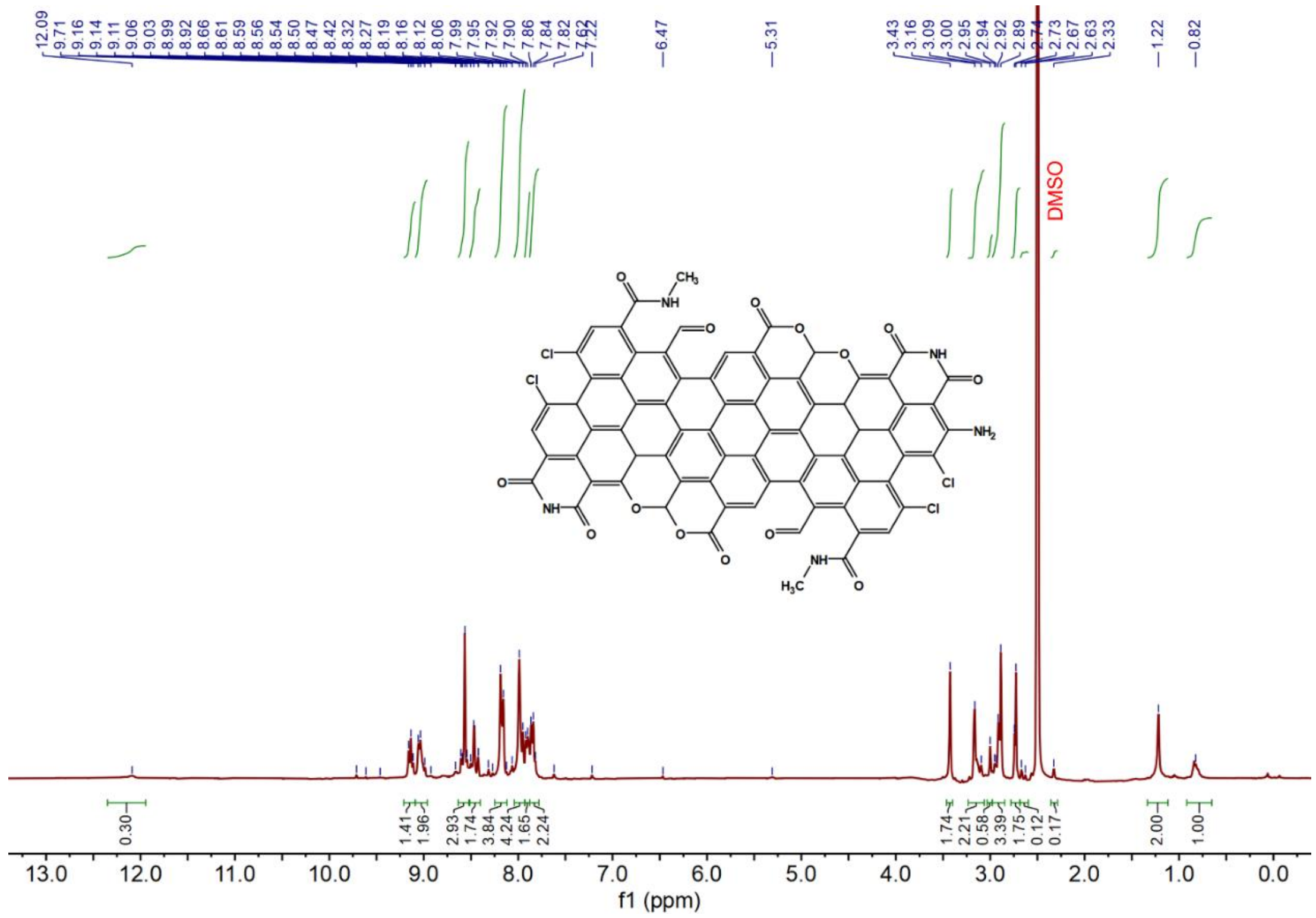

Figure S8. Structural analysis and prediction of CDs. ^{1}H NMR spectra of CDs (5 mg solutions in DMSO-*d6*).

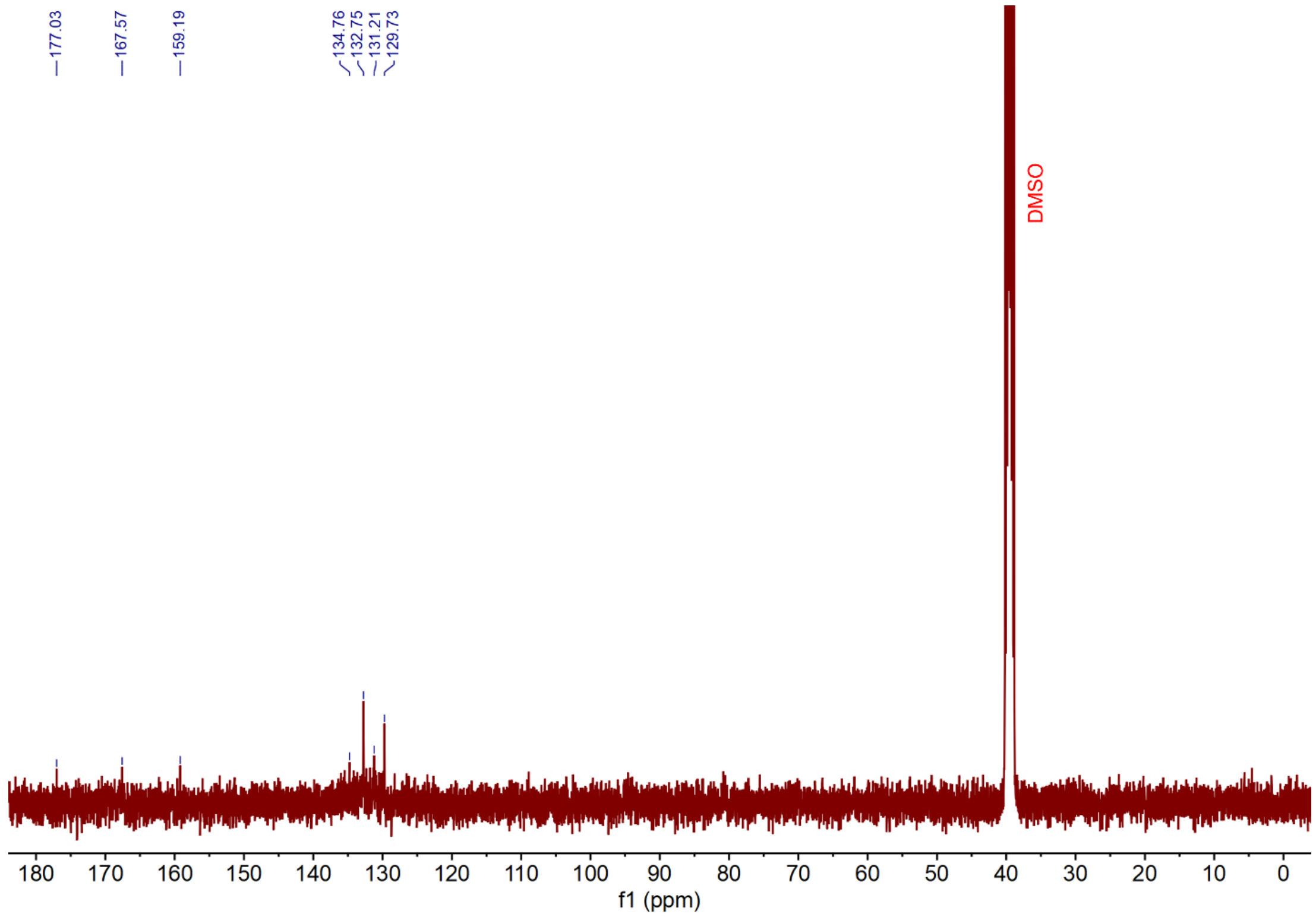

Figure S9. Structural analysis and prediction of CDs. ^{13}C NMR spectra of CDs (20 mg solutions in DMSO-*d6*).

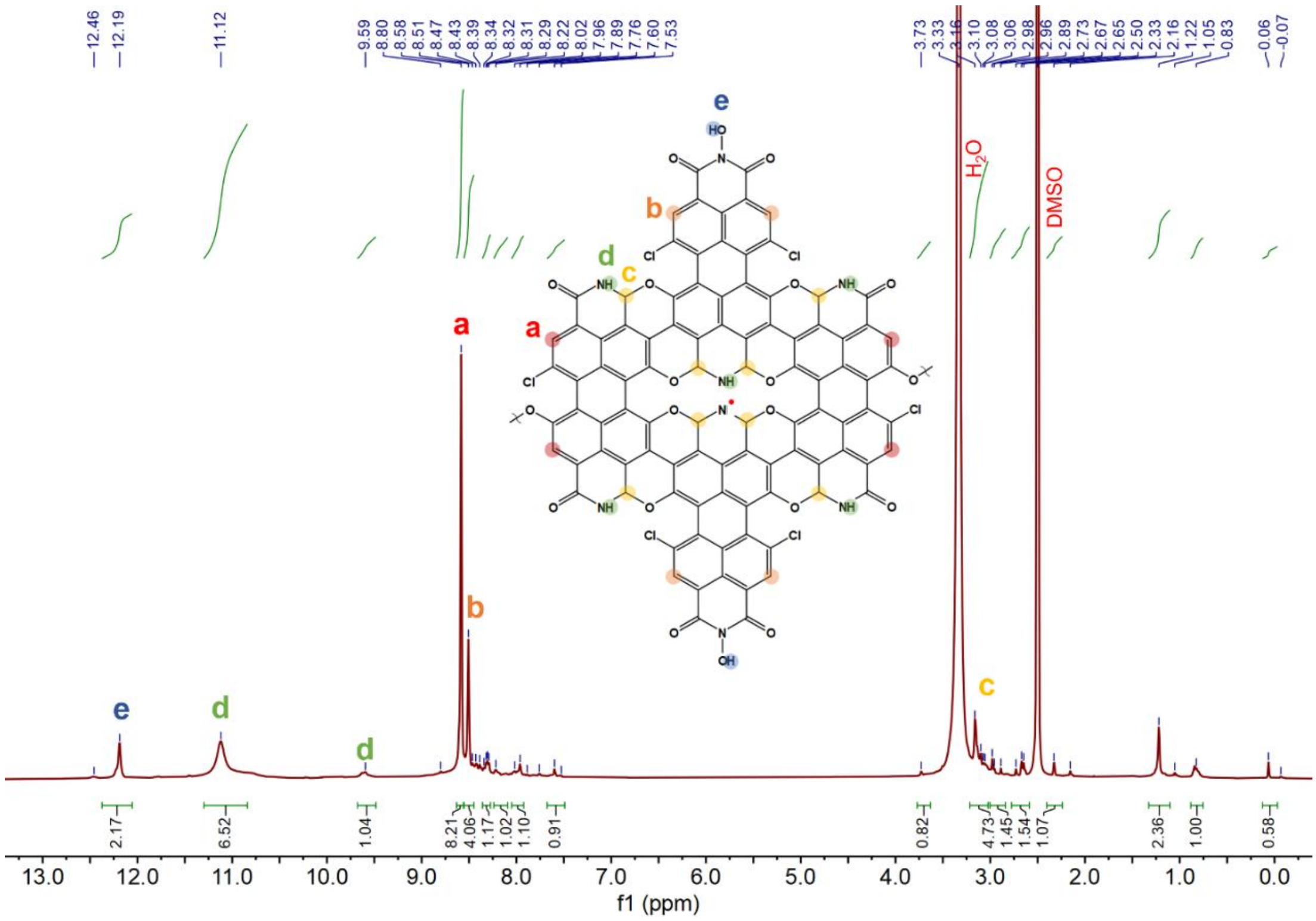

Figure S10. Structural analysis and prediction of GQDs. ¹H NMR spectra of GQDs (5 mg solutions in DMSO-*d6*).

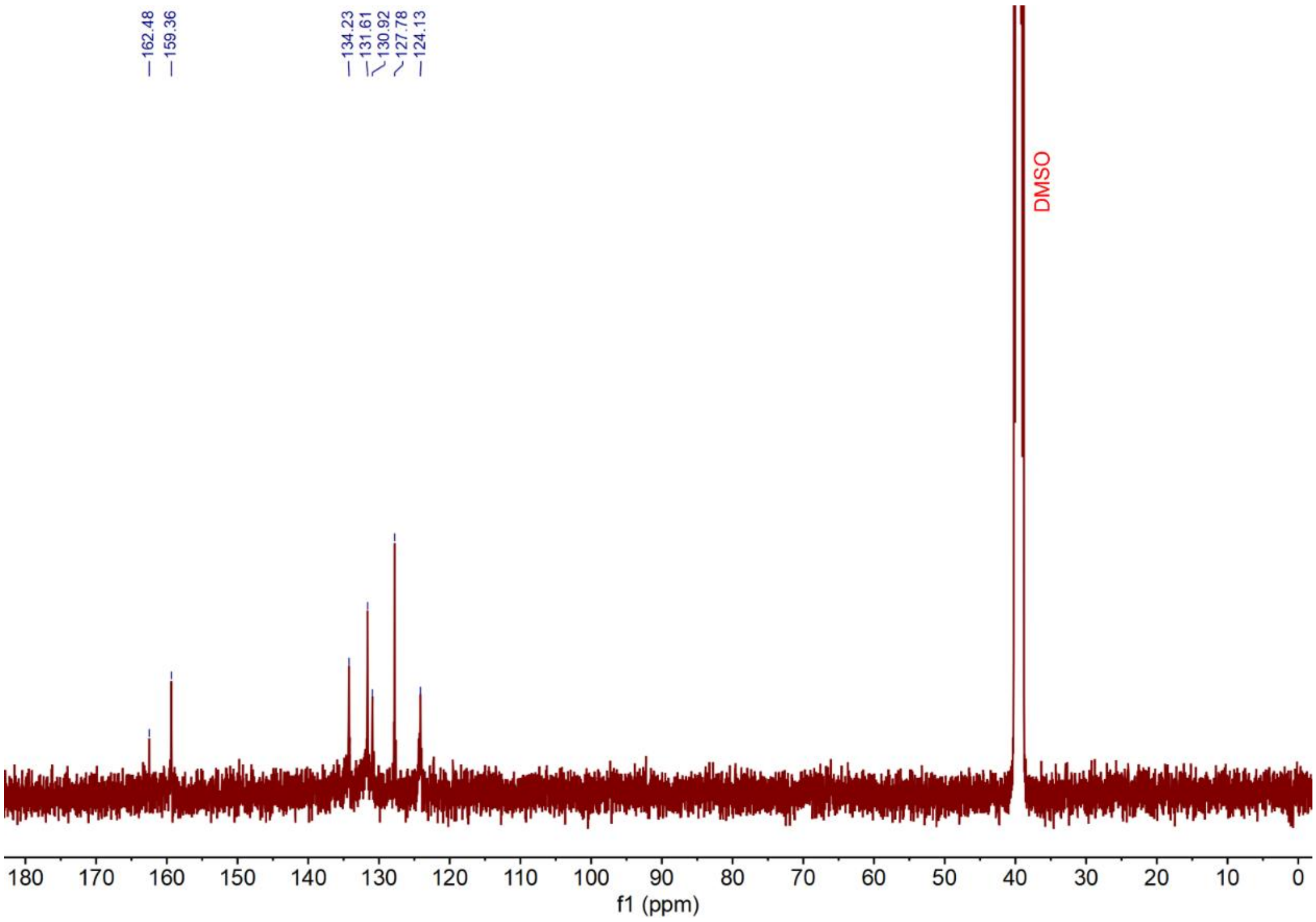

Figure S11. Structural analysis and prediction of GQDs. ^{13}C NMR spectra of GQDs (20 mg solutions in DMSO-*d6*).

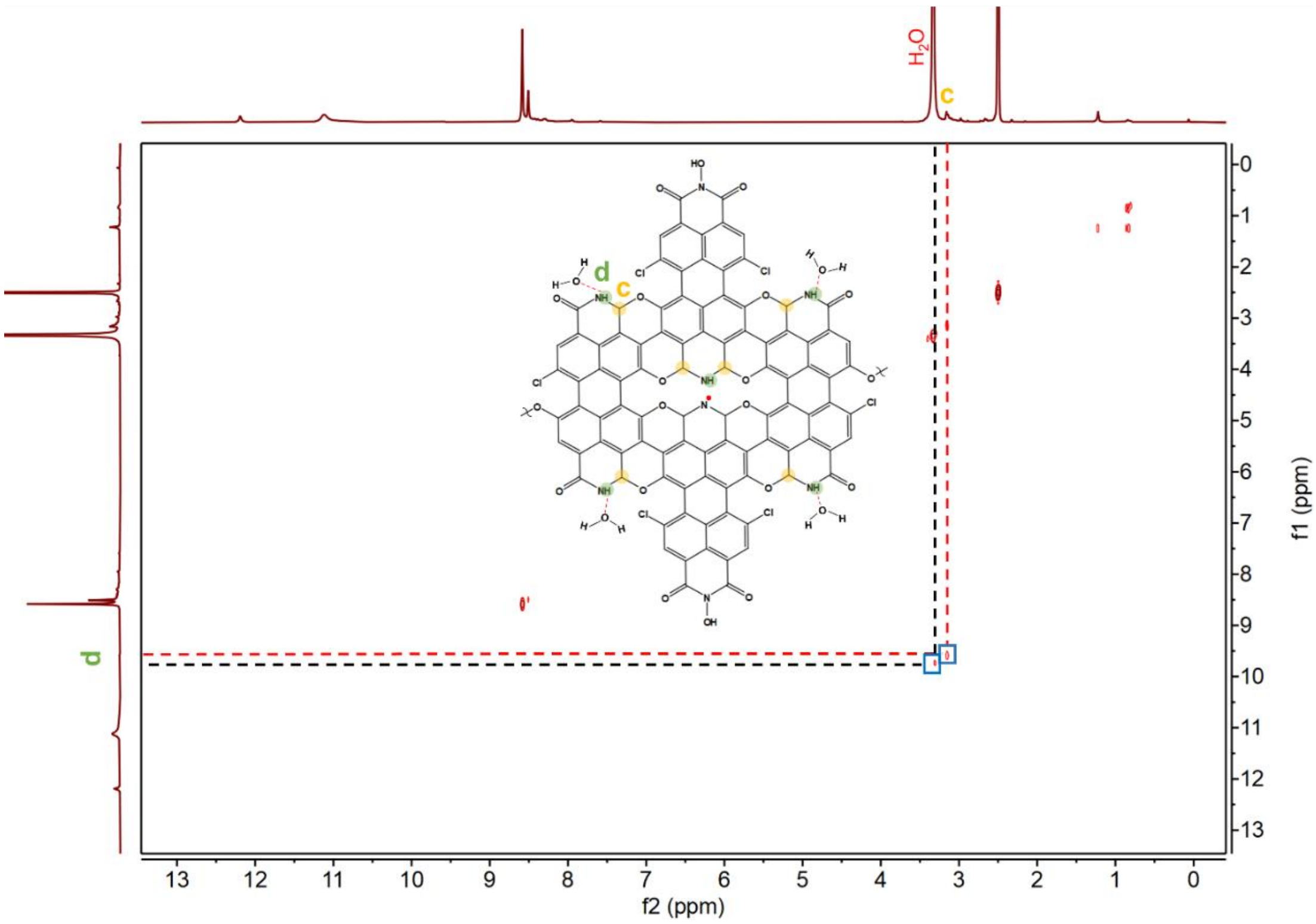

Figure S12. Structural analysis and prediction of GQDs. ^1H-^1H COSY NMR spectroscopic characterization of CQDs. The COSY spectrum was acquired at 400 MHz and 295 K. The important cross-signals are indicated by blue boxes.

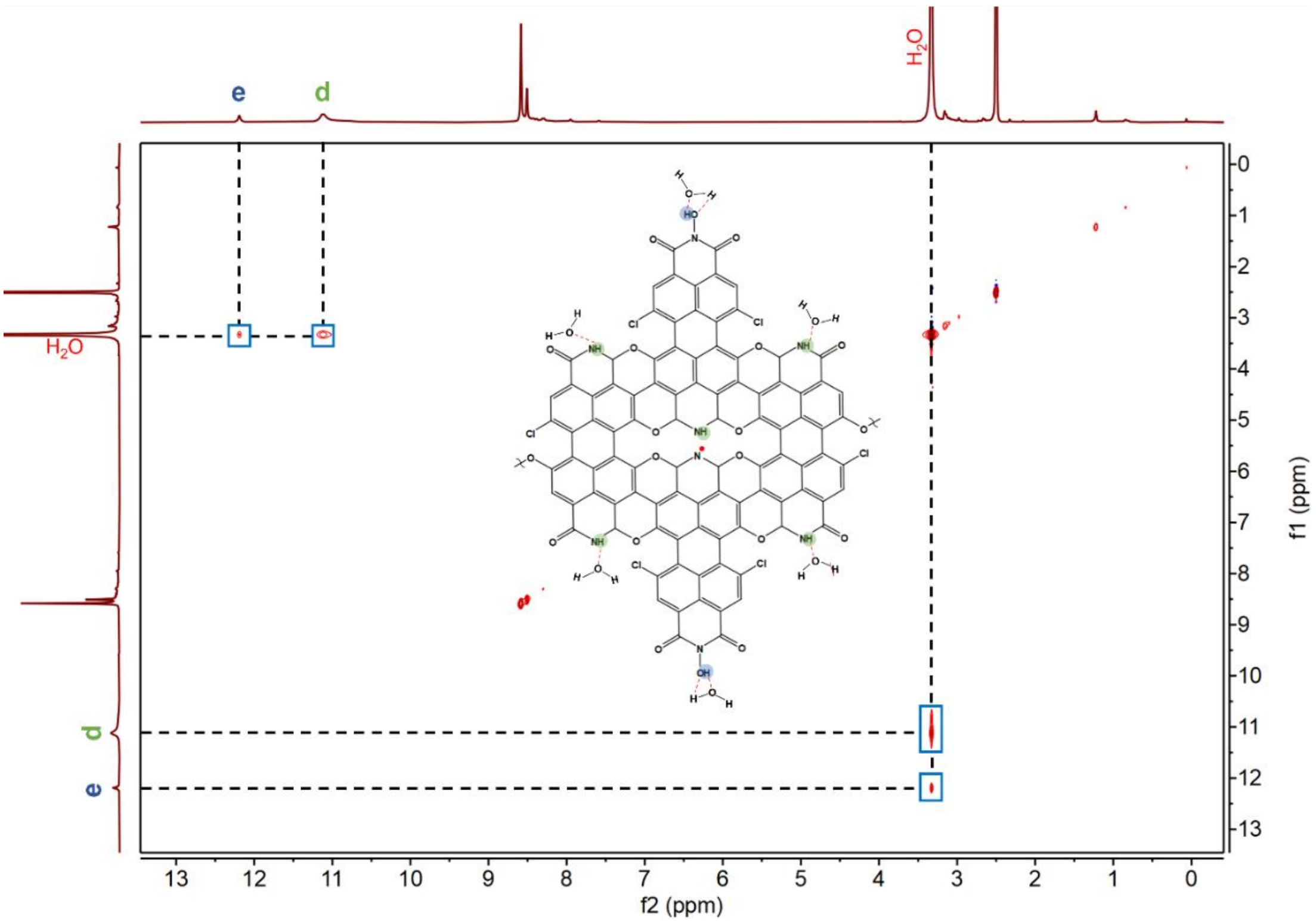

Figure S13. Structural analysis and prediction of GQDs. ^1H-^1H NOESY NMR spectroscopic characterization of CQDs. The NOESY spectrum was acquired at 400 MHz and 295 K. The important cross-signals are indicated by blue boxes.

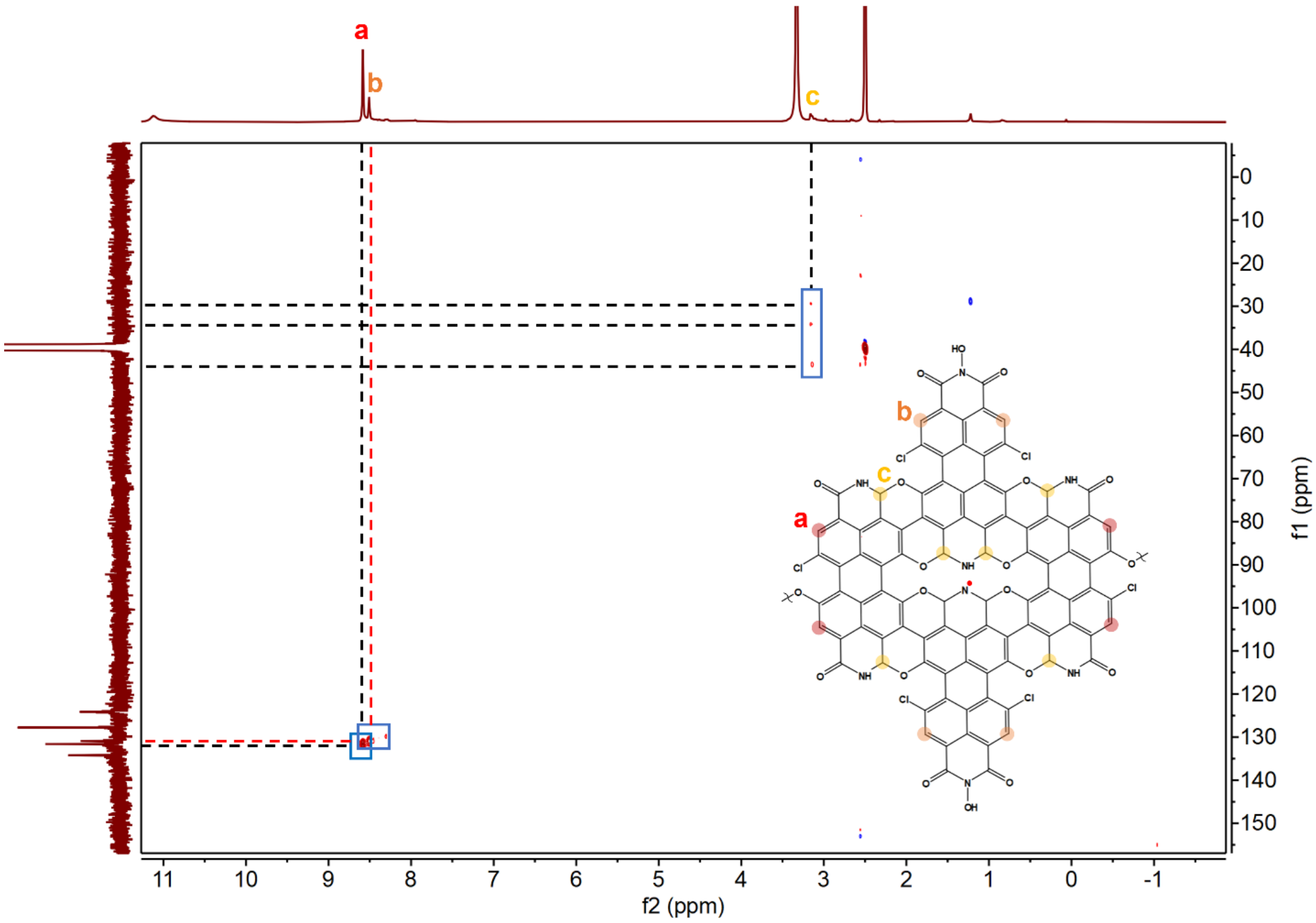

Figure S14. Structural analysis and prediction of GQDs. ^{1}H-^{13}C HSQC NMR spectroscopic characterization of CQDs. The NOESY spectrum was acquired at 400 MHz and 295 K. The important cross-signals are indicated by blue boxes.

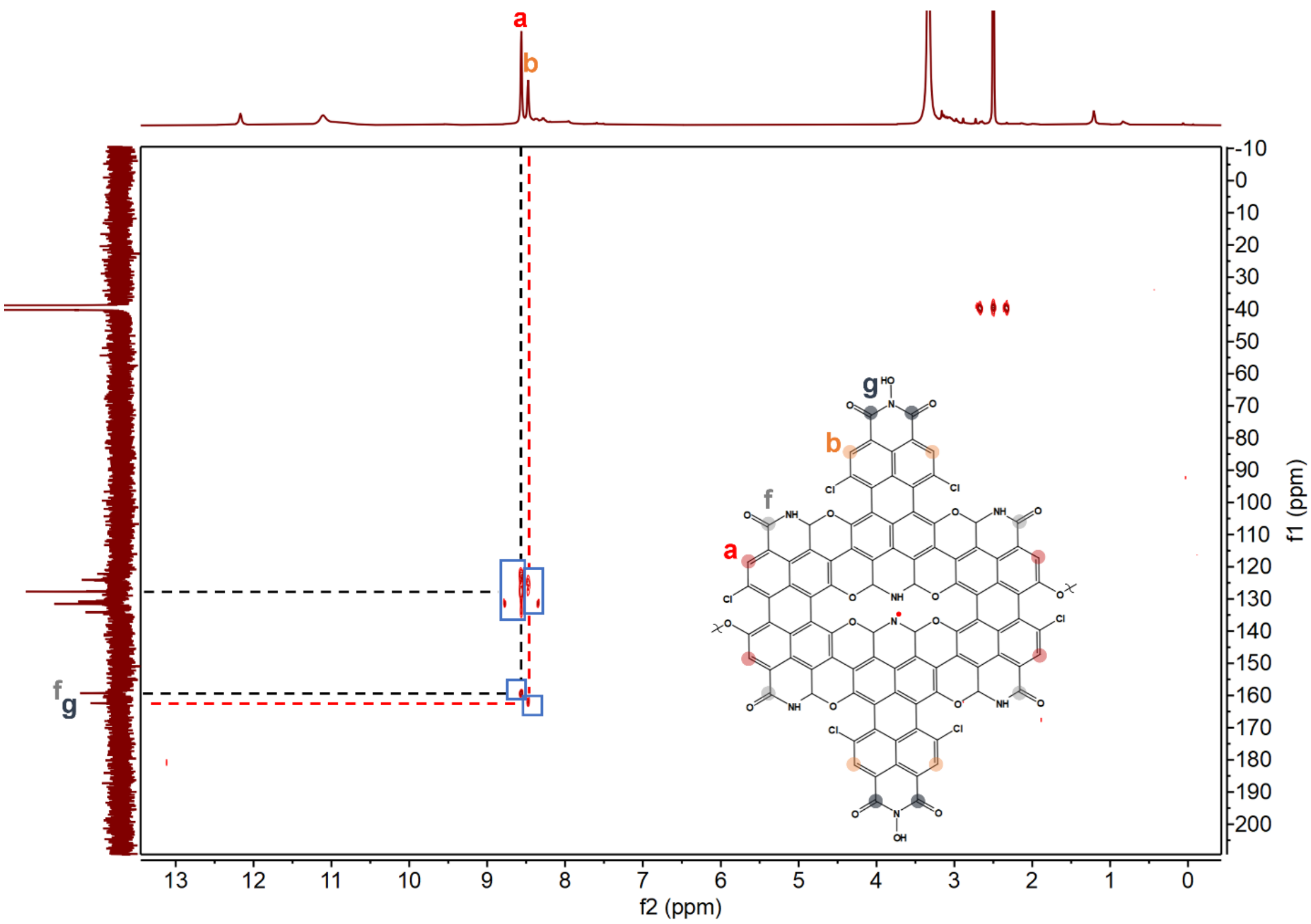

Figure S15. Structural analysis and prediction of GQDs. ^{1}H-^{13}C HMBC NMR spectroscopic characterization of CQDs. The HMBC spectrum was acquired at 400 MHz and 295 K. The important cross-signals are indicated by blue boxes.

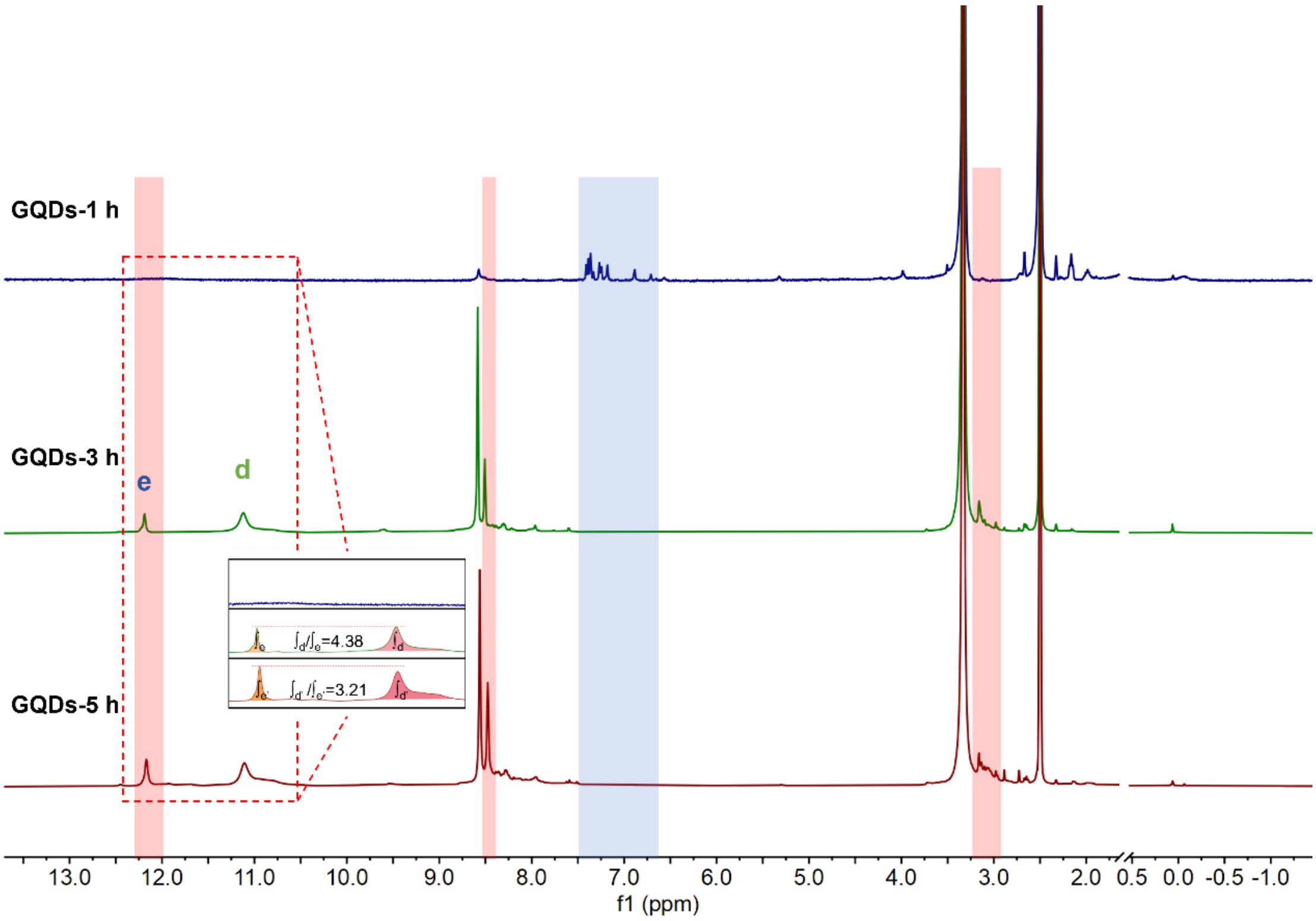

Figure S16. Time-dependent evolution of GQD synthesis. Stacked ^{1}H NMR spectra of GQDs obtained at different reaction times. Prolonged reaction time promotes the fusion of polymerized PDI-Cl molecules and induces the conversion of N-H moieties into internally stabilized N radicals, thereby driving the progressive expansion toward a two-dimensional isotropic configuration.

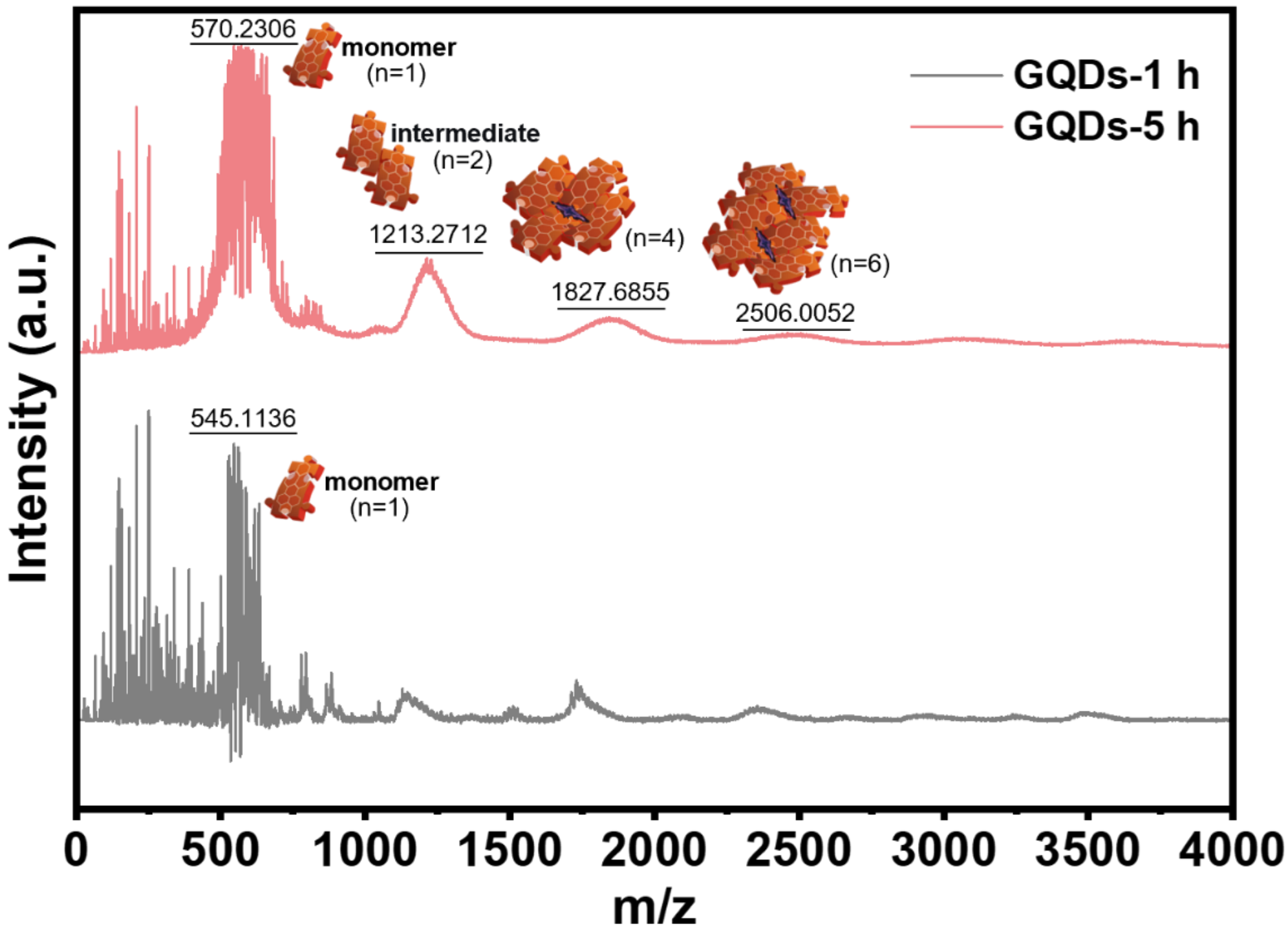

Figure S17. Molecular weight analysis of GQDs. Mass spectrometric characterization of GQDs at different time points during the reaction. At 1 h, the GQDs likewise existed primarily as monomers. After 5 h, however, the mass spectrum revealed a periodic progression of m/z peaks. Notably, the observed peaks align closely with the molecular weights of the predicted structures. This indicates that hydroxylamine hydrochloride promotes the fusion of perylene monomers into an intermediate state (e.g., n=2), thereby driving the two-dimensional planar fusion and extension of the GQDs.

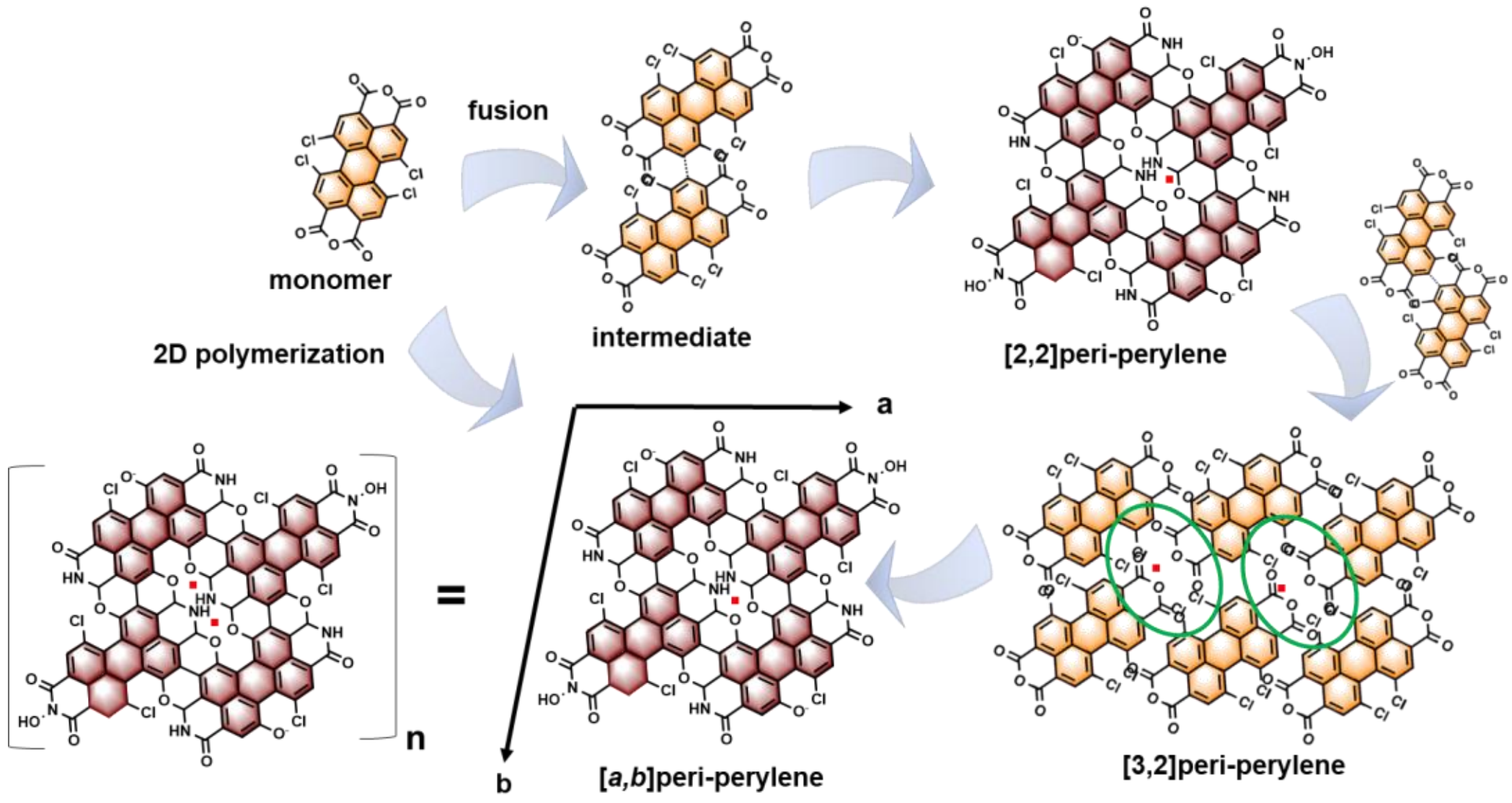

Figure S18. The scheme of 2D polymerization process of perylene-based monomer.

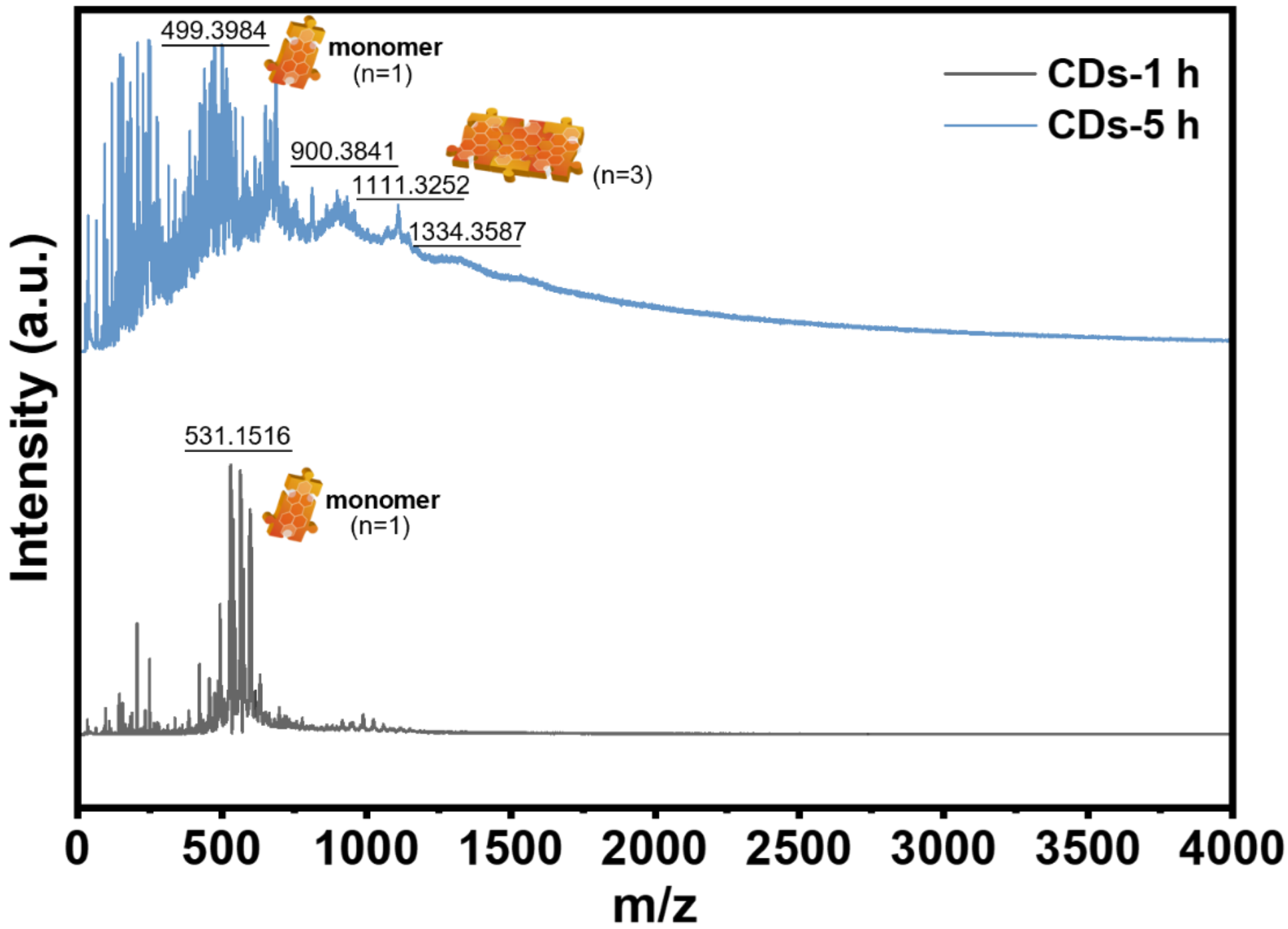

Figure S19. Molecular weight analysis of CDs. Mass spectrometric characterization of CDs at different time points during the reaction. At a reaction time of 1 h, the CDs existed predominantly as monomers. After 5 h, the mass spectrum displayed a broad m/z peak, originating from the formation of disordered peripheral structures during CD fusion.

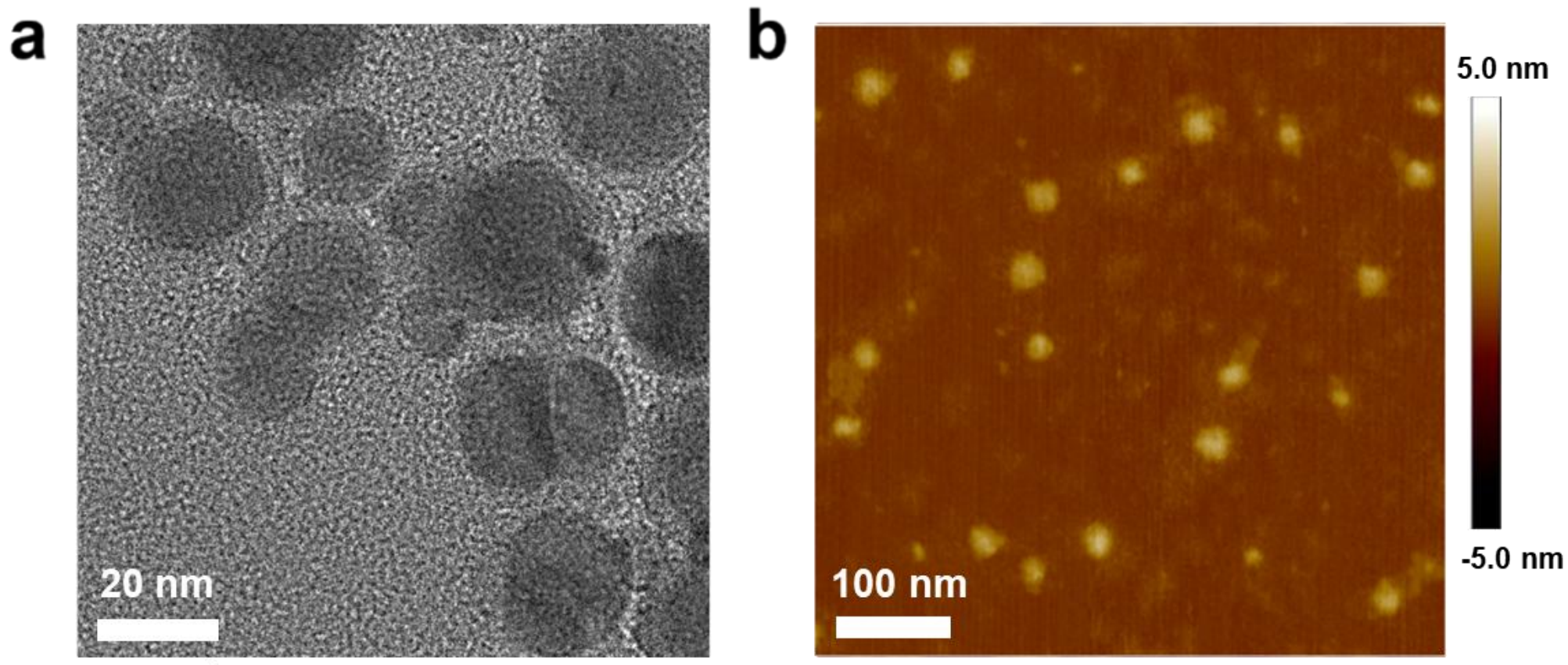

Figure S20. Morphological characterization of GQDs-10 h. (a) TEM image of the synthesized GQDs. (b) AFM topography analysis. The increase in lateral dimension with reaction time, without a change in the number of stacked layers, indicates a scalable two-dimensional sheet-like morphology.

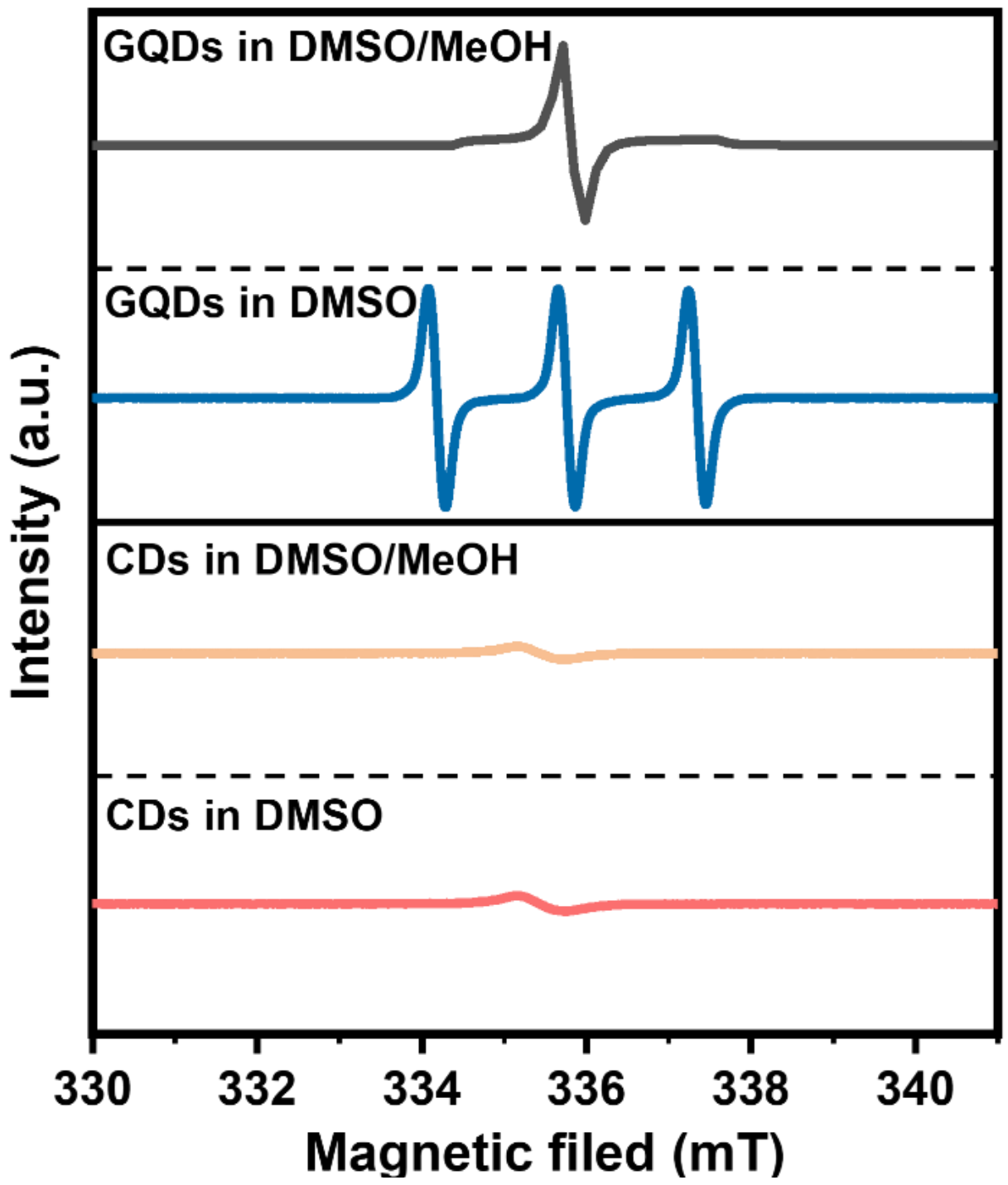

Figure S21. EPR spectra of CDs and GQDs dispersed in DMSO or DMSO/methanol (DMSO/MeOH) mixtures. Notably, GQDs dispersed in DMSO exhibit a well-resolved hyperfine triplet characteristic of N-centered radicals. Upon the addition of 5% v/v methanol, this triplet collapses into a single line, indicative of spin-exchange averaging. In contrast, CDs consistently exhibit a negligible single-line signal predominantly associated with carbon-centered radicals.

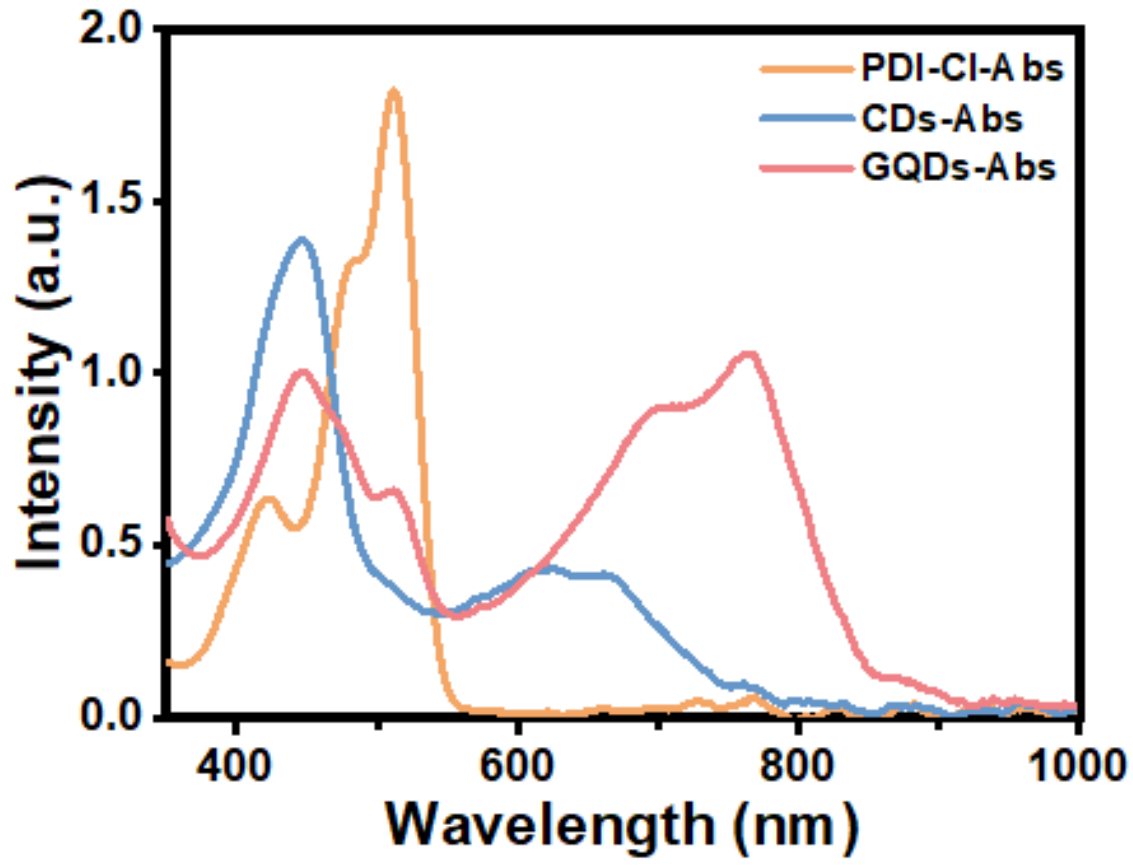

Figure S22. The absorption spectra of PDI-Cl, CDs, and GQDs with 30 μg/mL in DMSO.

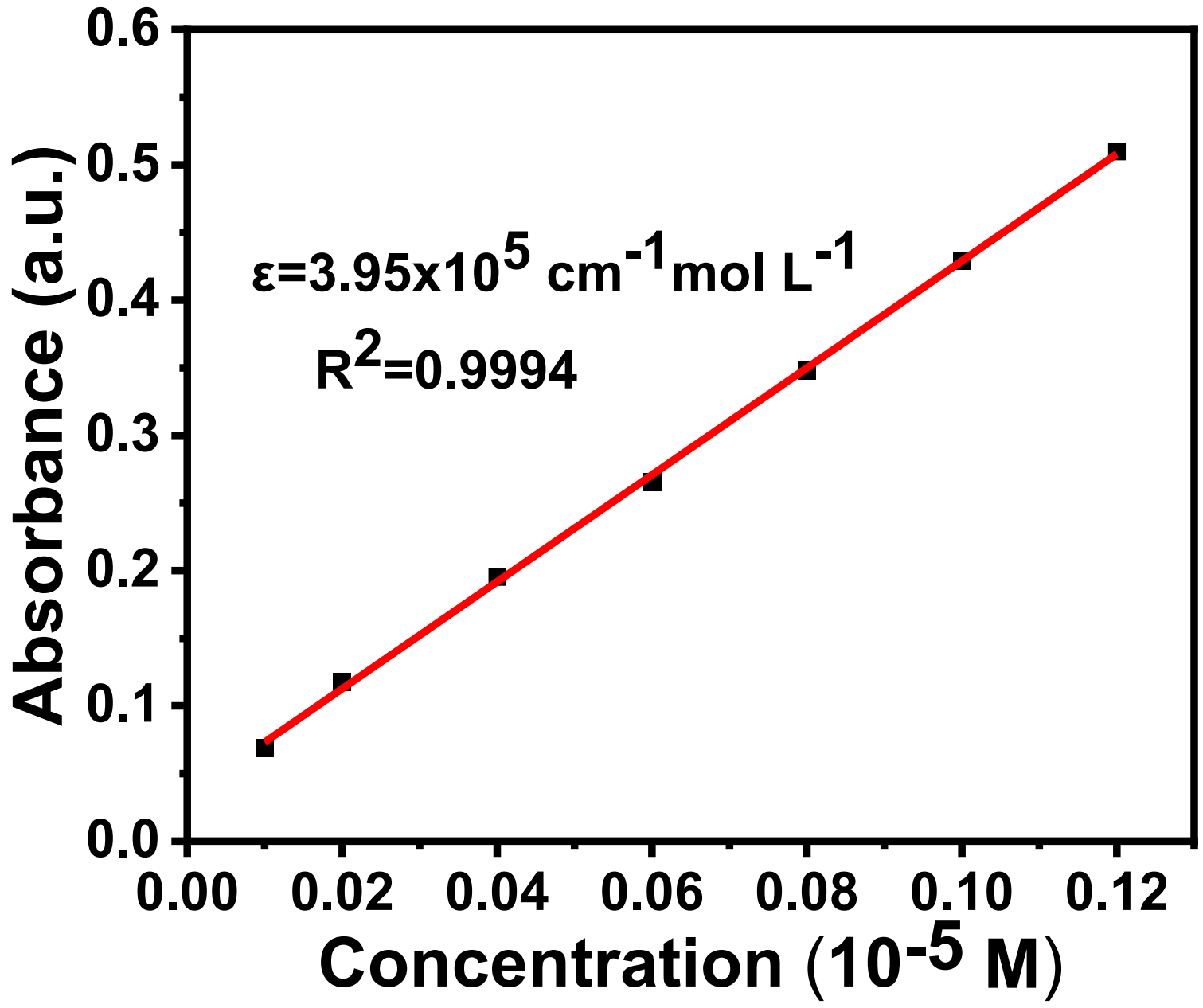

Figure S23. The plot of absorption density at 808 nm versus concentration.

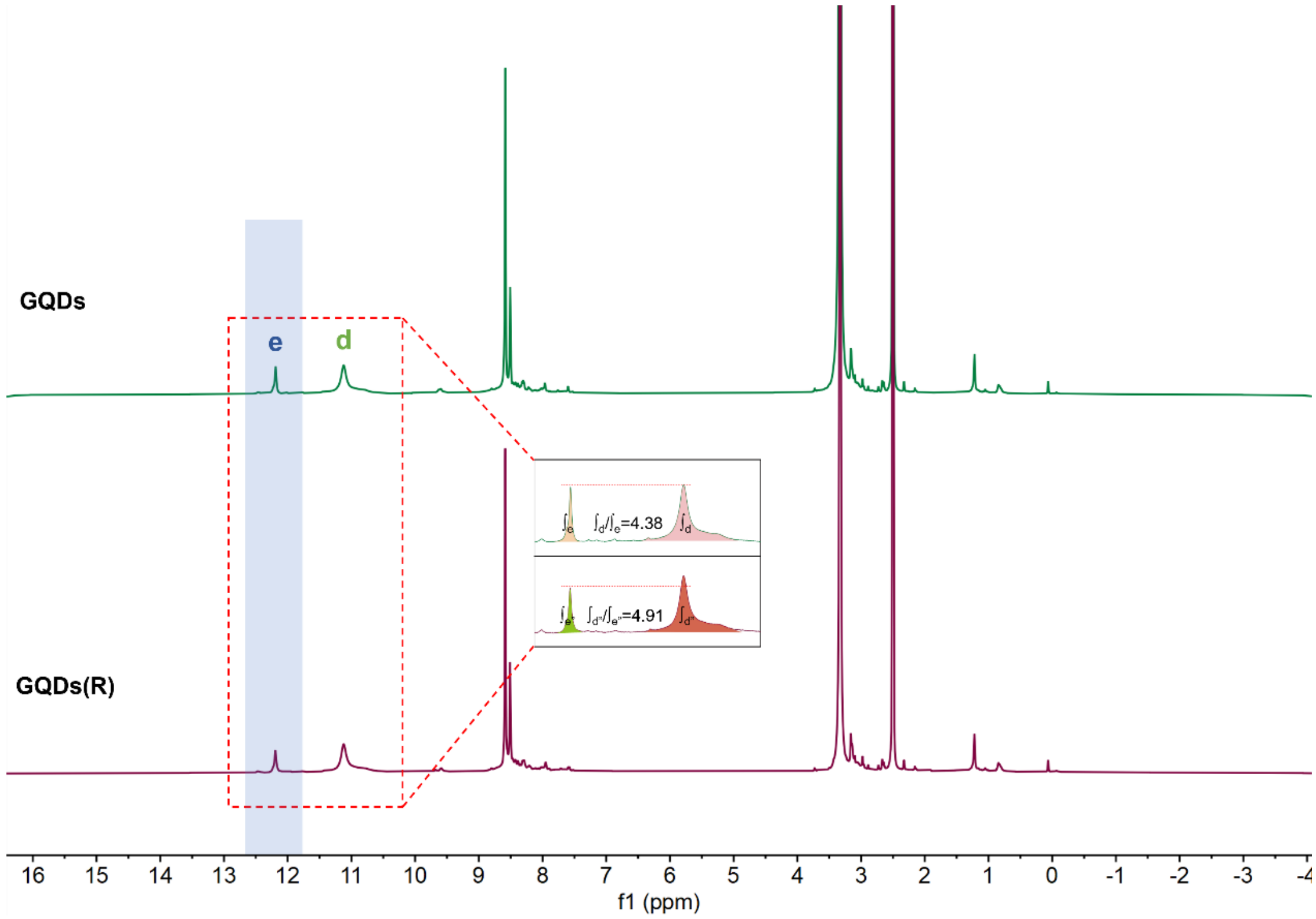

Figure S24. Radical site probing in GQDs. ^{1}H NMR spectra of pristine GQDs and radical-quenched GQDs(R). The increase in integration of the **d** peak upon partial radical quenching, with other signals remaining constant, identifies this site as the location of the radicals, consistent with an increase in hydrogen atom population.

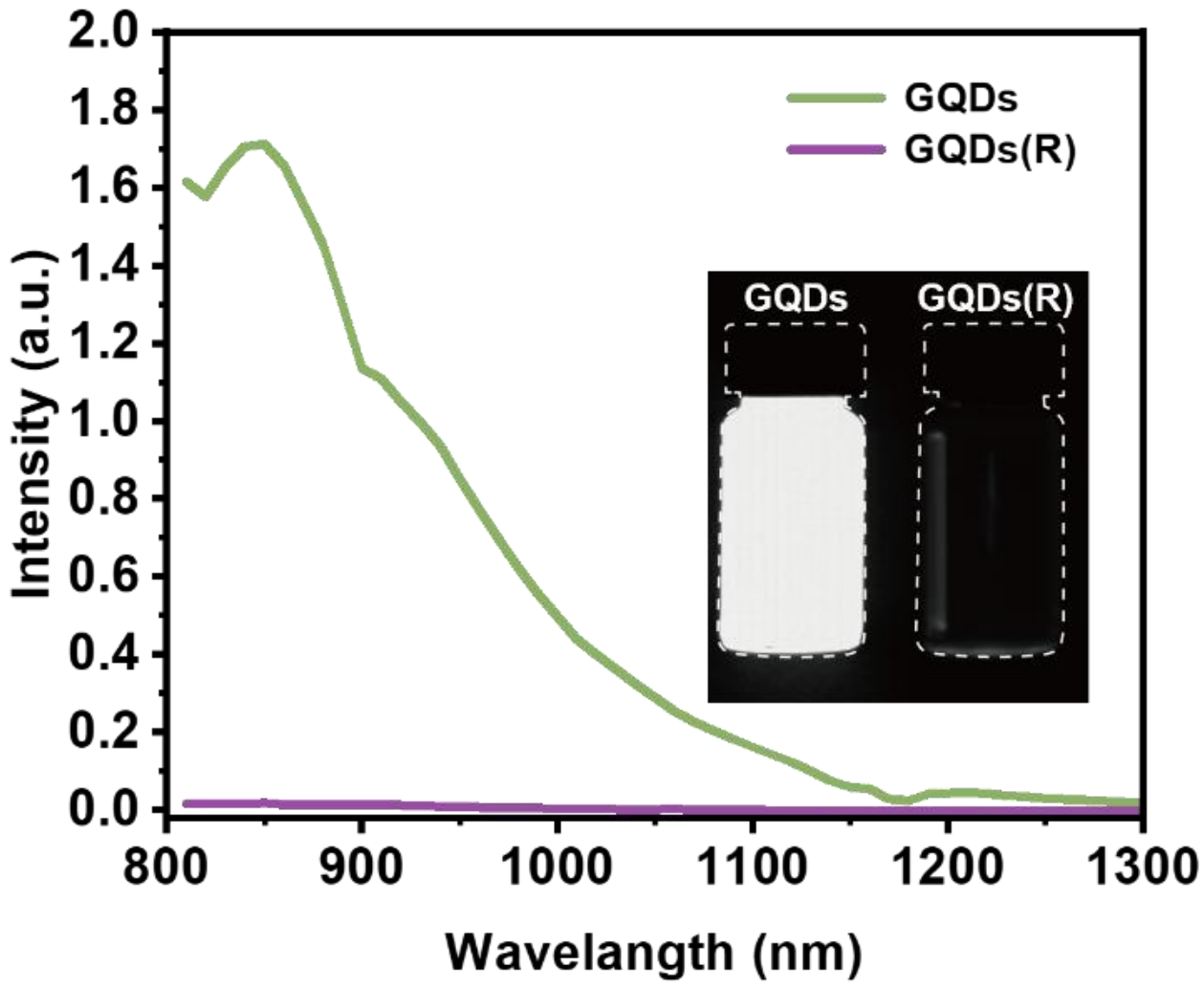

Figure S25. Linking radical species to NIR fluorescence. Photoluminescence spectra of pristine and radical-quenched GQDs(R) under 808 nm excitation (inset: corresponding NIR fluorescence images; Excitation: 808 nm, long-pass filter: 900 nm). The quenching of radicals drastically diminishes the NIR fluorescence while leaving the primary chemical structure intact, unmasks their critical role in generating the NIR emission.

3. Fluorescence characterization

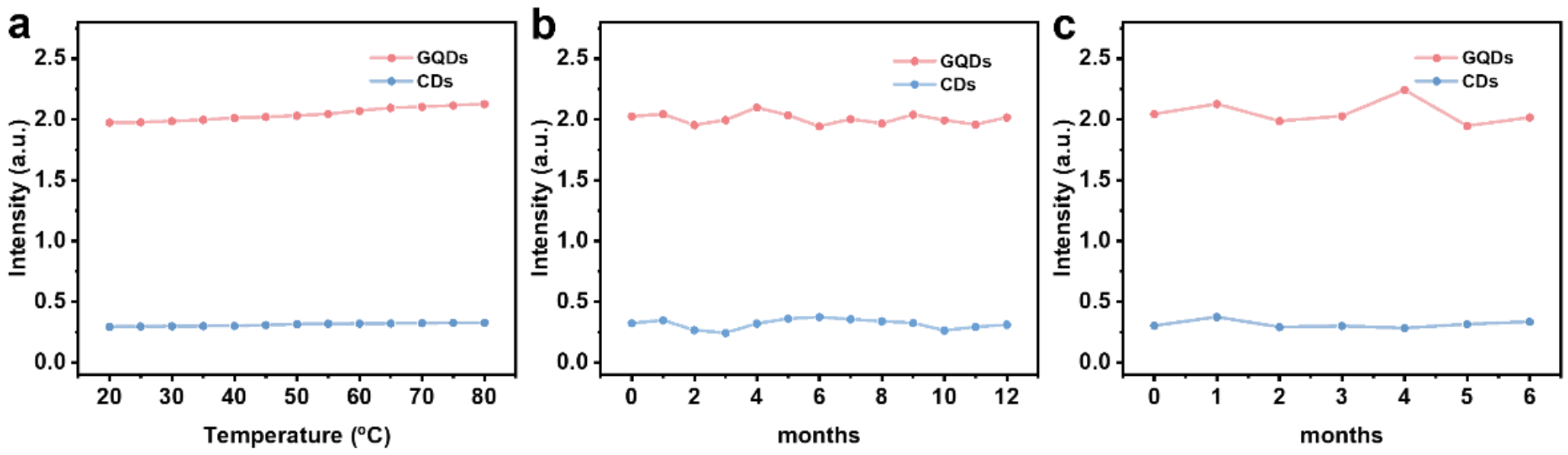

Figure S26. Photostability of radical-derived fluorescence. (a) The near-infrared fluorescence intensity of CDs and GQDs under 808 nm excitation in DMSO at different temperatures. (b) Temporal evolution of fluorescence intensity during storage in a sealed container at room temperature. (c) Fluorescence intensity variation upon exposure to ambient storage at 60% relative humidity. The experimental data demonstrate that both CDs and GQDs exhibit excellent long-term fluorescence stability under specific thermal and humidity conditions.

4. Quantitative analysis of radicals

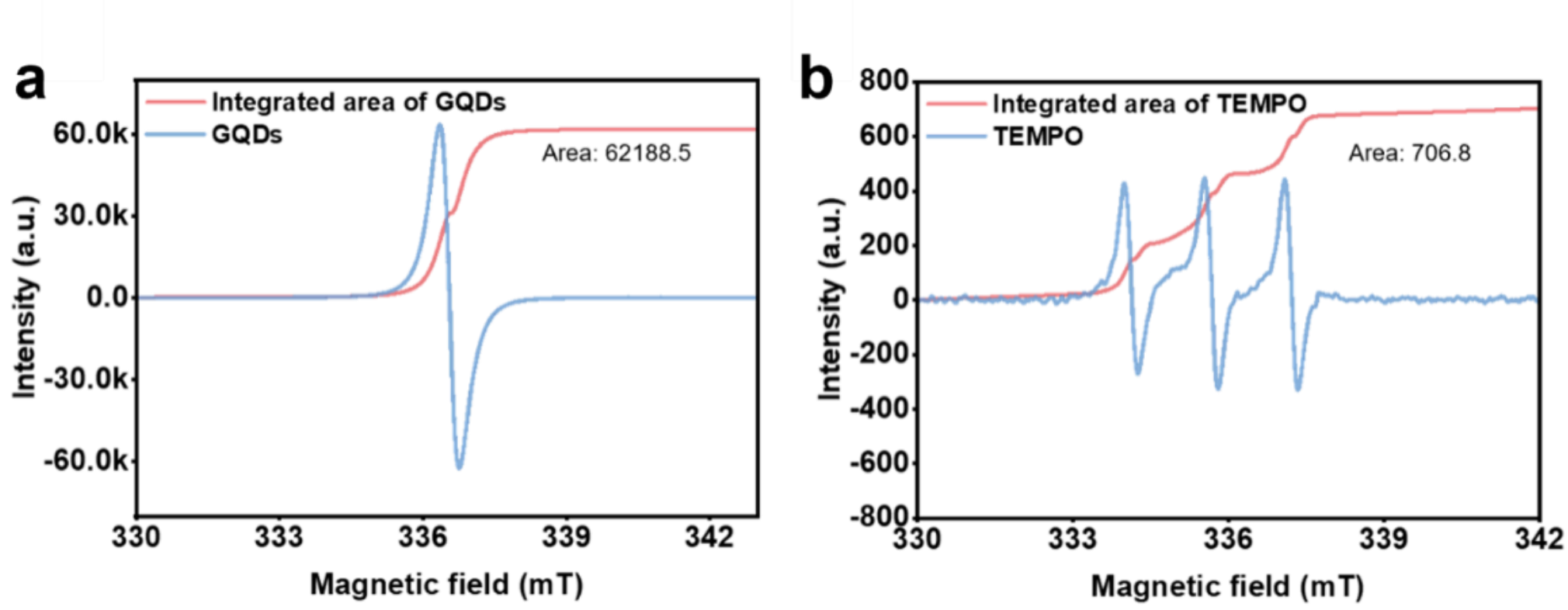

Figure S27. Quantitative detection of radicals in GQDs. The EPR spectra of (a) GQDs and (b) TEMPO were measured under dark conditions at room temperature. Radical signals from TEMPO and GQDs were integrated, and the radical concentration in GQDs was quantitatively analyzed using the qEPR methodology described in Eq. (S4) and Eq. (S5).

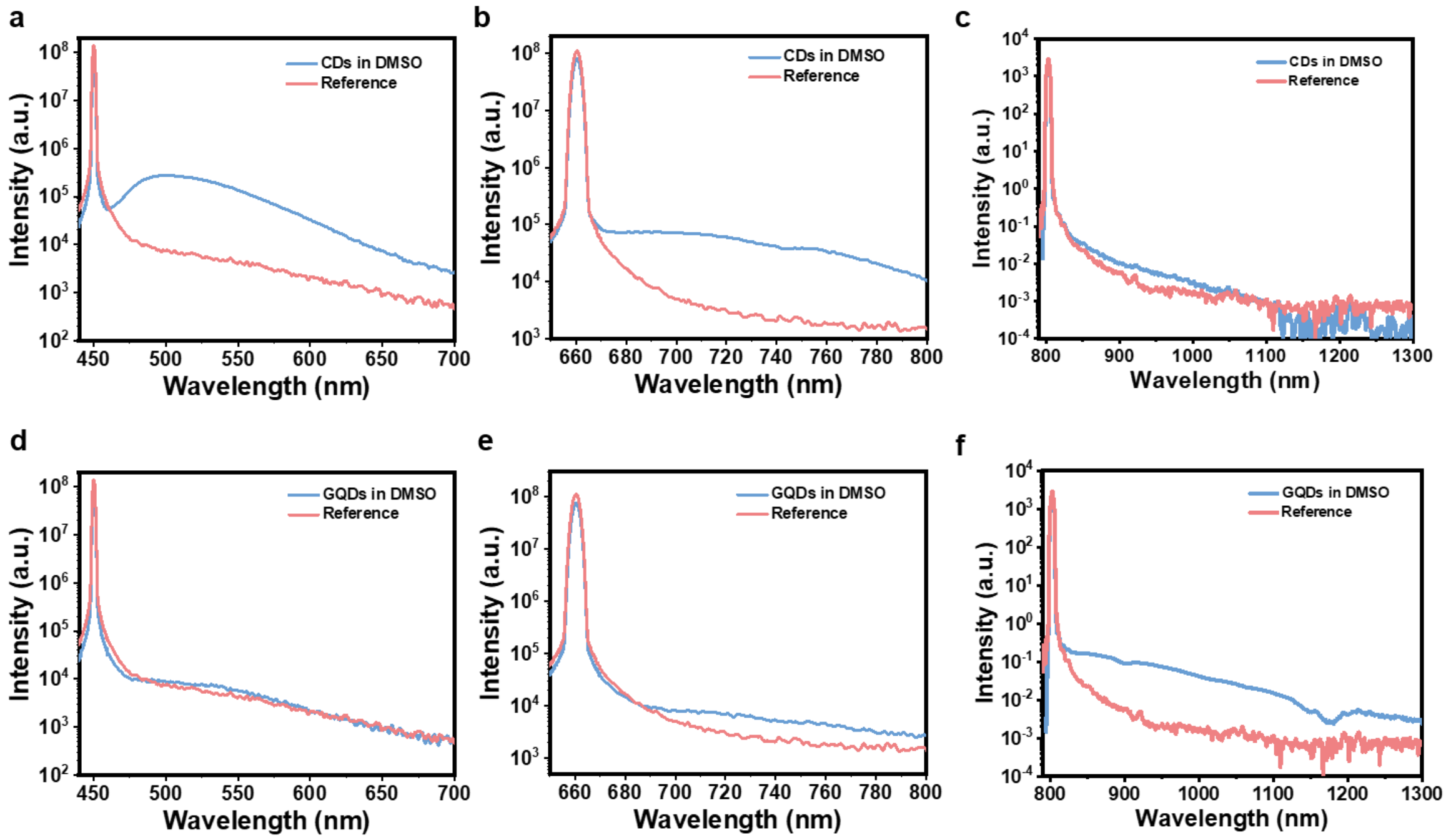

Figure S28. PL intensity analysis of CDs and GQDs. Absolute photoluminescence quantum yields of (a), (b), (c) CDs and (d), (e), (f) GQDs in DMSO under 450 nm, 660 nm and 808 nm laser irradiation, respectively.

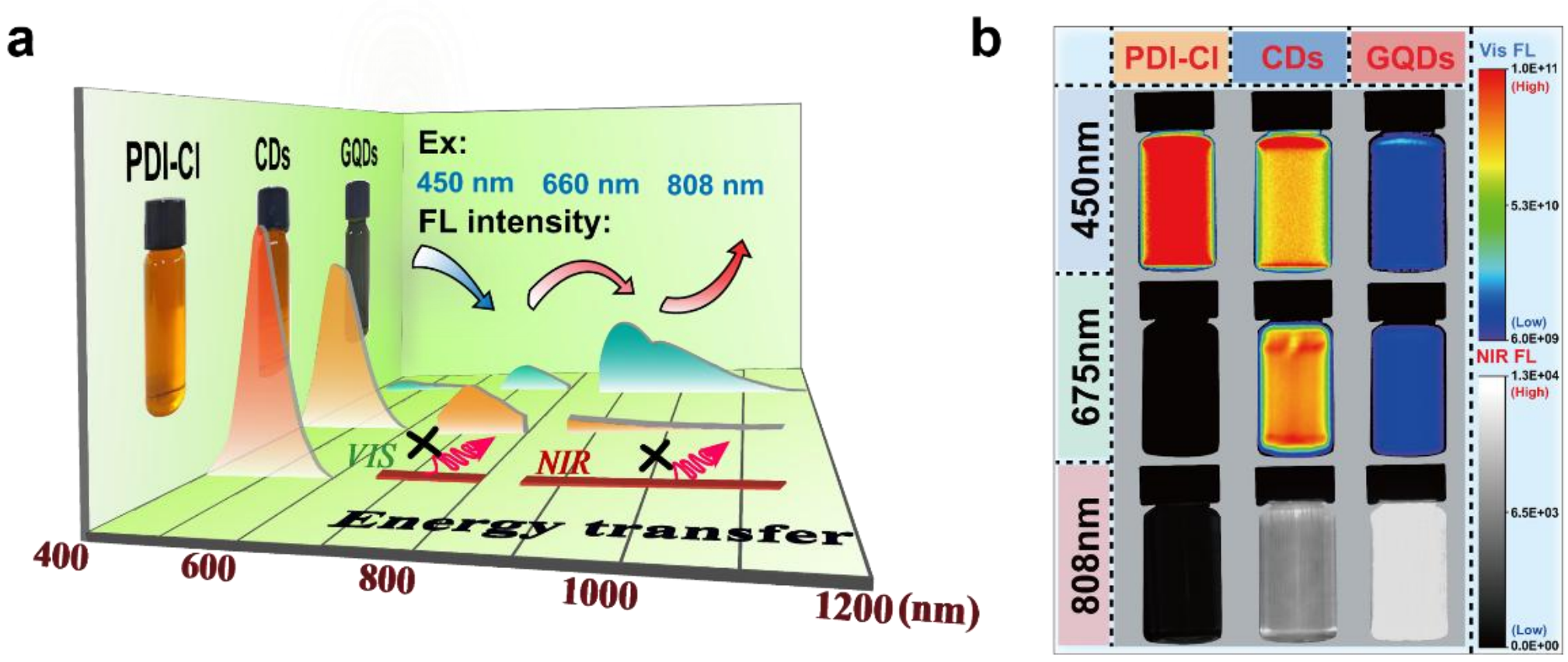

Figure S29. Multi-spectral fluorescence characterization. (a) Fluorescence spectra and (b) *ex vivo* images of PDI-Cl, CDs, and GQDs across different spectral windows. Samples were excited using lasers at 450 nm, 660/675 nm, and 808 nm. PDI-Cl and CDs exhibited strong emission in the visible range. In contrast, GQDs exhibited predominant emission in the NIR range.

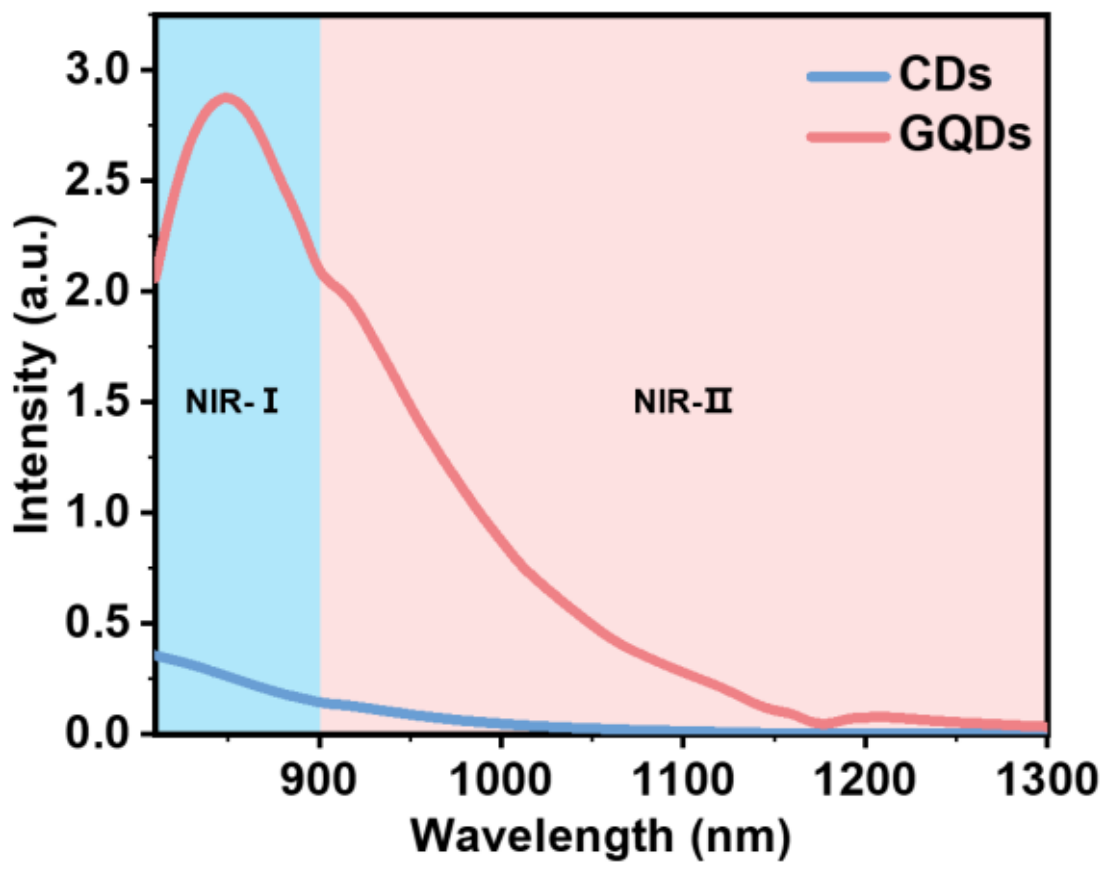

Figure S30. NIR emission spectra of CDs and GQDs. The emission spectra of CDs and GQDs in DMSO at 30 μg mL^{-1} under excitation at 808 nm were measured.

5. Bandgap measurement

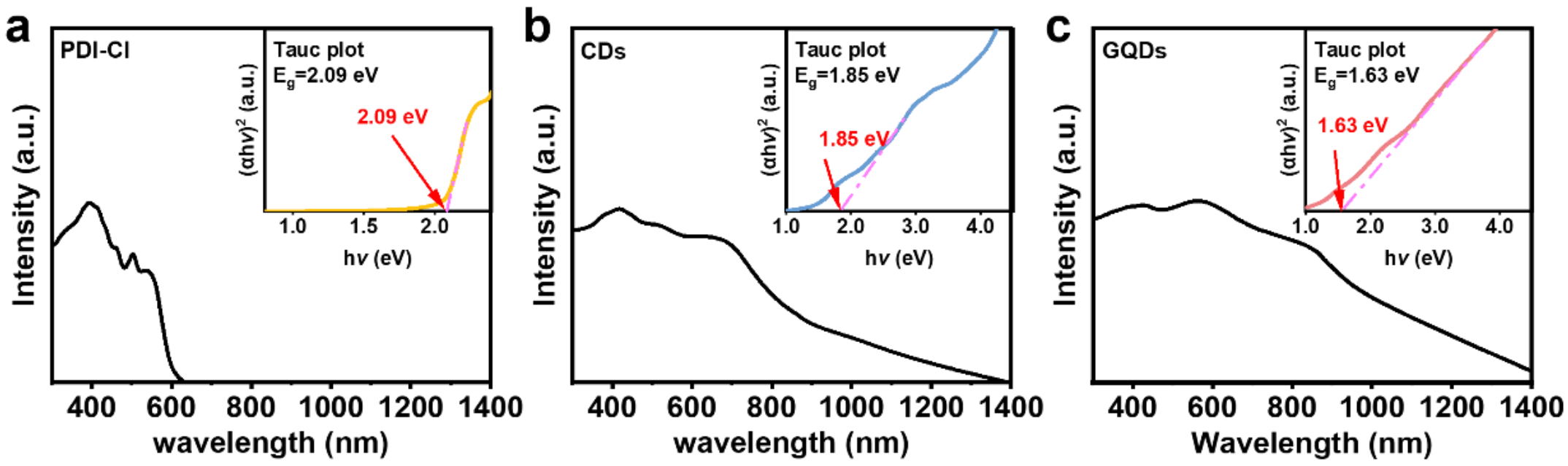

Figure S31. Bandgap determination via solid-state UV-Vis Spectroscopy. The solid-state absorption spectra of (a) PDI-Cl, (b) CDs, and (c) GQDs. Inset: the bandgap from a *Tauc* plot. The energy gap structure of organic semiconductors serves as a crucial basis for understanding their electronic transition mechanisms and optoelectronic properties. The discontinuous band structure of semiconductors enables electrons in excitons within the valence band (VB) to be excited and transition to the conduction band (CB) when irradiated by photons with energy equal to or greater than the bandgap width, while generating corresponding holes (h^+) in the VB. The energy gap structure of semiconductors can be measured and calculated through UV-vis absorption spectroscopy, Mott-Schottky analysis, and VB XPS measurements.

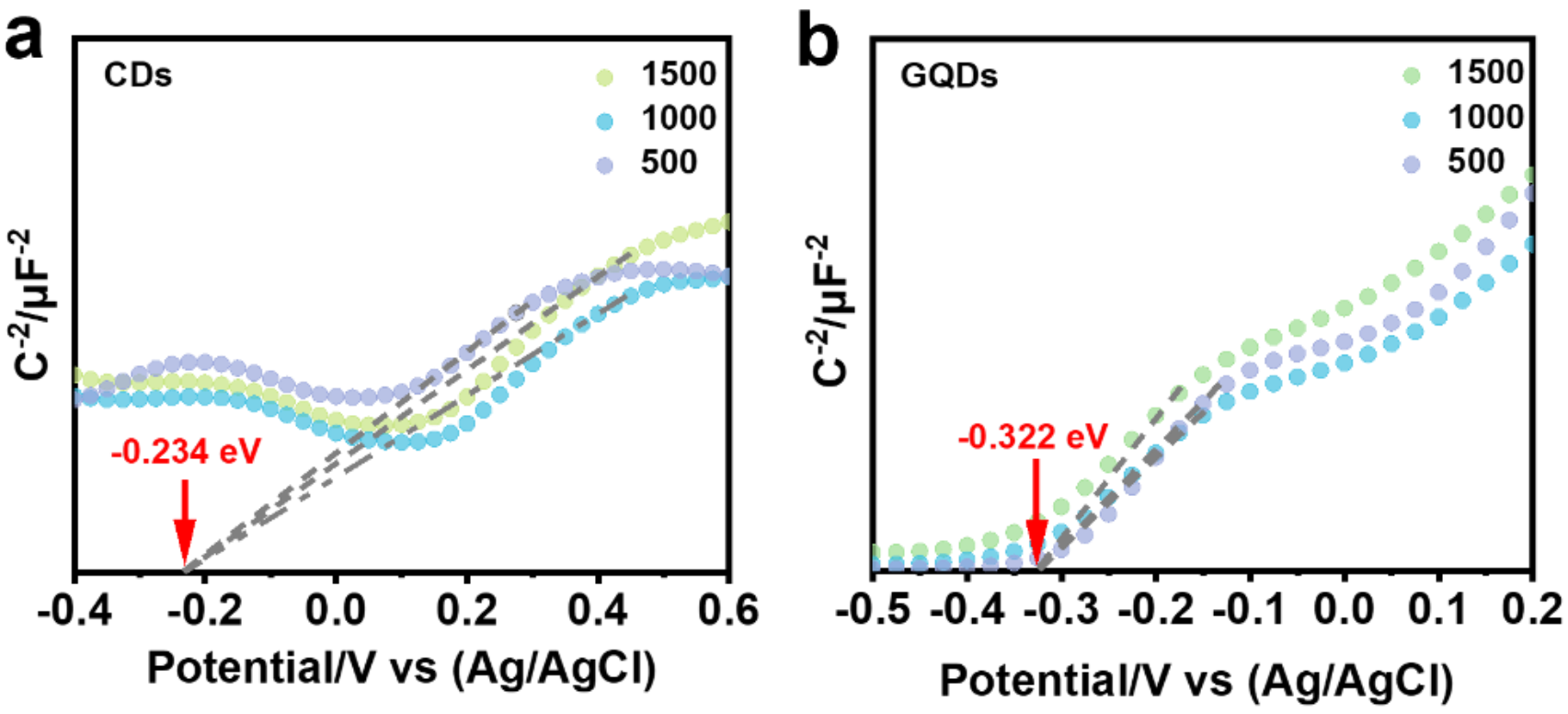

Figure S32. CB positions of CDs and GQDs. Conduction band potential Mott-Schottky measurements of (a) CDs and (b) GQDs using an electrochemical workstation (the detection frequencies are 500, 1000, and 1500 Hz, respectively). CB potential can be estimated by determining flat band potential by Mott-Schottky method. According to the formula $E_{(NHE)} = E_{(Ag/Ag+)} + 0.197$, the flat band potentials of the graphene quantum dots are calculated to be $E_{CDs} = -0.037$ and $E_{GQDs} = -0.125$. Furthermore, both materials exhibited positive slopes in their Mott-Schottky plots, confirming their n-type semiconductor characteristics.

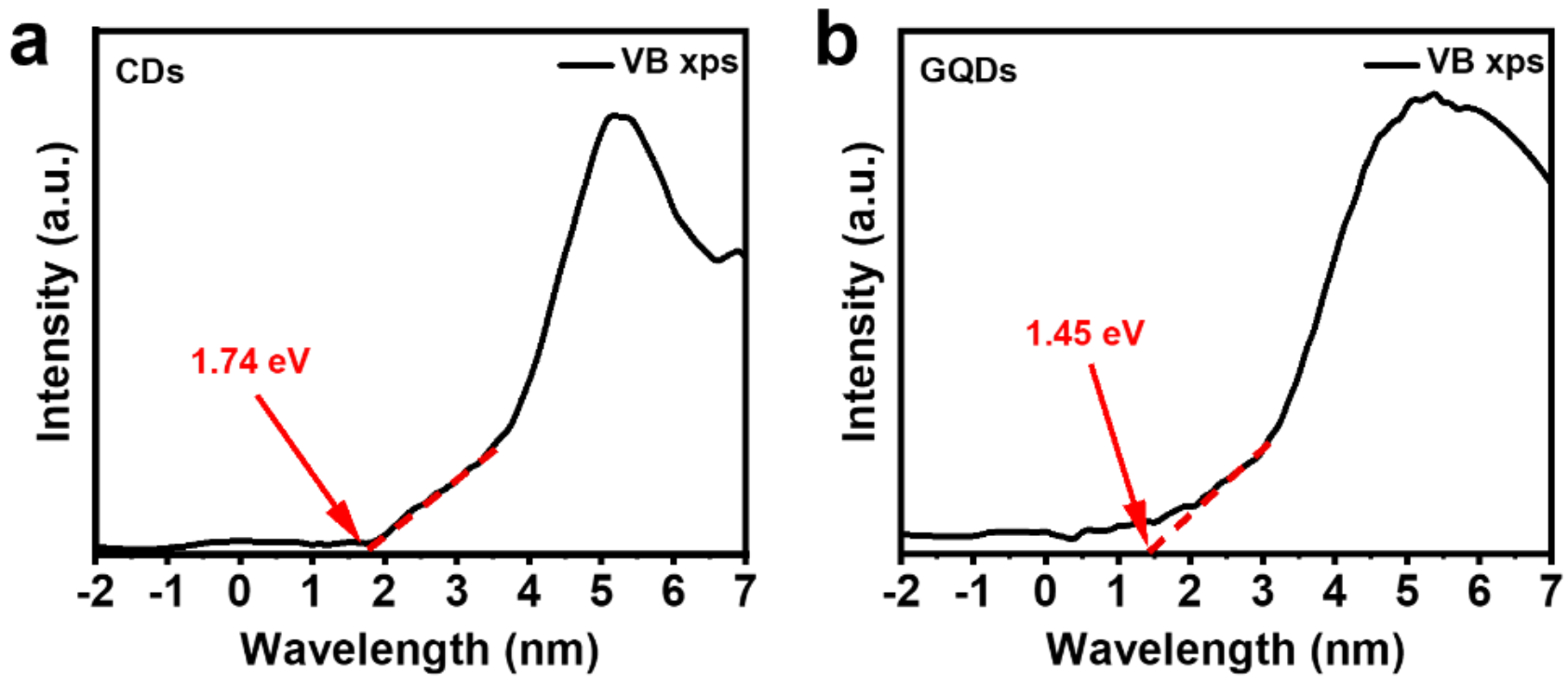

Figure S33. VB positions of CDs and GQDs. Valence band positions of CDs and GQDs fitted by VB XPS. According to the formula $E_{(NHE)} = \psi + E_{(VB,\ XPS)} - 4.44$, where the instrument work function ψ is 4.5 W, it is calculated that $E_{CDs} = 1.80$ and $E_{GQDs} = 1.51$.

6. Theoretical calculations

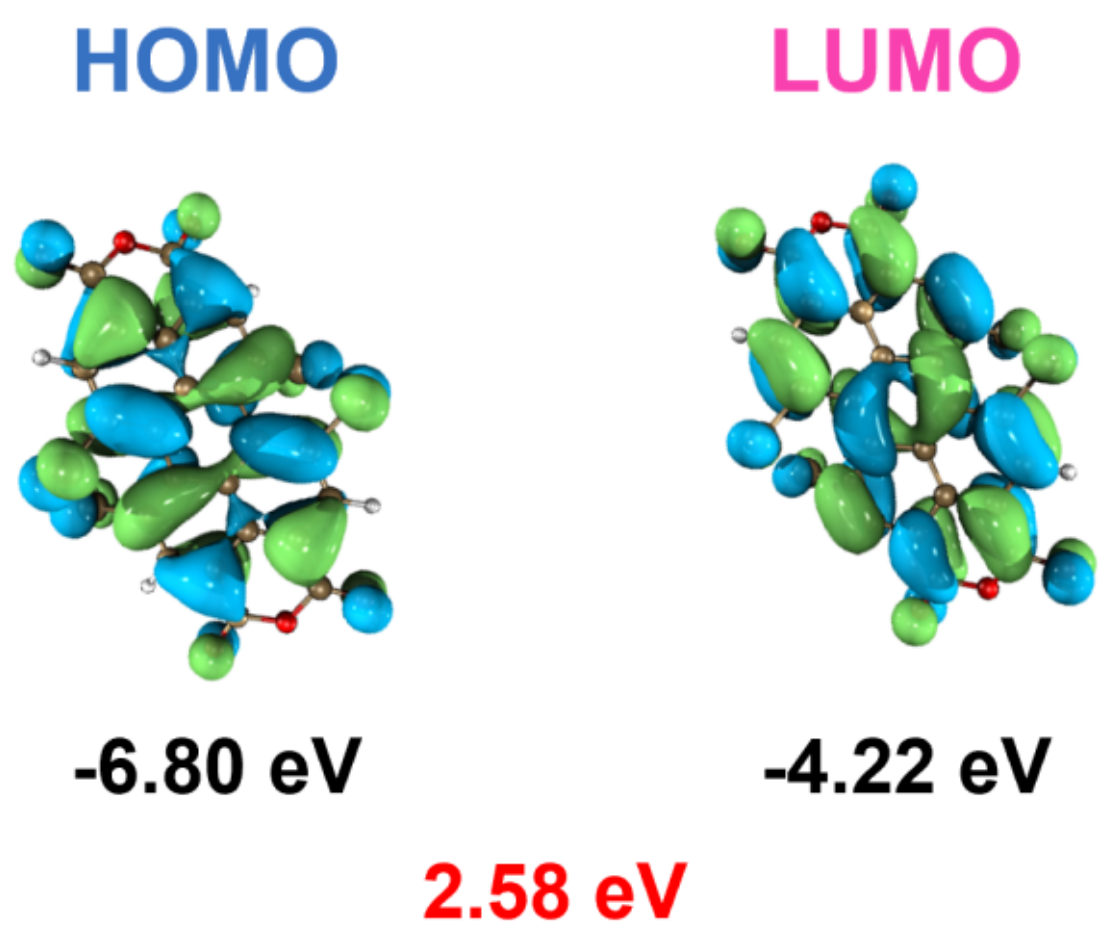

Figure S34. Ground-state isosurface maps of PDI-Cl. Molecular structures and calculated HOMO and LUMO of PDI-Cl.

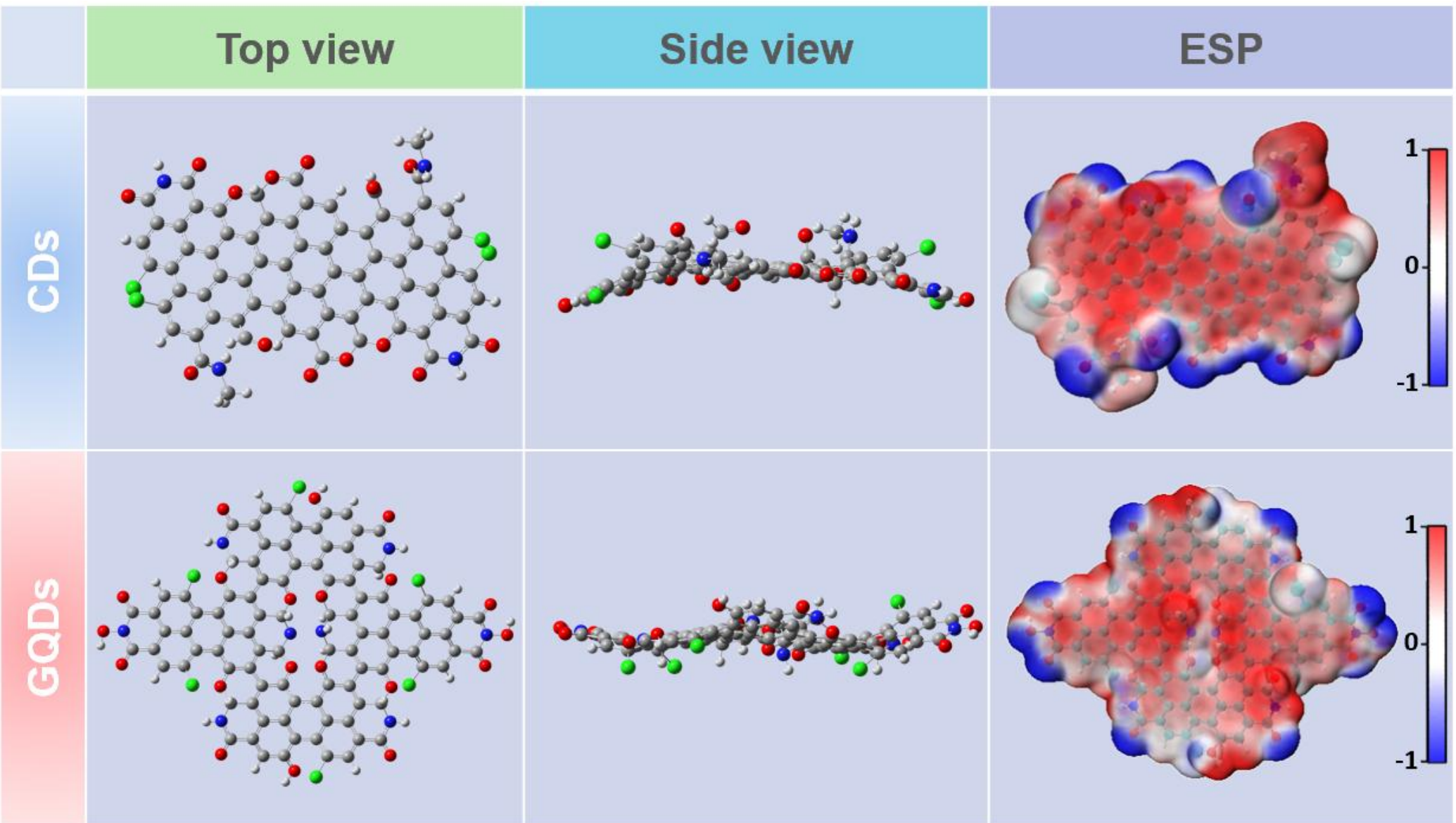

Figure S35. Ground-state model structure and ESP distribution. Top view, side view, and electrostatic potential map (ESP) of the molecular structures of model 1 for CDs and model 2 for GQDs after geometry optimization based on density functional theory (DFT) at the B3LYP/6-31G(d) level. The results revealed distinct structural characteristics: CDs exhibited planar carbon layers in side view with nearly co-planar alignment, whereas GQDs displayed curved lattice distortions. Electrostatic potential analysis demonstrated clear spatial separation between the inner π-conjugated system and edge groups in CDs. In contrast, GQDs showed discontinuous electrostatic potential surfaces due to intrinsic defects, which may significantly influence electron relaxation during excitation processes.

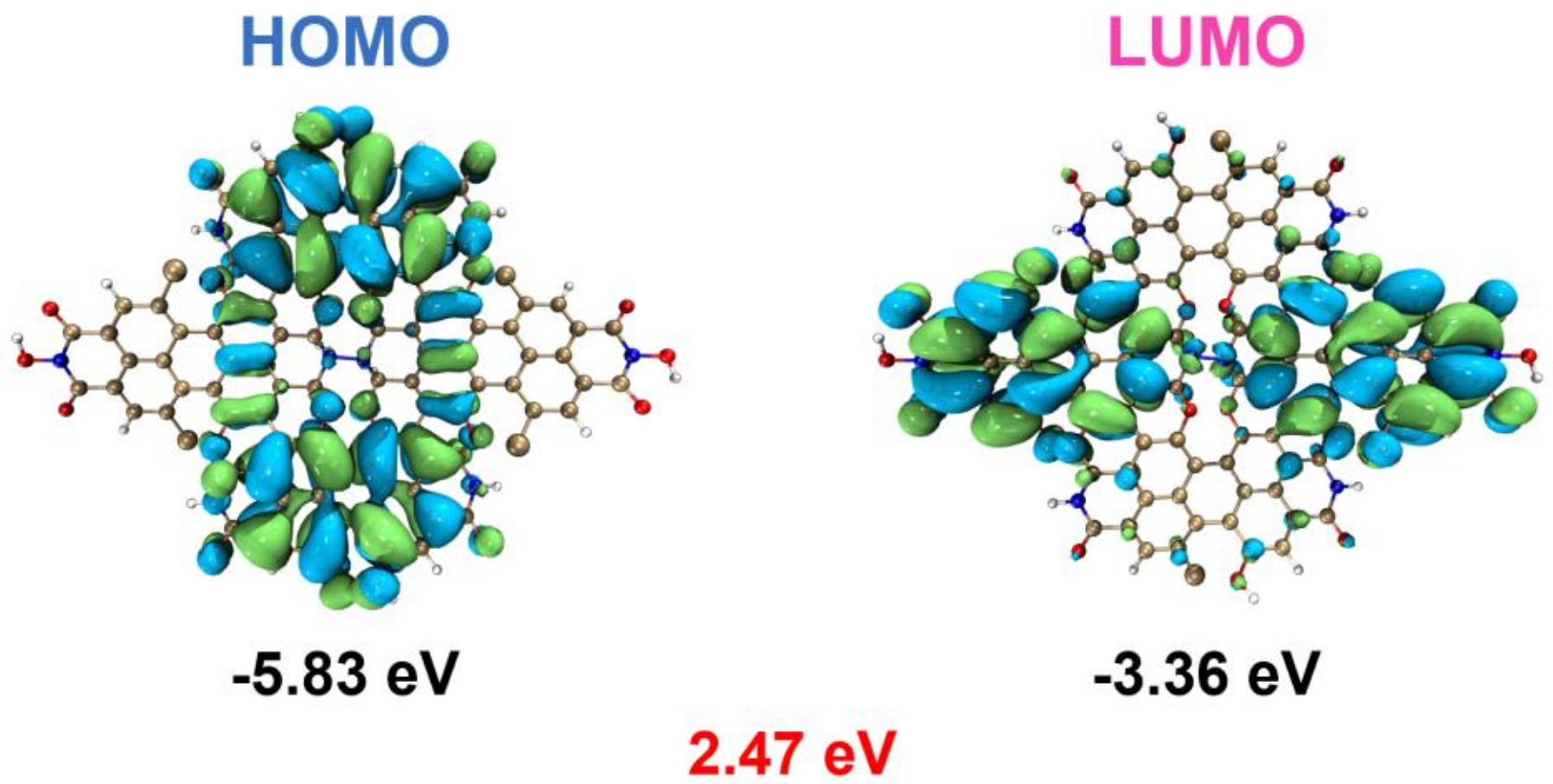

Figure S36. Ground-state isosurface maps of ir-GQDs. Molecular structures and calculated HOMO and LUMO of radical-free model 4 for ir-GQDs.

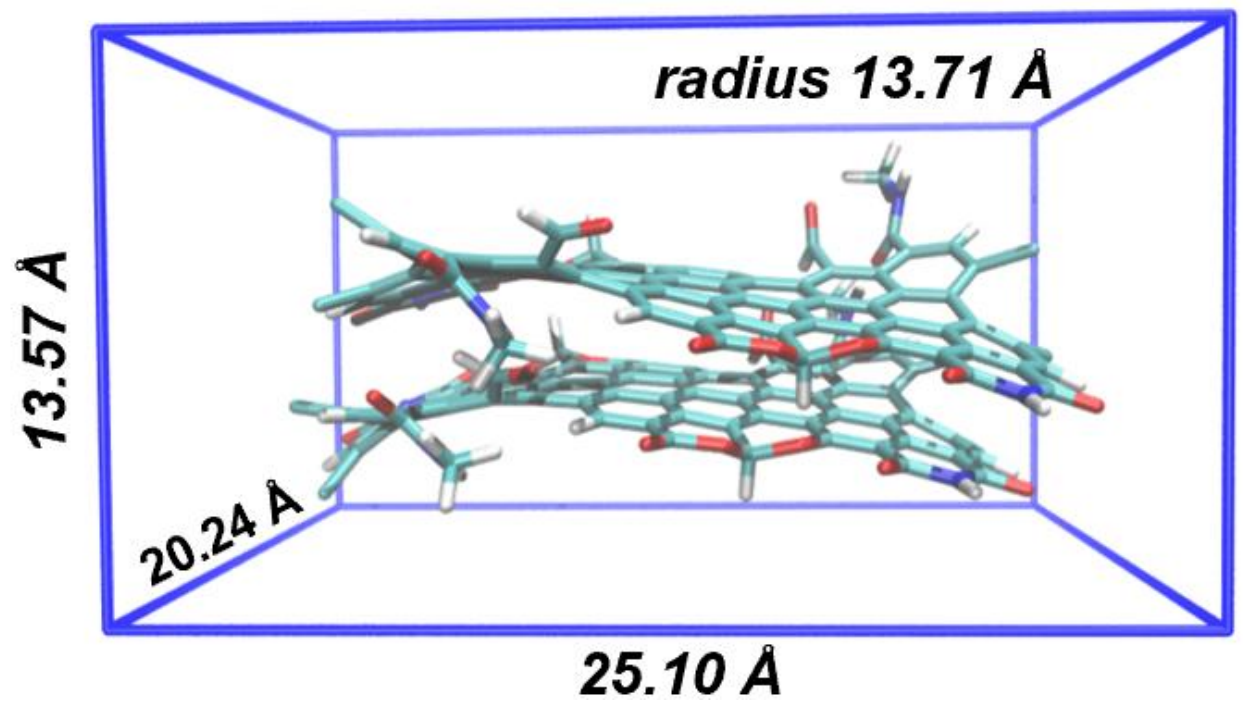

Figure S37. Three-dimensional rendering of bilayer CDs. Schematic diagram of the size of the dimer model for CDs by Multiwfn software. CDs displayed a larger interlayer distance (~1.4 nm), suggesting a stacked structure composed of 6-8 graphene-like layers.

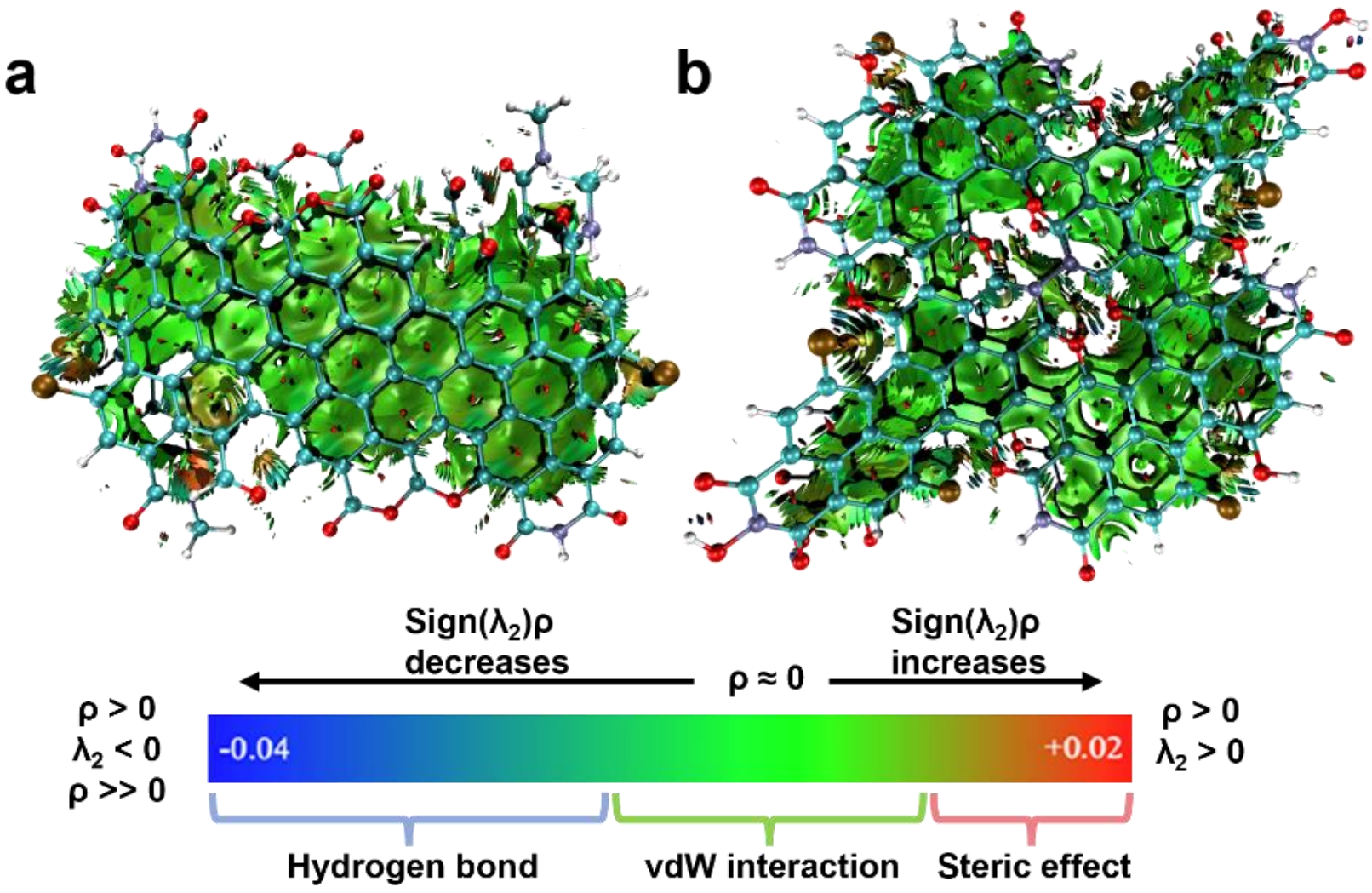

Figure S38. Interlayer interaction characterization of CDs and GQDs. Interaction region indicator (IRI) isosurface maps of dimer models for (a) CDs and (b) GQDs at the B3LYP/6-31G(d) level of theory. It was clear that the interaction between the graphene-like layers in GQDs was mainly due to the van der Waals (vdW) interaction. Note that these vdW regions were predominantly localized between sp^2 conjugation domains.

7. Photophysical properties analysis

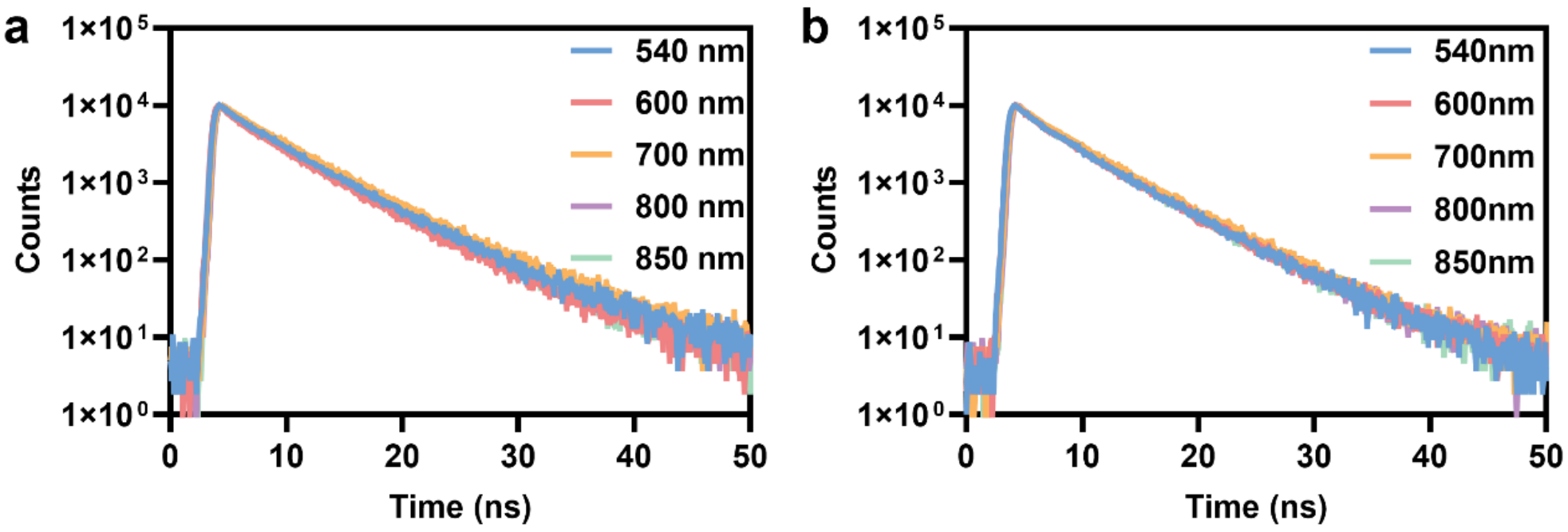

Figure S39. Solvent-dependent TCSPC signals of CDs. PL decay curves of CDs in (a) DMSO and (b) DMSO/methanol mixed solution under 510 nm excitation and monitored at 540, 600, 700, 800, and 850 nm. The fluorescence lifetimes of CDs exhibited uniformity across distinct emission bands, with no significant alterations before and after methanol addition. These results suggest CDs has no intrinsic-stabilized radicals.

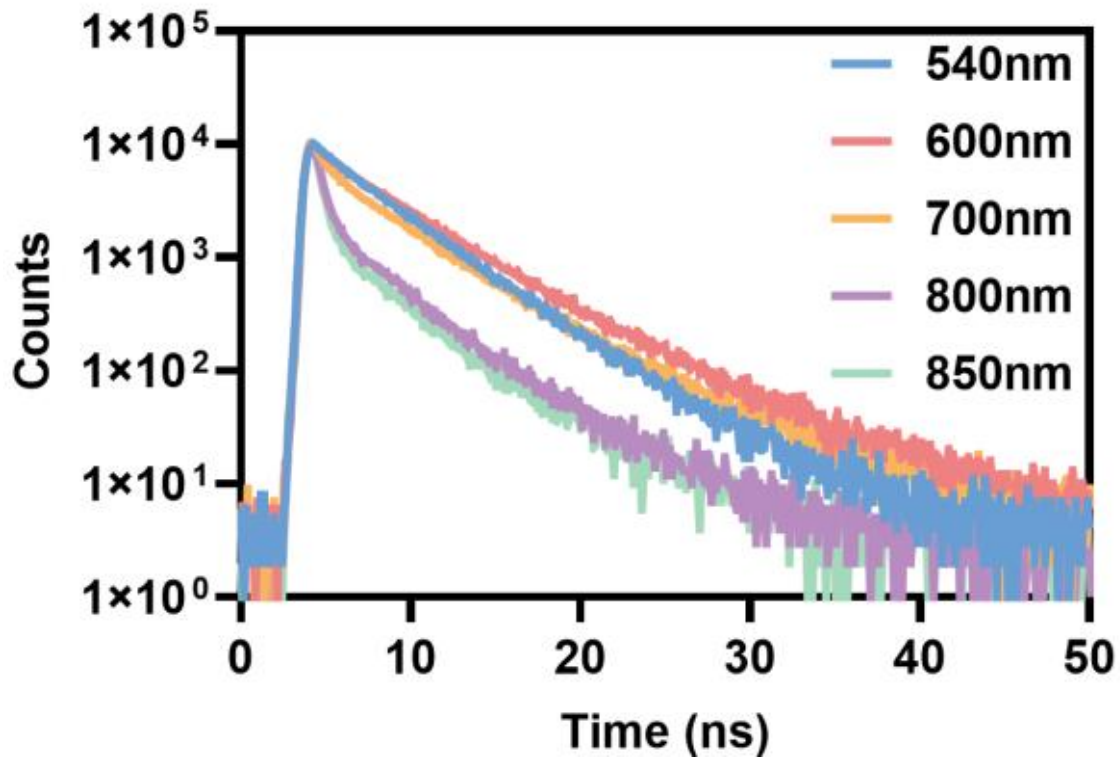

Figure S40. TCSPC signals of GQDs in mixed solution. PL decay curves of GQDs in DMSO/methanol mixed solution under 510 nm excitation and monitored at 540, 600, 700, 800, and 850 nm. Following the addition of trace methanol as a radical scavenger, wavelength-dependent fluorescence lifetime measurements revealed a significant prolongation of lifetimes at 540 and 600 nm, while the lifetimes of radical emission at 800 and 850 nm exhibited a marked reduction (Table S5).

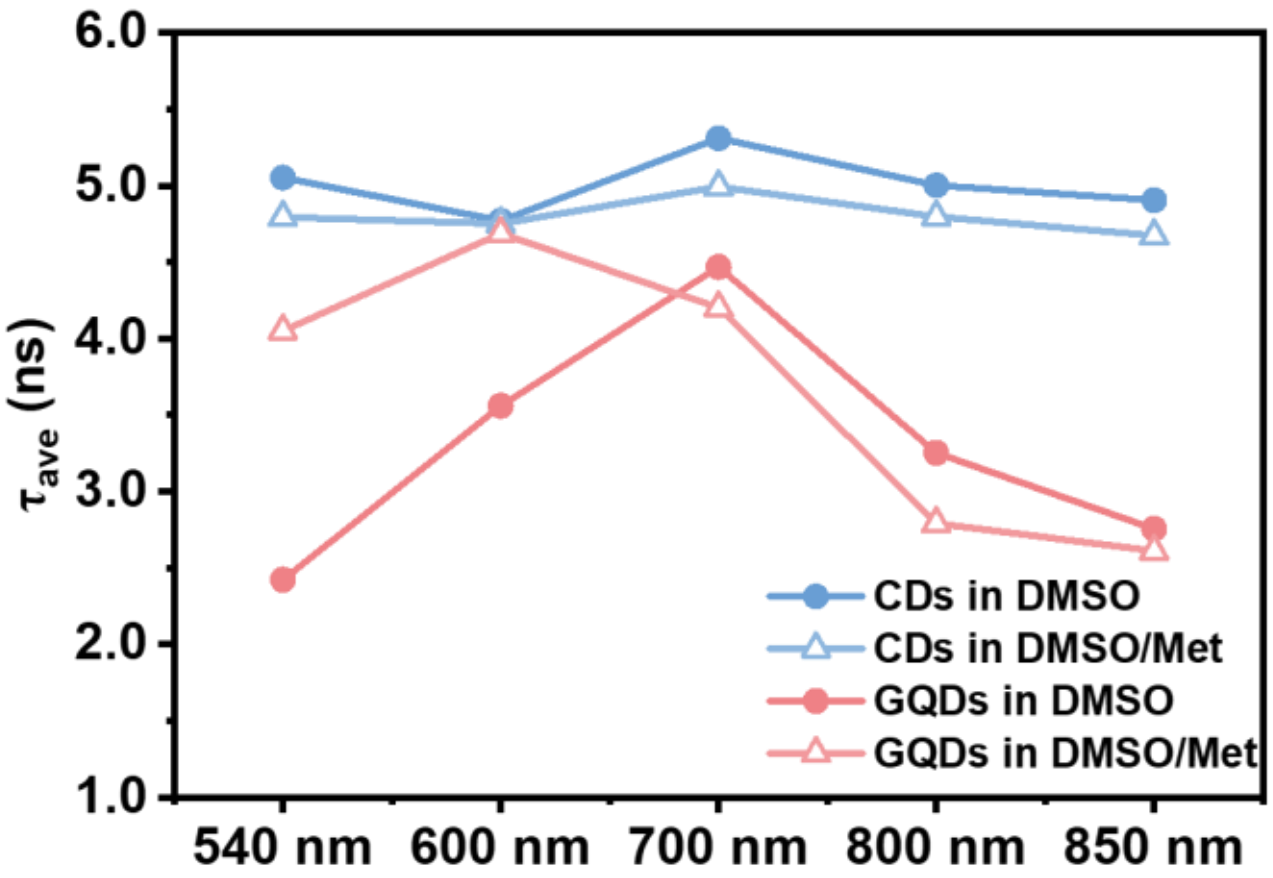

Figure S41. Fluorescence lifetime distributions of CDs and GQDs. Statistical plot of the average fluorescence lifetimes across emission bands for CDs and GQDs in different solvents under 510 nm excitation. The fluorescence lifetime decay signal serves as a critical indicator of excited-state dynamics and charge carrier behavior. Generally, a longer average decay time constant (τ_{ave}) correlates with higher photoluminescence quantum yield. The presence of methanol rapidly scavenges the intrinsic radicals, leading to suppressed radiative transition efficiency at 800-850 nm (associated with charge-transfer emission) while enhancing the emission of π-conjugated domain at 540-600 nm.

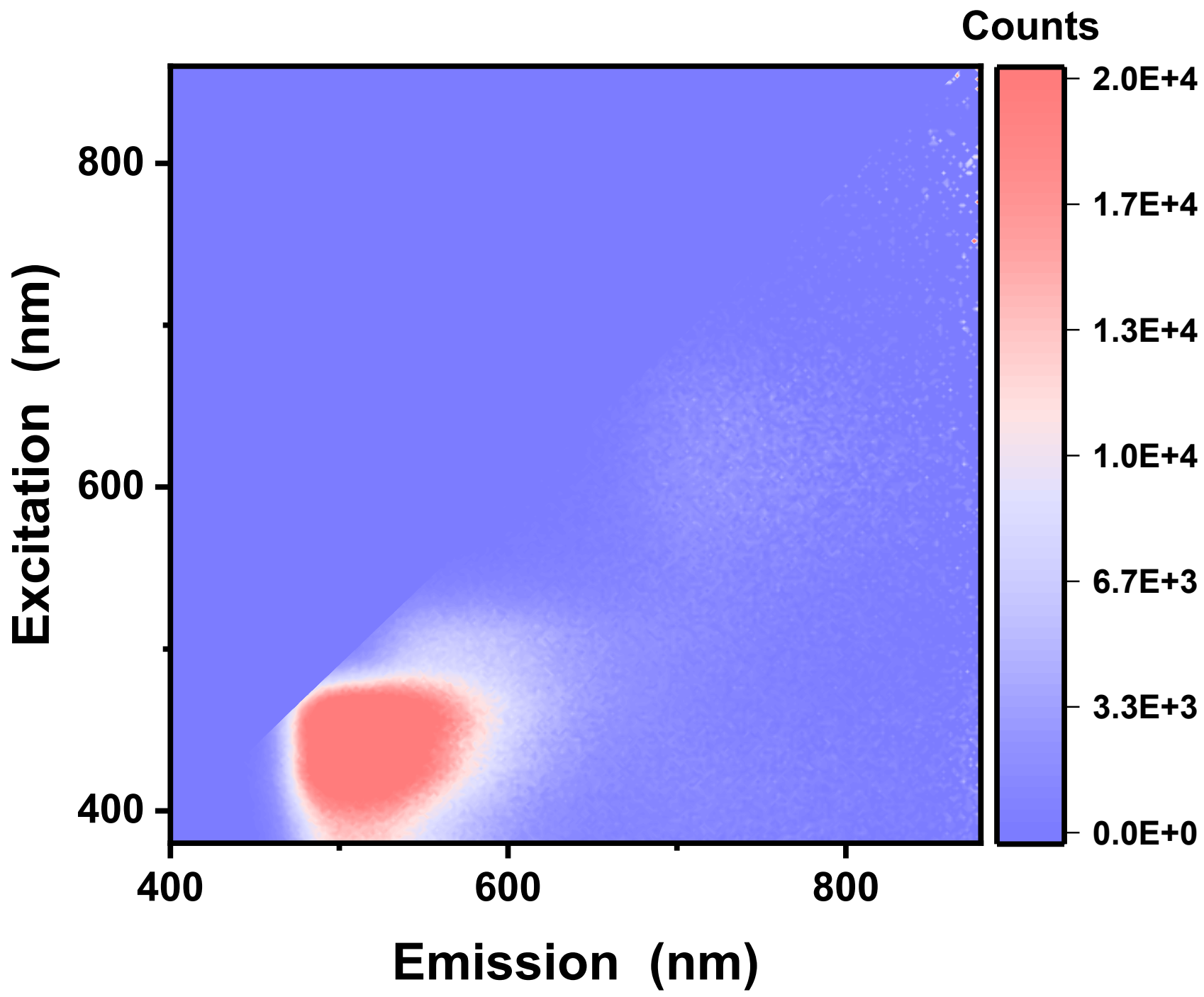

Figure S42. Fluorescence center analysis of CDs. Excitation-emission maps of CDs in DMSO solution. The result indicated the emission core of CDs was located at the visible region.

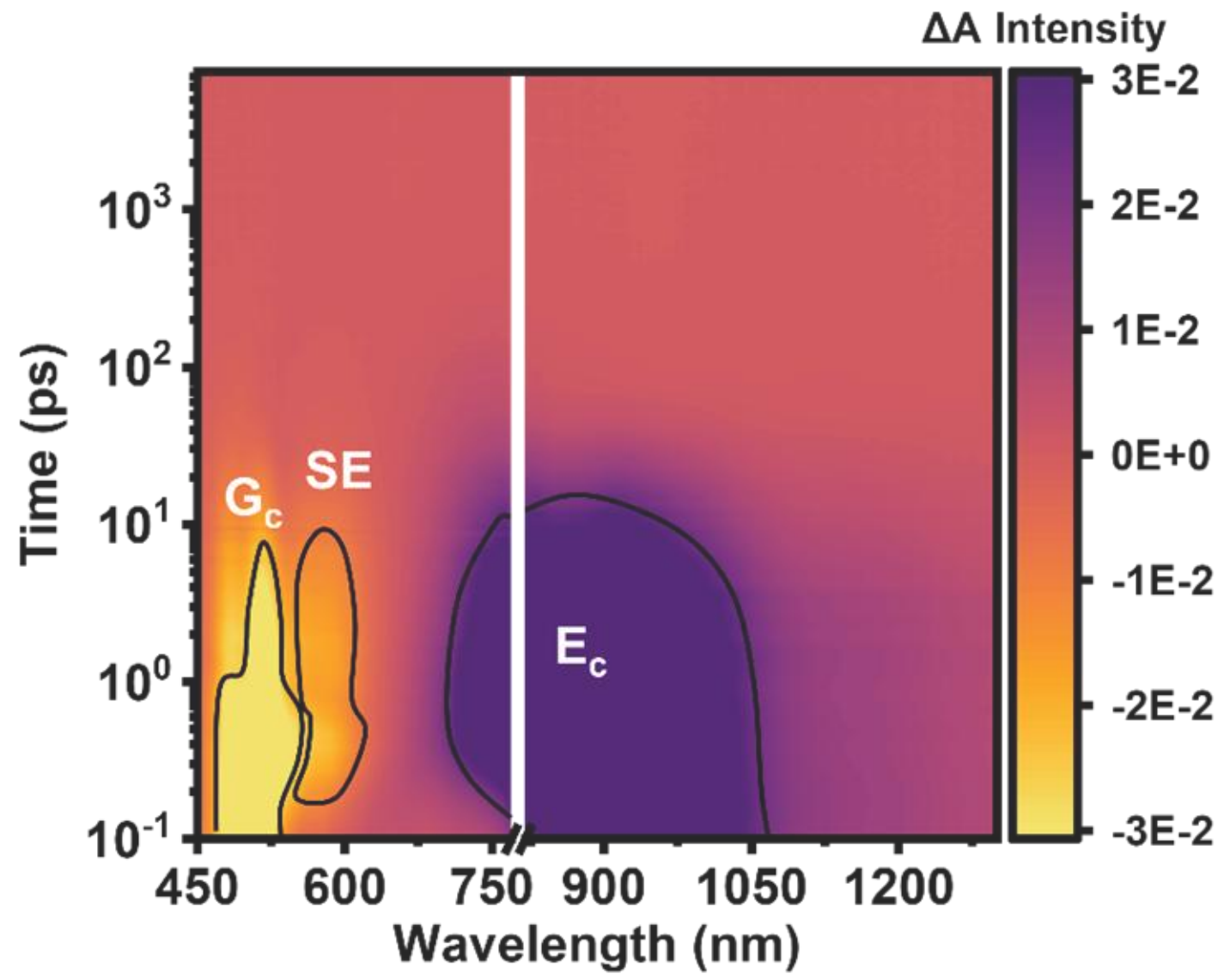

Figure S43. Excited state dynamics of the PDI-Cl. 2D pseudo-color maps of the TA spectrum of PDI-Cl with a pump wavelength of 400 nm. The corresponding excited-state absorption (ESA) appeared in the 700-1100 nm region.

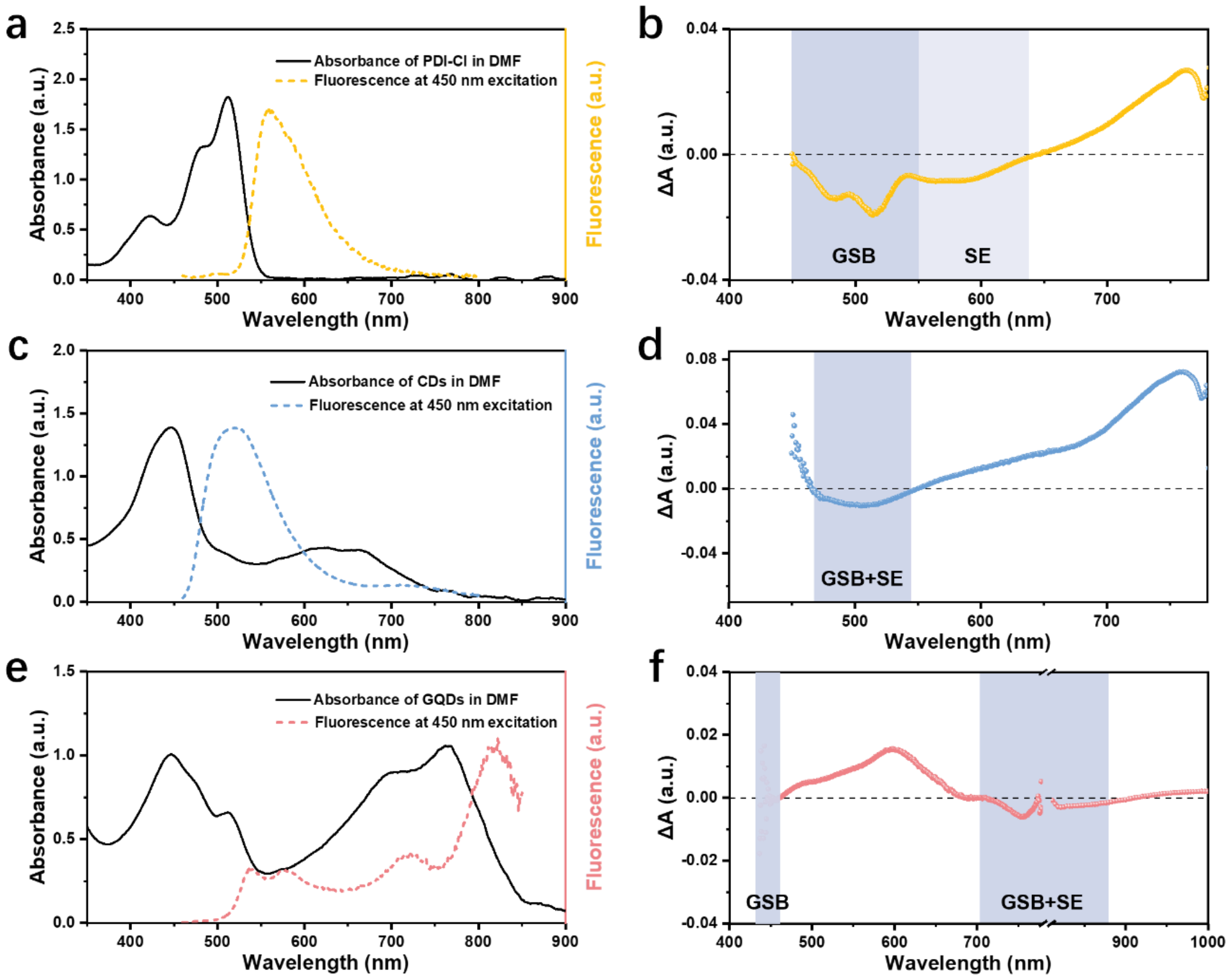

Figure S44. Assignment of TA signals. Steady-state absorption spectra, fluorescence spectra, and TA spectra (10 ps after excitation) of (a), (b) PDI-Cl, (c), (d) CDs, and (e), (f) GQDs in DMSO. The assignment of TA spectra was achieved by correlating steady-state absorption spectra with corresponding fluorescence spectra. For PDI-Cl in DMSO, the bleach signal at 450-550 nm perfectly matched the ground-state absorption profile in both spectral shape and peak position, unambiguously assigning it to ground-state bleach (GSB). The signal at 550-640 nm can be attributed to stimulated emission (SE) from molecular excited states. Similarly, CDs exhibited the partial spectral overlap between fluorescence and absorption, assigning the bleach signals of 460-550 nm as a convolution of both SE and GSB. For GQDs, their steady-state absorption exhibited weak visible absorption and strong near-infrared absorption. Consequently, in TA spectra, the weak band-edge signal at 450 nm was assigned to GSB of edge-conjugated states, while the signals in the 700-900 nm range originate from both GSB and SE induced by charge transfer (CT) processes.

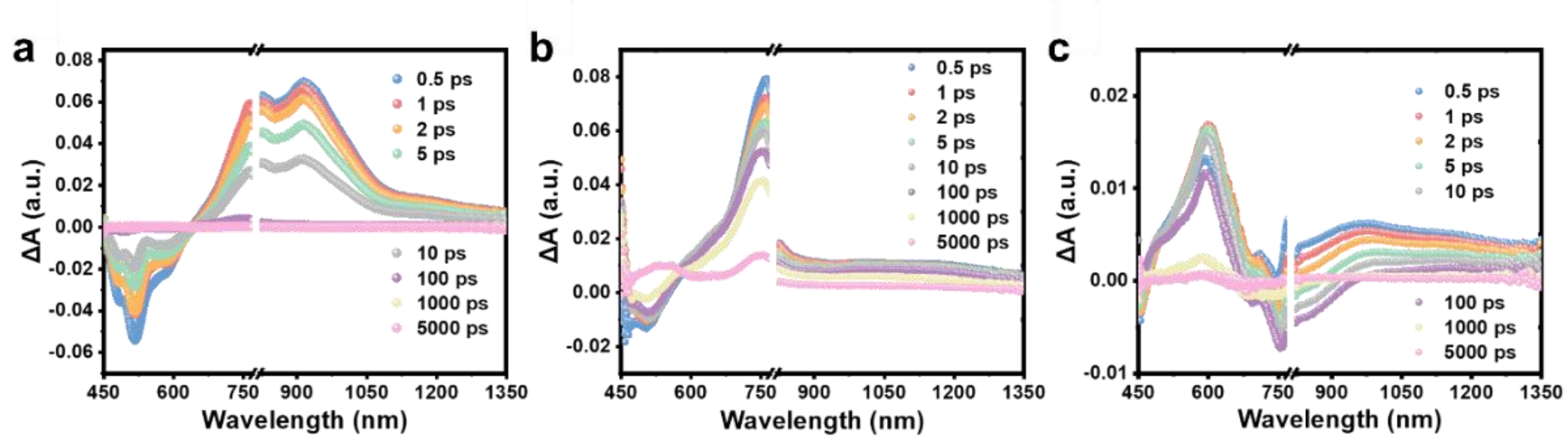

Figure S45. Bleach signal kinetics of PDI-Cl, CDs, and GQDs. TA spectra of (a) PDI-Cl, (b) CDs, and (c) GQDs in DMSO at indicated delay times (λ_{pump} = 400 nm).

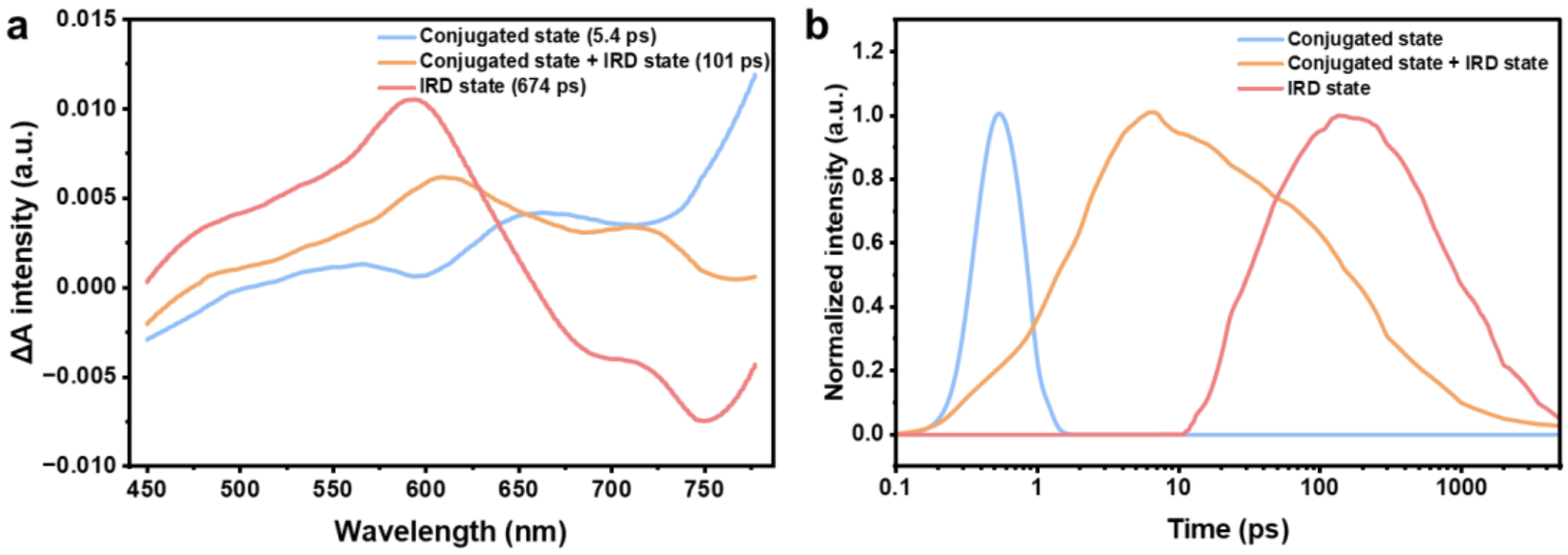

Figure S46. (a) A global fitting intensity and (b) normalized kinetic evolution traces of the conjugated state (5.4 ps), the conjugated state + IRD state (101 ps), and the IRD state (674 ps) in GQDs upon photoexcitation.

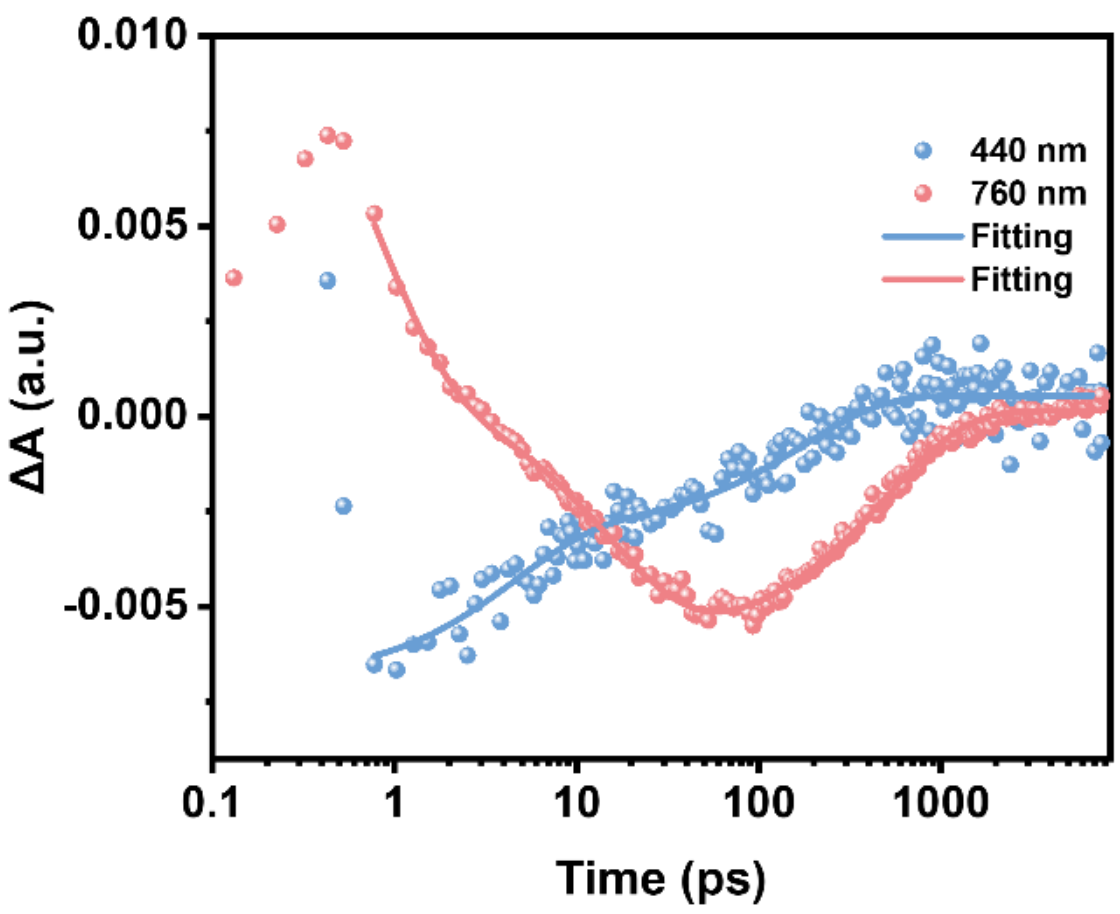

Figure S47. Charge transfer related signals in GQDs. TA kinetic traces of GQDs collected at 440, and 760 nm at a pump wavelength of 400 nm. Dots are experimental points, and solid lines are fittings. Decay dynamic fitting results at 440 nm and 760 nm revealed a negative correlation between GSB intensities within 40 ps, indicating charge transfer from π-conjugated domain to IRD domain.

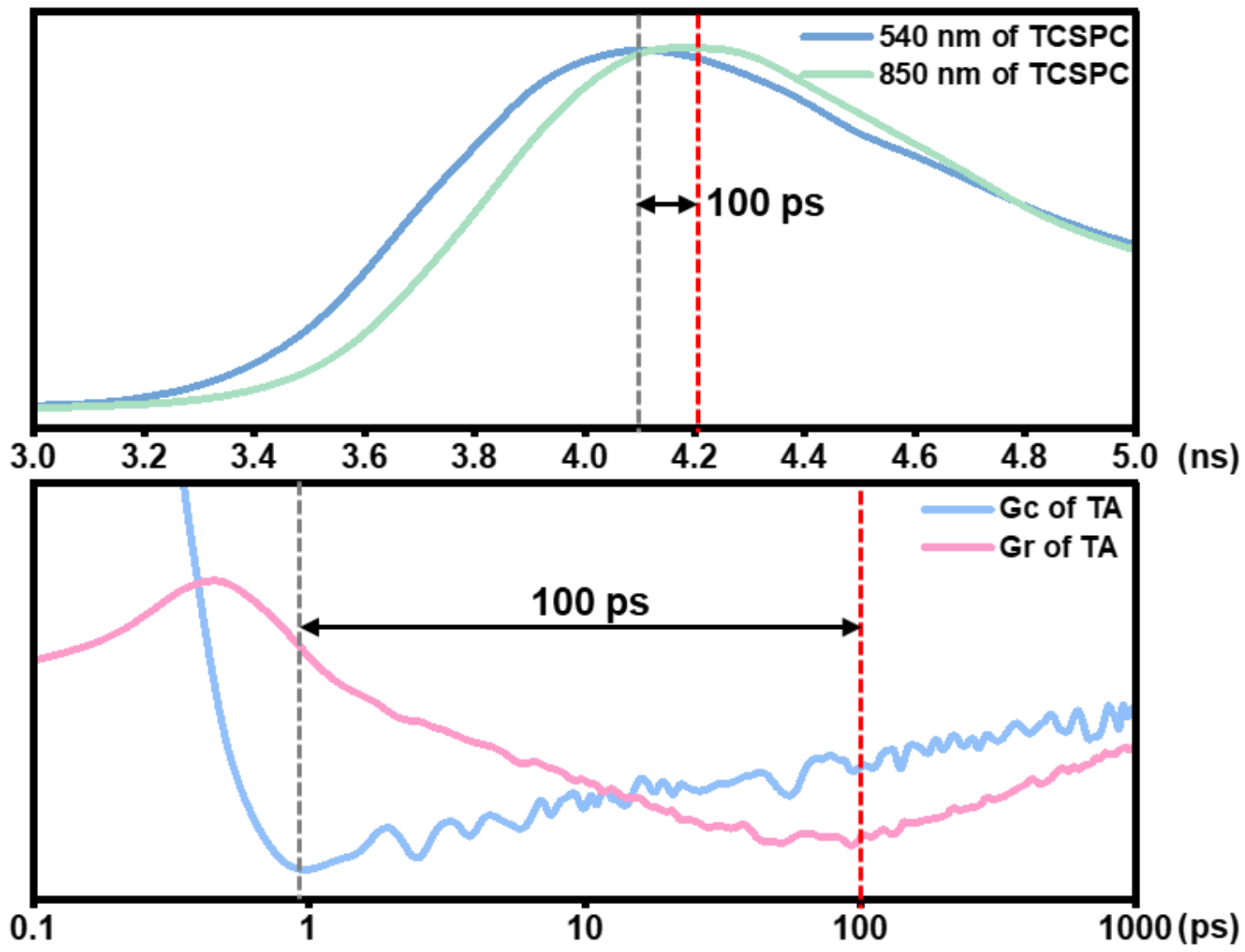

Figure S48. Charge transfer dynamics in GQDs revealed by TCSPC and TA spectroscopy. Up: Decay kinetics (excited at 510 nm) of GQDs were monitored at 540 nm, and 850 nm in DMSO at 60 μg mL^{-1}. Down: TA kinetic traces of GQDs collected at 440 nm (Gc for GQDs), 760 nm (Gr for GQDs), at a pump wavelength of 400 nm. Both measurement techniques consistently reveal that charge transfer processes in GQDs occur within 100 ps after excitation.

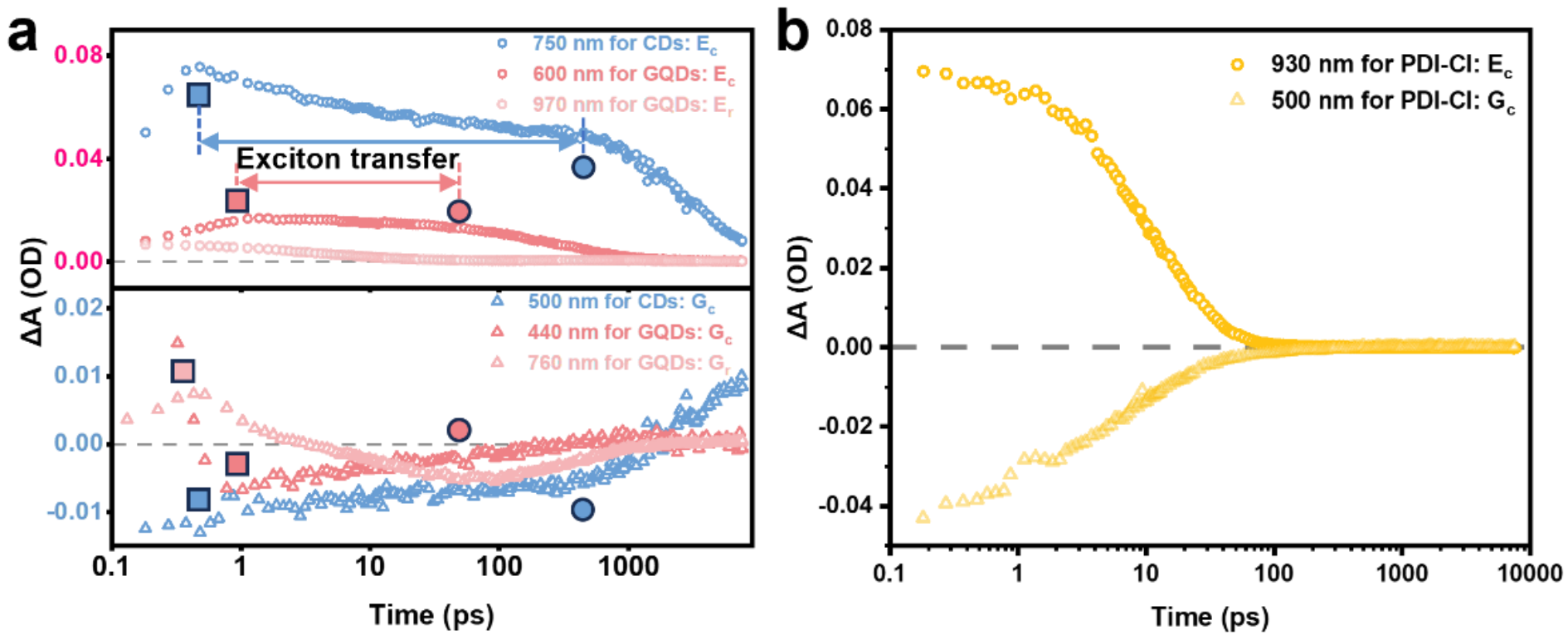

Figure S49. Analysis of exciton dynamics in PDI-Cl, CDs, and GQDs. (A) TA kinetic traces of GQDs collected at 440 nm (G_c for GQDs), 500 nm (G_c for CDs), 600 nm (E_c for GQDs), 750 nm (E_c for CDs), 760 nm (G_r for GQDs), and 970 nm (E_r for GQDs) at a pump wavelength of 400 nm. (B) The decay kinetics with logarithmic fitting in PDI-Cl at a pump wavelength of 400 nm. The exciton dynamics during photoexcitation involved three sequential processes: 1) exciton dissociation generating separated electrons and holes (prior to the labeled with "□"), 2) charge carrier migration (prior to the labeled with "○"), and 3) eventual electron-hole recombination (subsequent to the labeled with "○")[8]. Notably, the exciton dynamics of PDI-Cl completed within 100 ps, with no significant exciton dissociation signals. This phenomenon can be attributed to ultrafast Frenkel exciton coupling (<0.1 ps) in the compact conjugated domain of PDI-Cl.

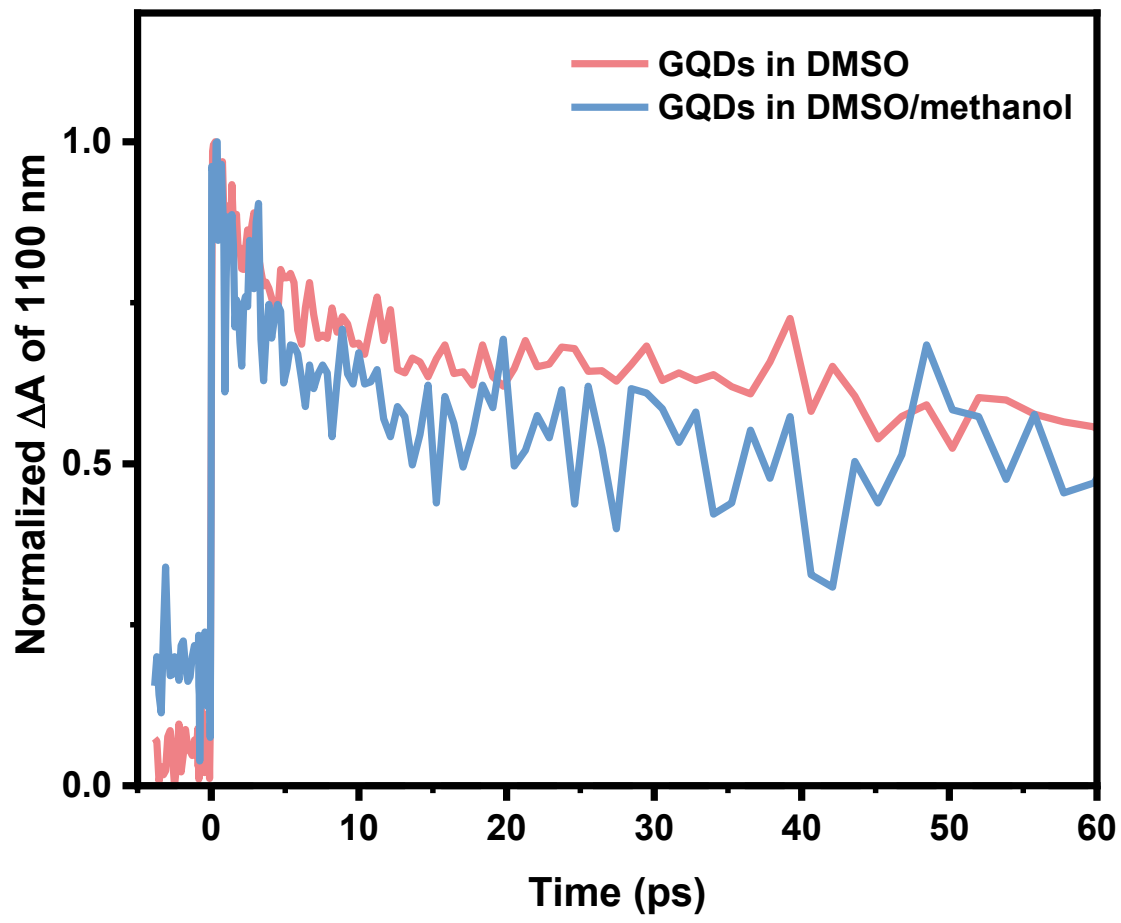

Figure S50. Relaxation dynamics of TA following radical quenching. The transient relaxation dynamics of GQDs in DMSO and DMSO/methanol mixture, with a pump wavelength of 700 nm. To elucidate interfacial charge transfer mechanisms, methanol was introduced as a hole scavenger. Kinetic analysis of the 1100 nm signal decay revealed a marked acceleration following methanol addition, which we attribute to the enhanced extraction efficiency of IRD domain through methanol-mediated scavenging processes.

8. Determination of exciton transfer

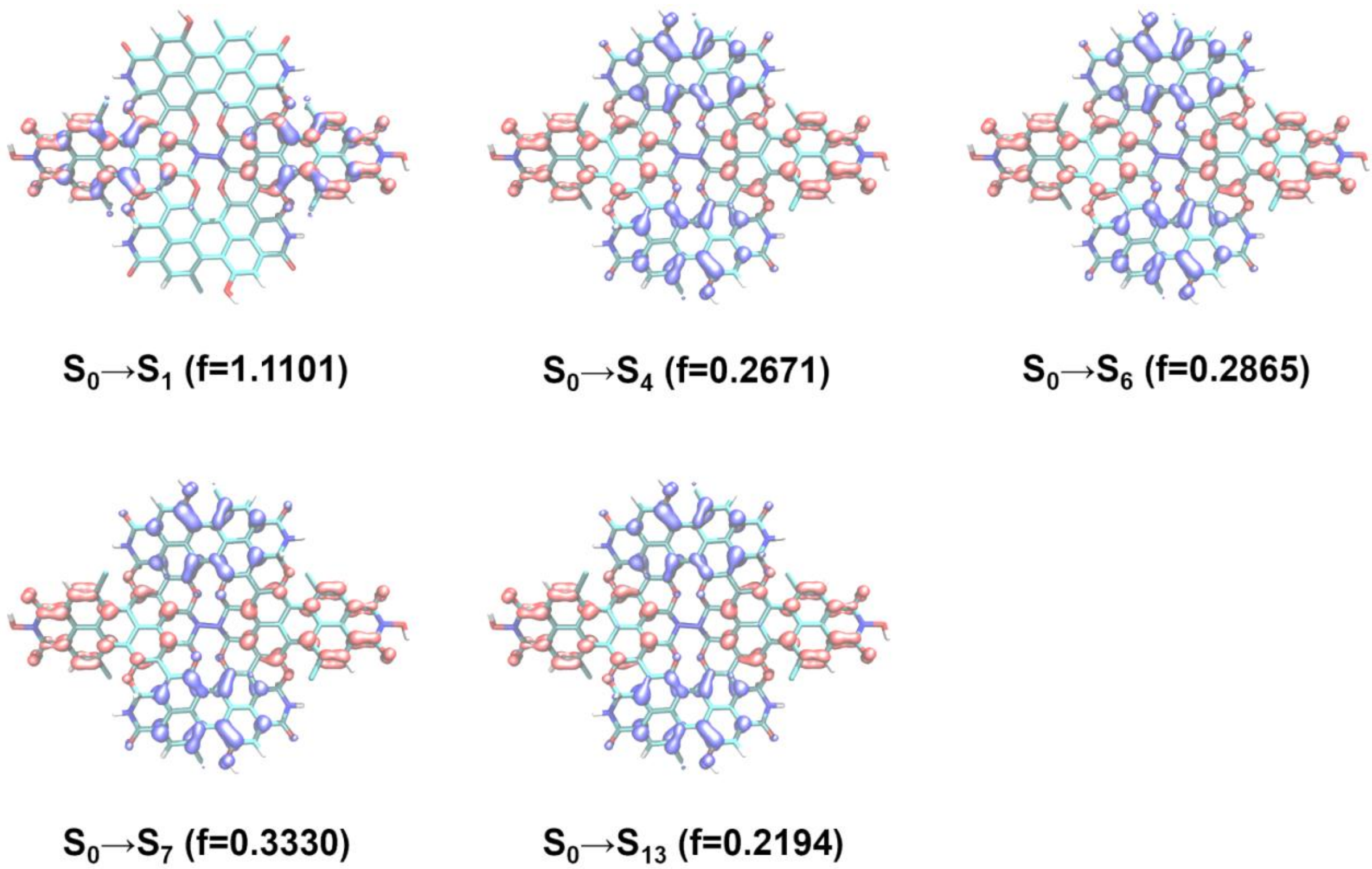

Figure S51. Excitation analysis of ir-GQDs. Excited-state isosurface maps of model 4 (ir-GQDs, the red area represents the electron distribution and the blue area represents the hole distribution). To investigate the influence of radical at the core on the excitation dynamics of GQDs, we performed electron-hole excitation analysis. The results demonstrate that ir-GQDs exhibit significantly enhanced oscillator strengths compared to GQDs (Figure S40). The $S_0 \rightarrow S_5$ transition is predominantly governed by localized π-π* excitation (LE) at the edge perylene-conjugated moieties, which plays a dominant role in the excitation process. Other excitation pathways are controlled by charge transfer (CT) excitations between adjacent perylene-conjugated π-systems.

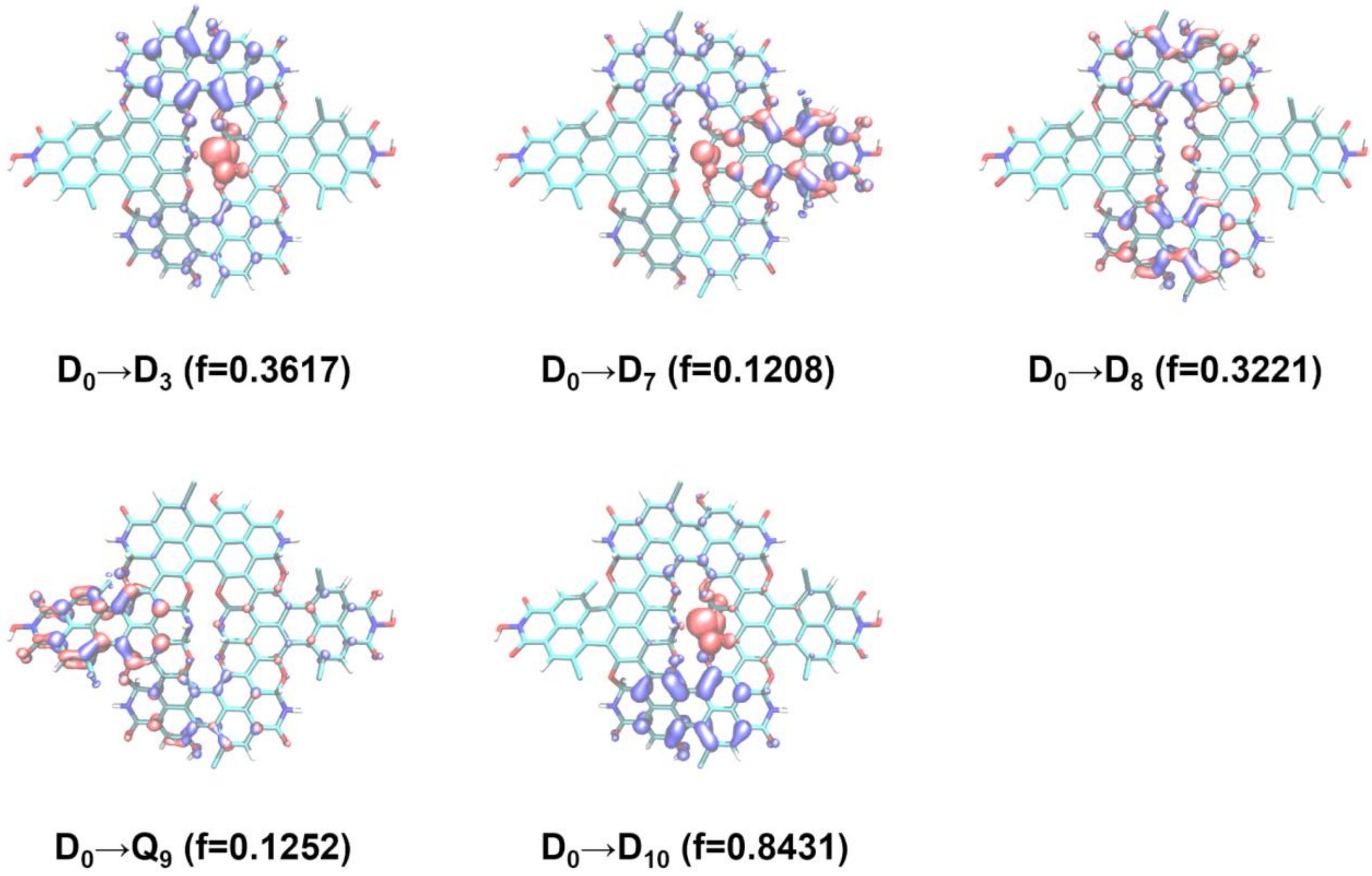

Figure S52. Excitation analysis of GQDs. Excited-state isosurface maps of model 2 (GQDs, the red area represents the electron distribution and the blue area represents the hole distribution). To further analyze the spatial distribution of electron-hole pairs in GQDs upon excitation, we performed electron-hole excitation analysis. The results demonstrate that the $D_0 \rightarrow D_1$ and $D_0 \rightarrow D_3$ transitions are predominantly governed by charge transfer (CT) excitations from the edge perylene-conjugated states to the central radical sites, which play a dominant role in the excitation process. In contrast, the $D_0 \rightarrow Q_2$, $D_0 \rightarrow Q_4$, and $D_0 \rightarrow Q_5$ transitions are primarily controlled by localized π-π* excitations (LE) at the edge-conjugated moieties, yet these contribute negligibly to the overall excitation process.

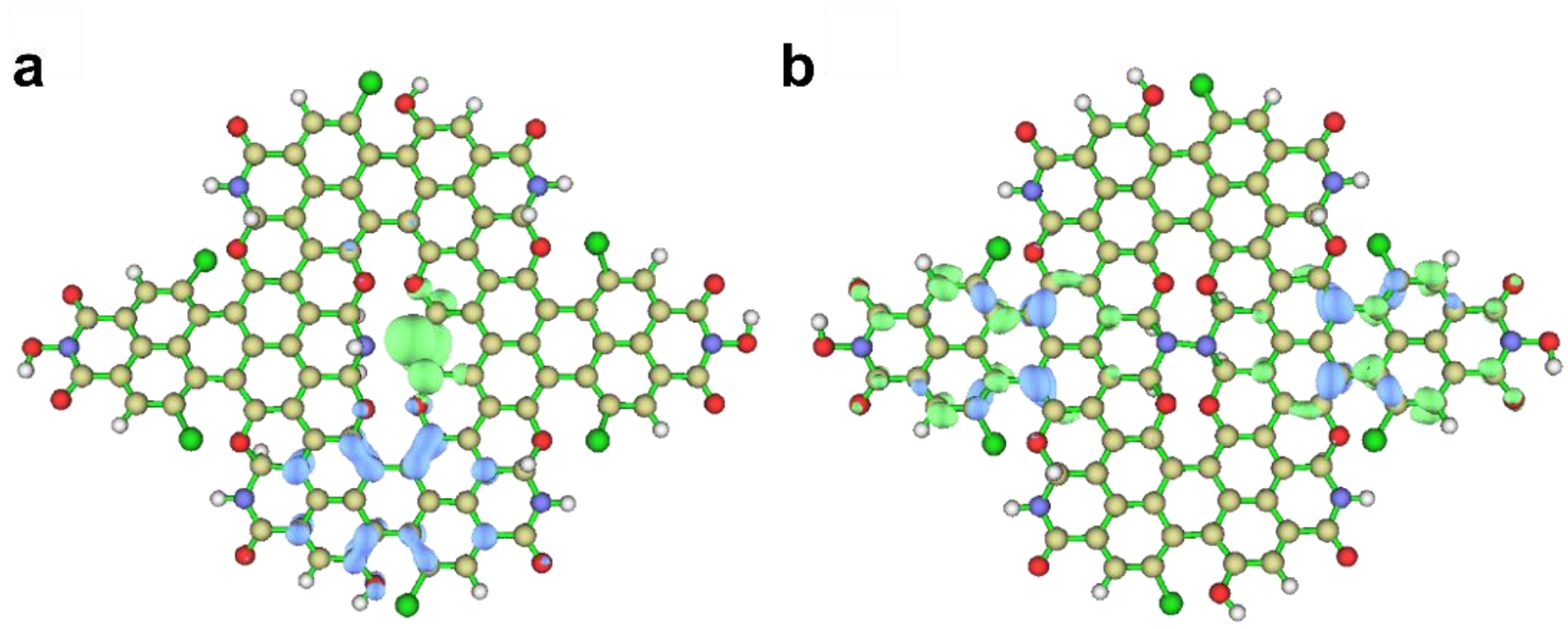

Figure S53. Charge distribution in GQDs upon excitation. The charge density difference (CDD) plots between excited and ground states for the dominant transition modes of (a) model 2 and (b) model 4, respectively. The blue regions indicate depletion of electron density, while the green regions represent accumulation of electron density. During photoexcitation, a pronounced electron transfer occurs from the edge-conjugated states to the central radical defect in GQDs. In contrast, the ir-GQDs exhibits no significant charge transfer process.

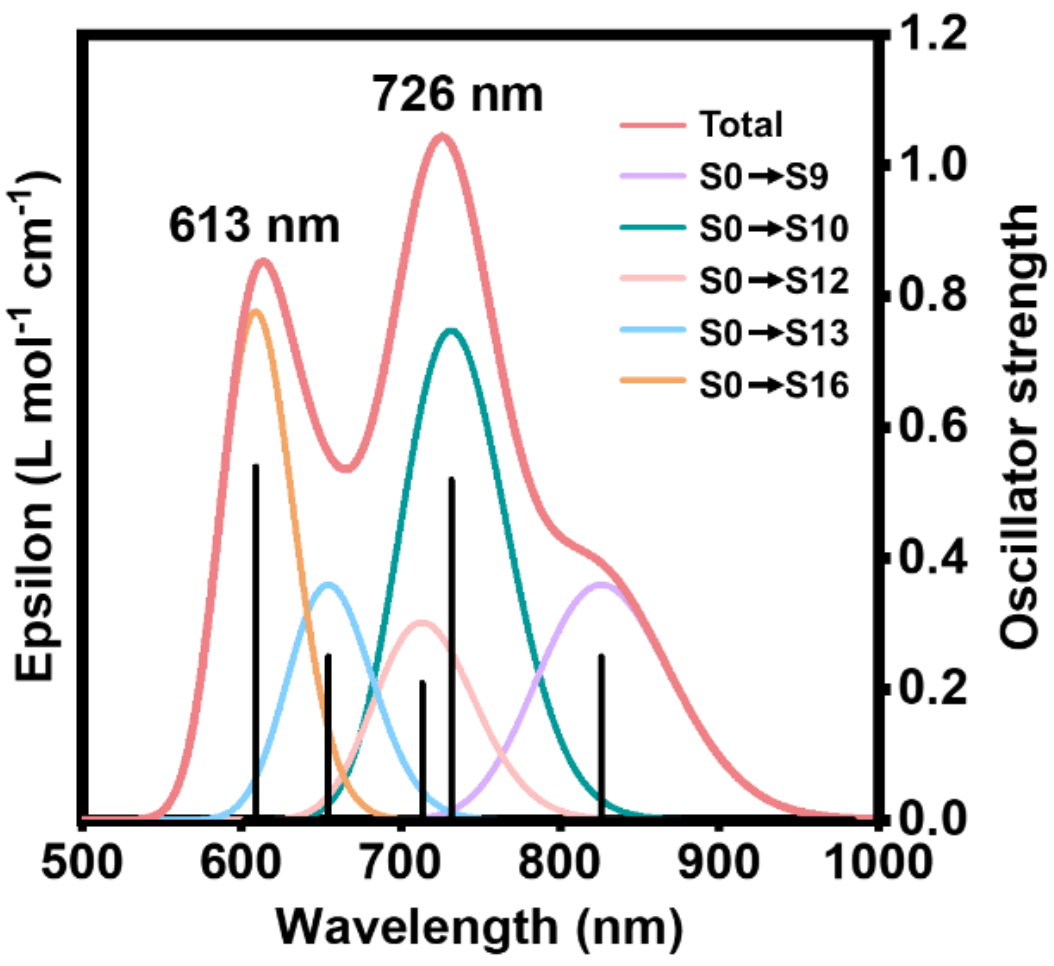

Figure S54. Simulated absorption spectra and oscillator strength of model 3 of GQDs.

9. Characterization of GQD micelles

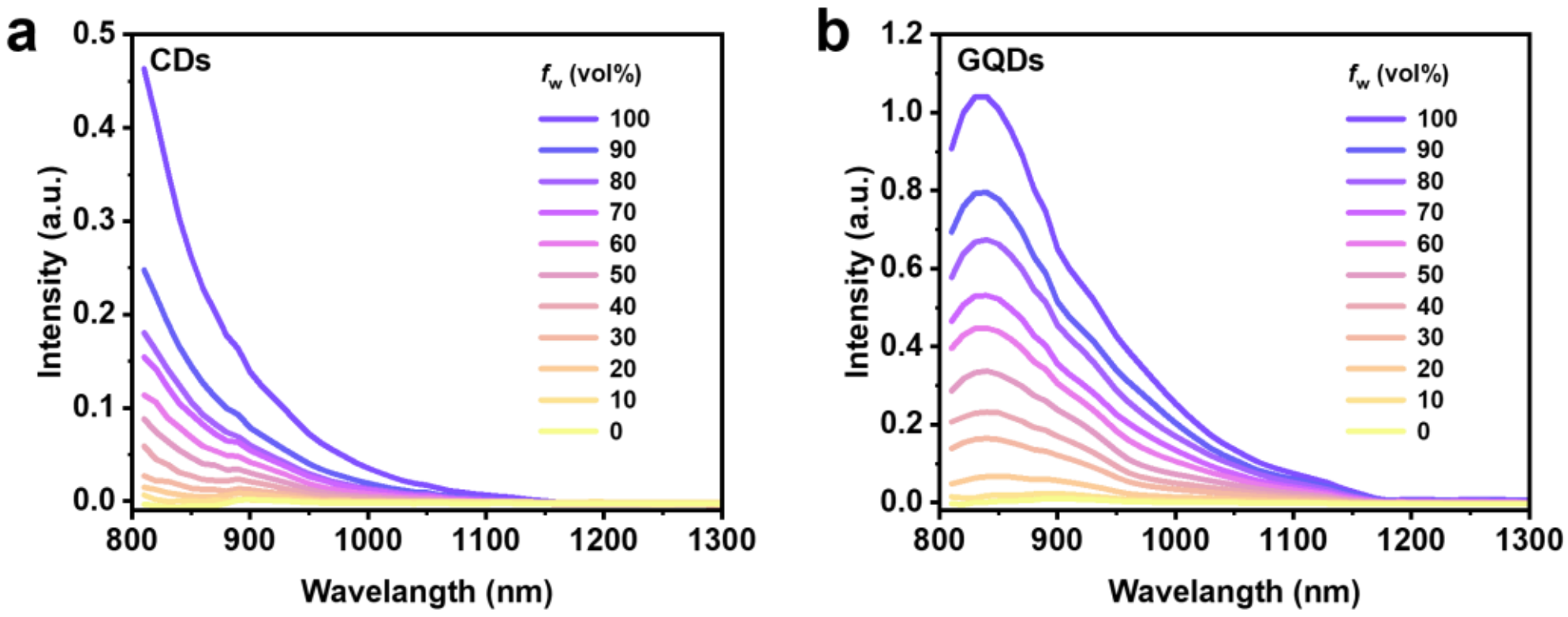

Figure S55. Fluorescence change analysis in varied polarity solvents. The near-infrared fluorescence spectra of (a) CDs and (b) GQDs under 808 nm excitation in THF/water mixed solutions. CDs and GQDs exhibited pronounced quenching of near-infrared fluorescence with decreasing the content of aprotic THF solvent.

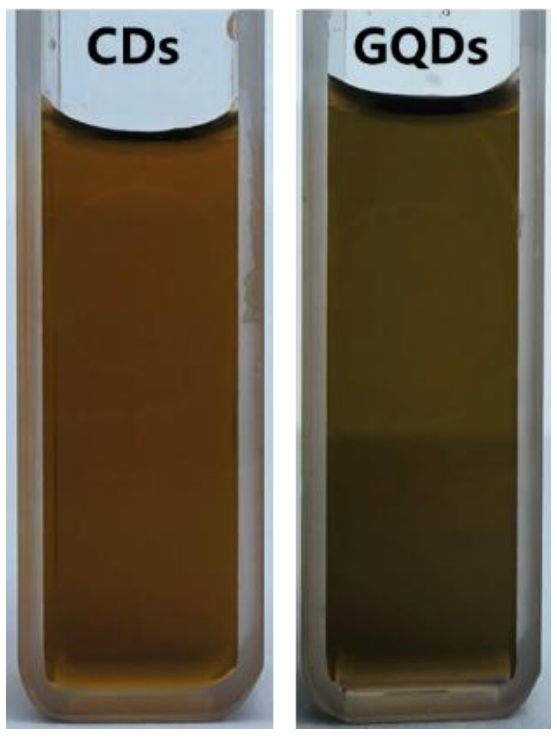

Figure S56. The digital image of CDs, GQDs in DMSO.

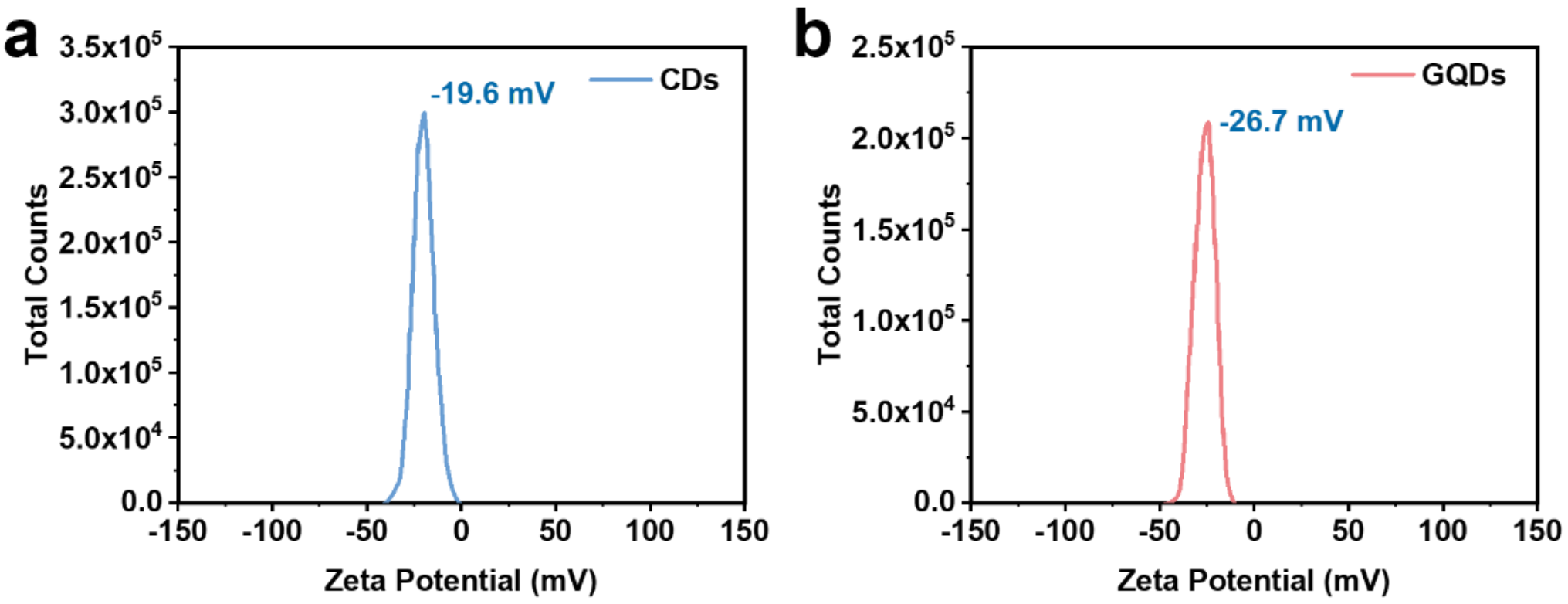

Figure S57. Surface charge analysis of CDs and GQDs. Zeta potential measurements of (a) CDs and (b) GQDs in water.

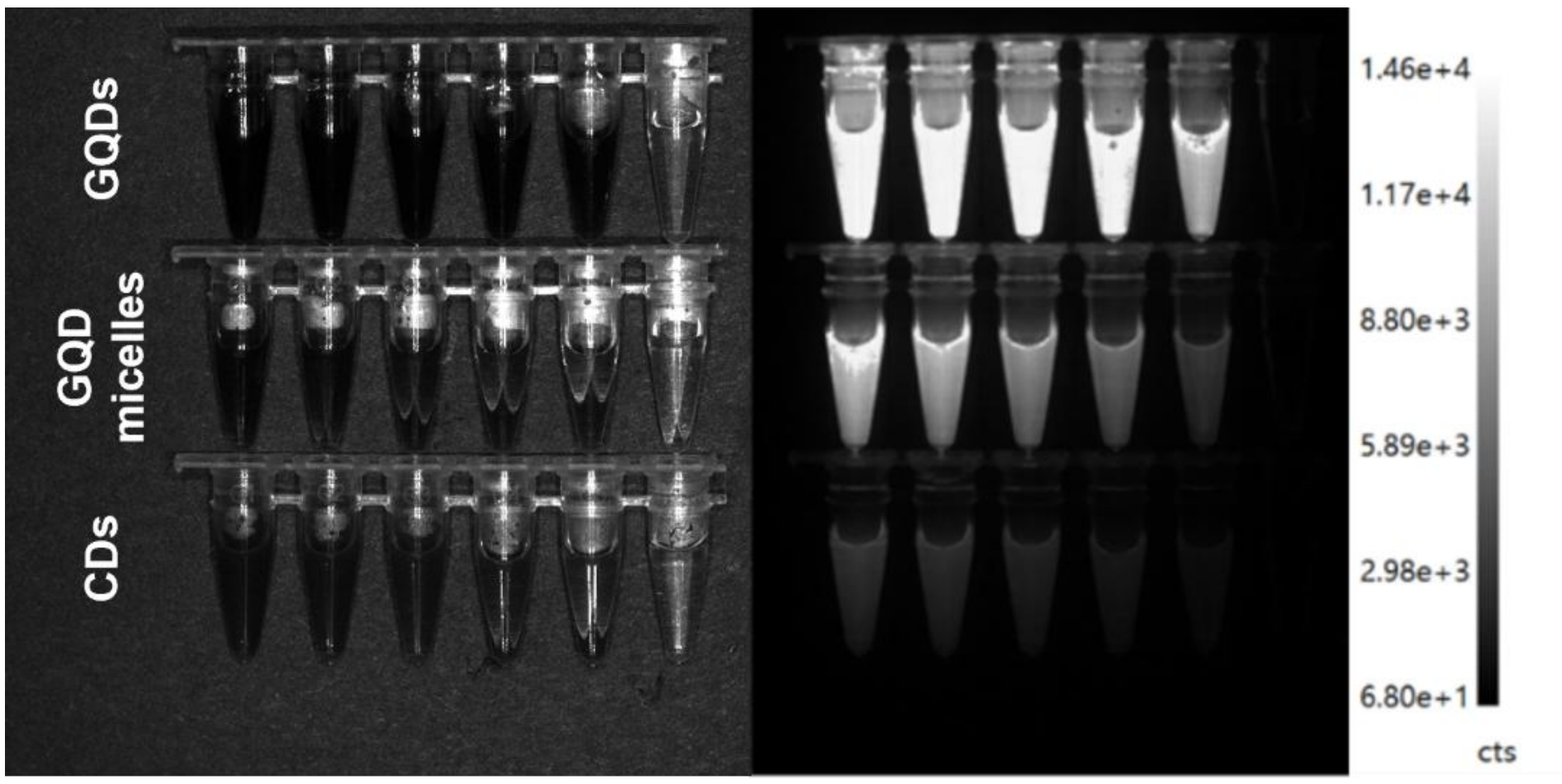

Figure S58. Comparative analysis of NIR fluorescence signals. Bright-field and near-infrared fluorescence images of CDs, GQDs, and GQD micelles solutions at different concentrations. (CDs and GQDs were dissolved in DMSO; GQD micelles were dispersed in water. Imaging conditions: 808 nm laser excitation, 1100 nm long-pass filter, exposure time 0.01 s).

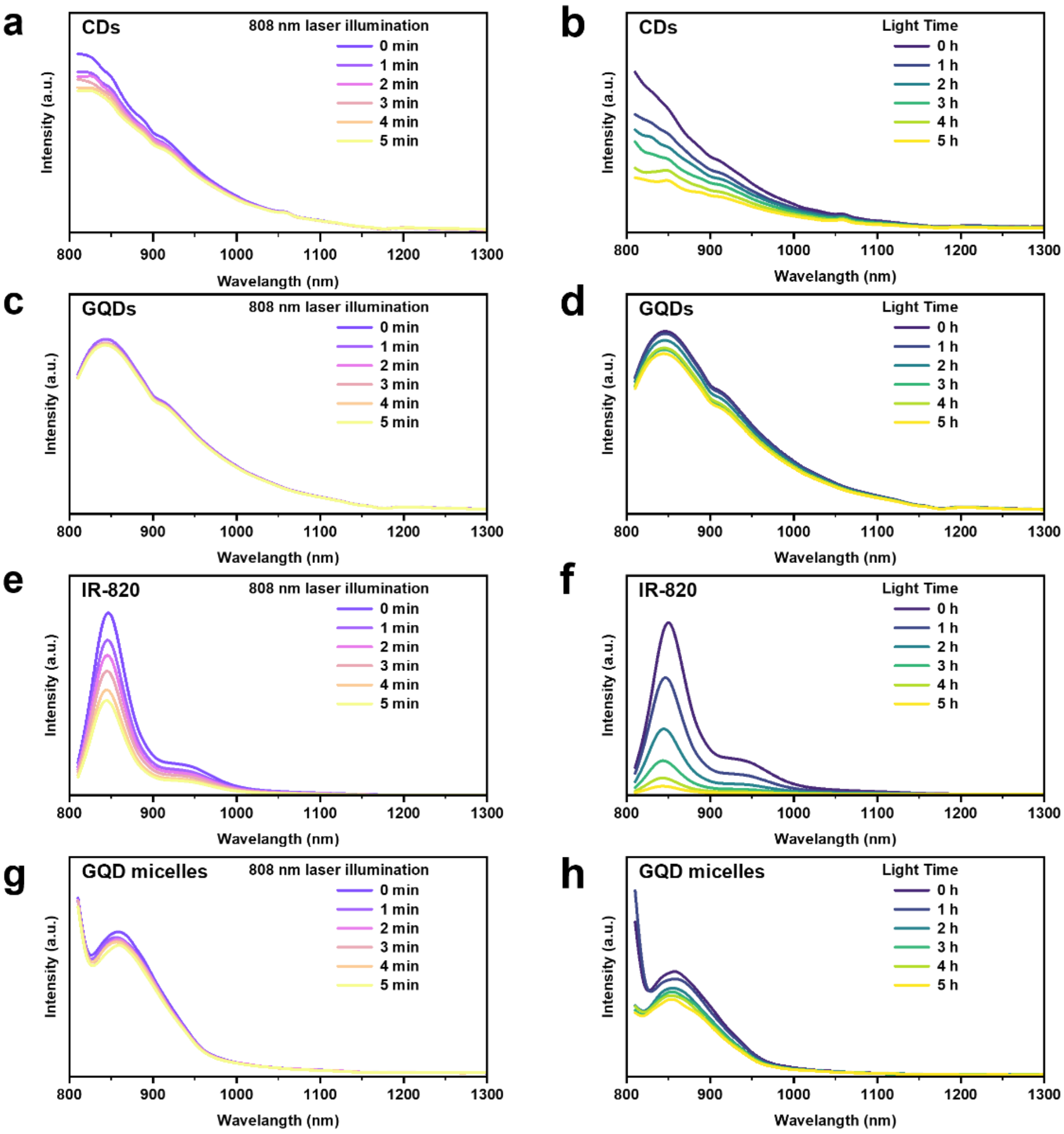

Figure S59. Photostability Assessment of NIR Fluorescence. The near-infrared fluorescence spectra of (a), (b) CDs, (c), (d) GQDs, (e), (f) IR-820, and (g), (h) GQD micelles under irradiation at different time intervals with 100 W m^{-2} xenon lamp and 1 W cm^{-2} 808 nm laser.

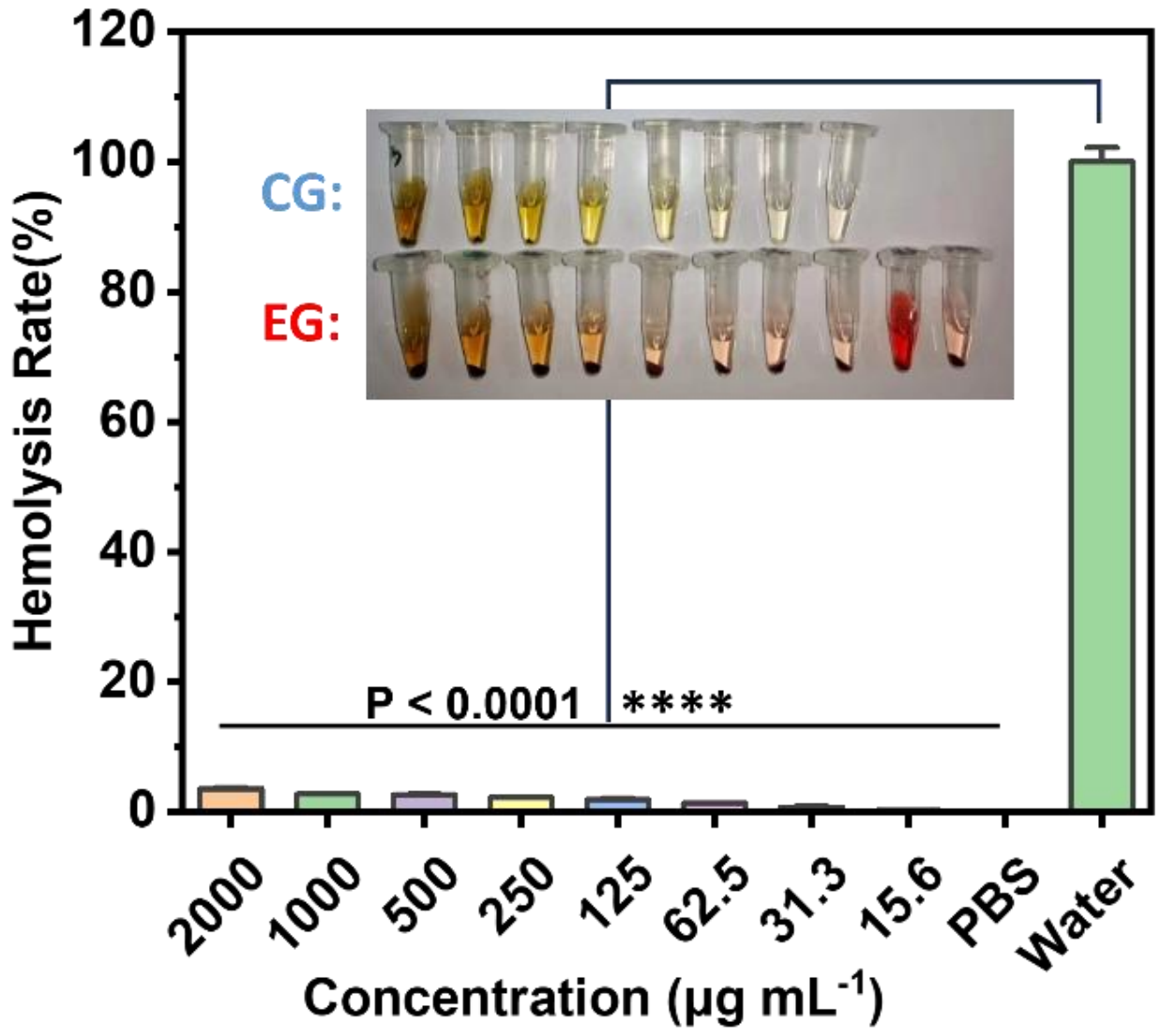

Figure S60. Hemolysis assay of GQD micelles. Hemolysis of red blood cells (RBCs) after co-incubation with varying concentrations of GQD micelles. Control Group (CG): GQD micelles; Experimental Group (EG): GQD micelles + RBCs. The coloration observed in the supernatant of high-concentration solutions originates from GQD micelles that were not sedimented during centrifugation.

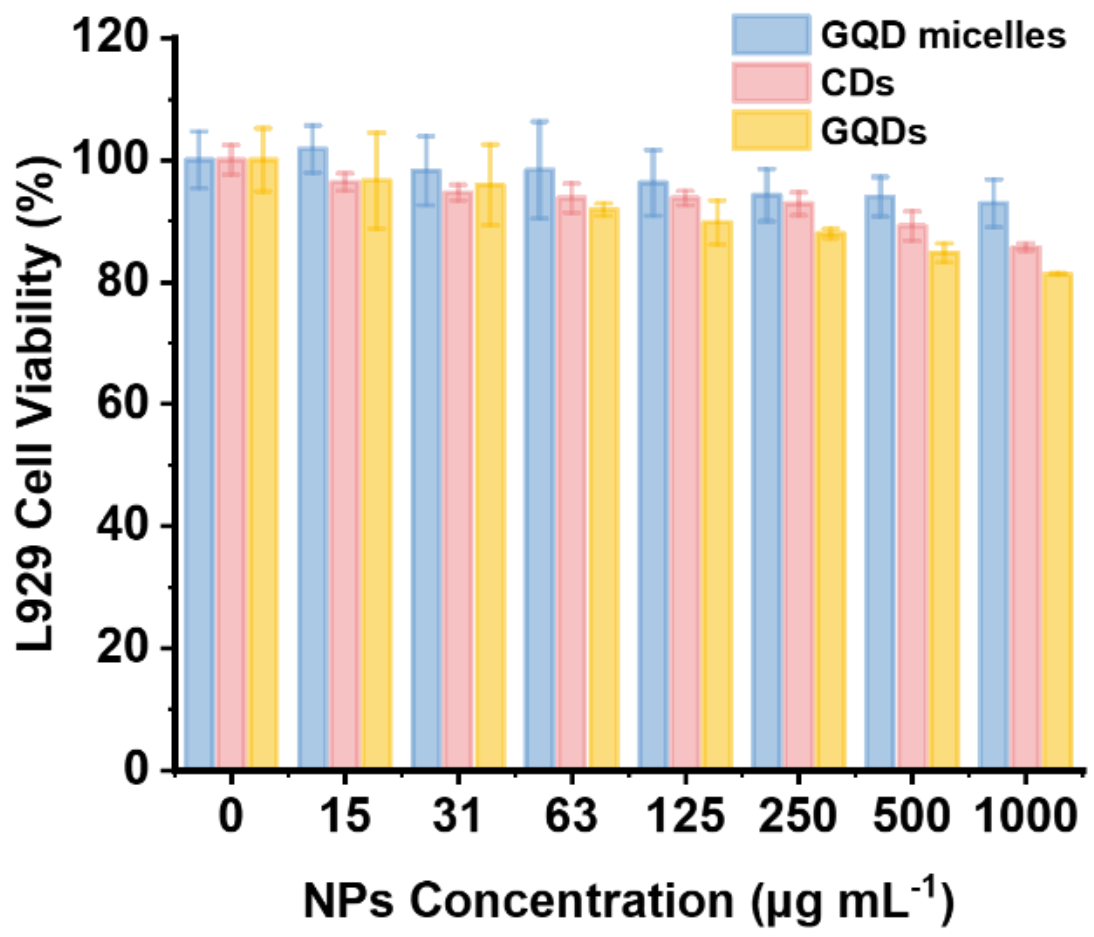

Figure S61. Cytotoxicity of GQD micelles. Cell viability of L929 cells treated with CDs, GQDs and GQD micelles for 24h (n = 4). Data are presented as mean ± standard deviation. The results showed that GQD micelles were biocompatible even the concentration up to 1.0 mg mL^{-1}.

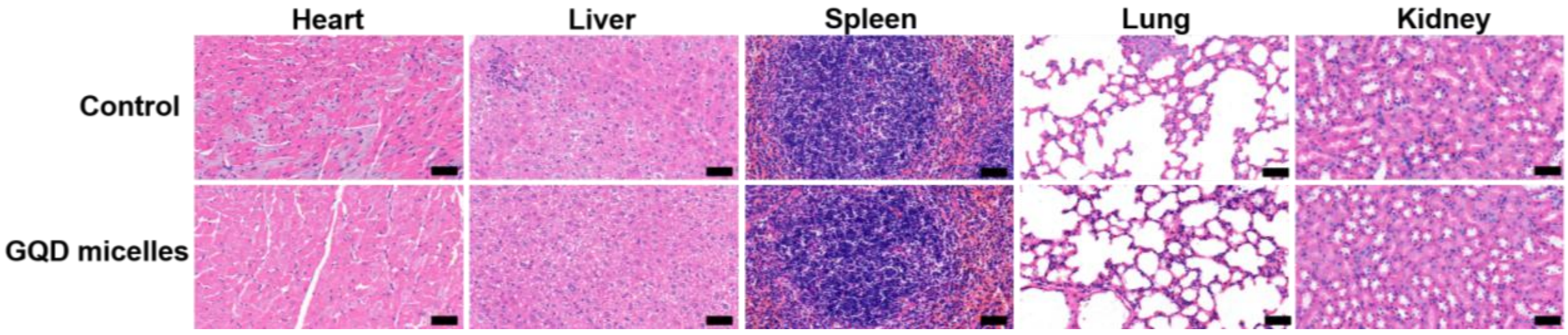

Figure S62. Biosafety evaluation of GQD micelles in vital organs. Hematoxylin and eosin (H&E) staining of main organs in mice after GQD micelles (1 mg mL^{-1}, 100 μL) injection for 24 h. Scale bar: 20 μm.

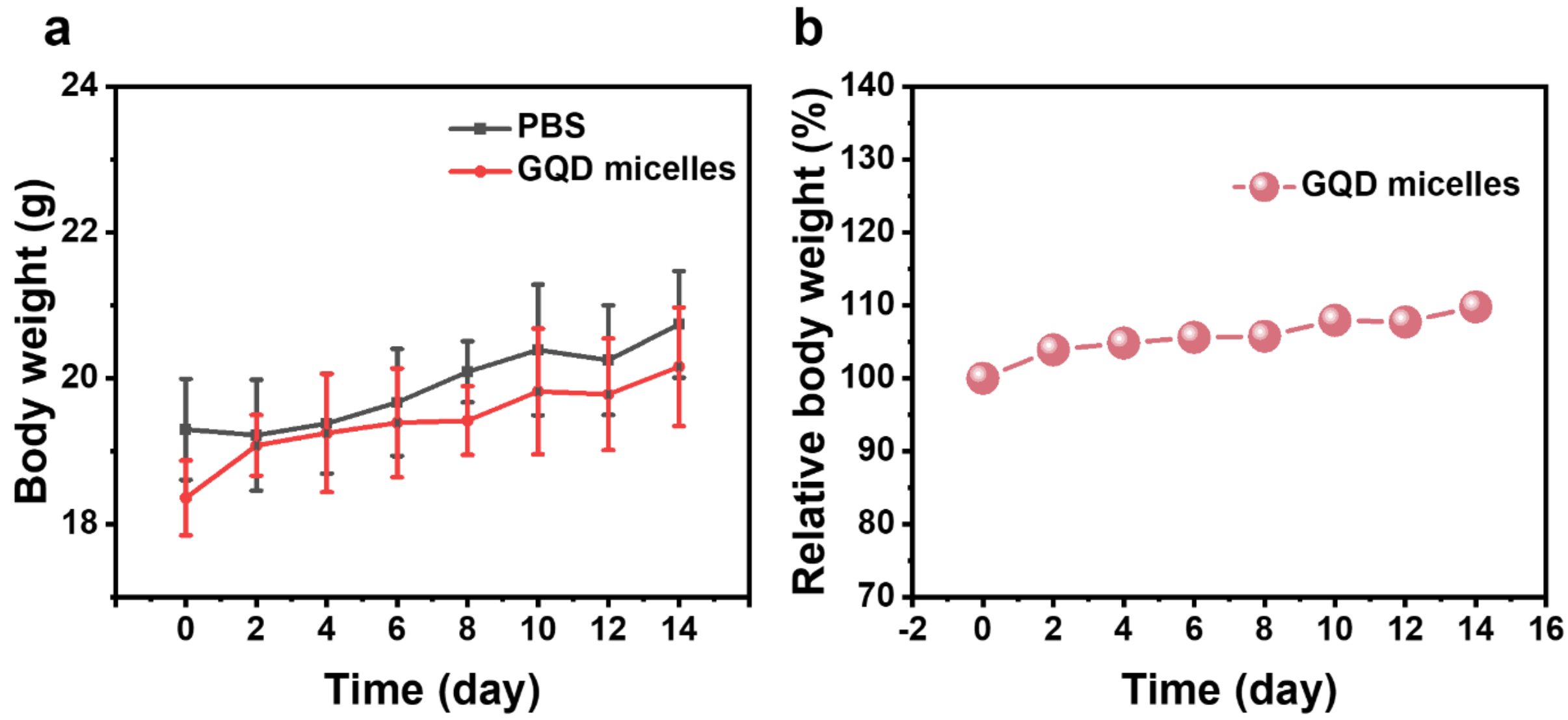

Figure S63. (a) Body weight changes in mice treated with PBS or GQD micelles (1 mg/mL) over 14 days. (b) Relative body weight of GQD micelles-treated mice (Day 0 = 100%).

10. In vivo fluorescence characterization

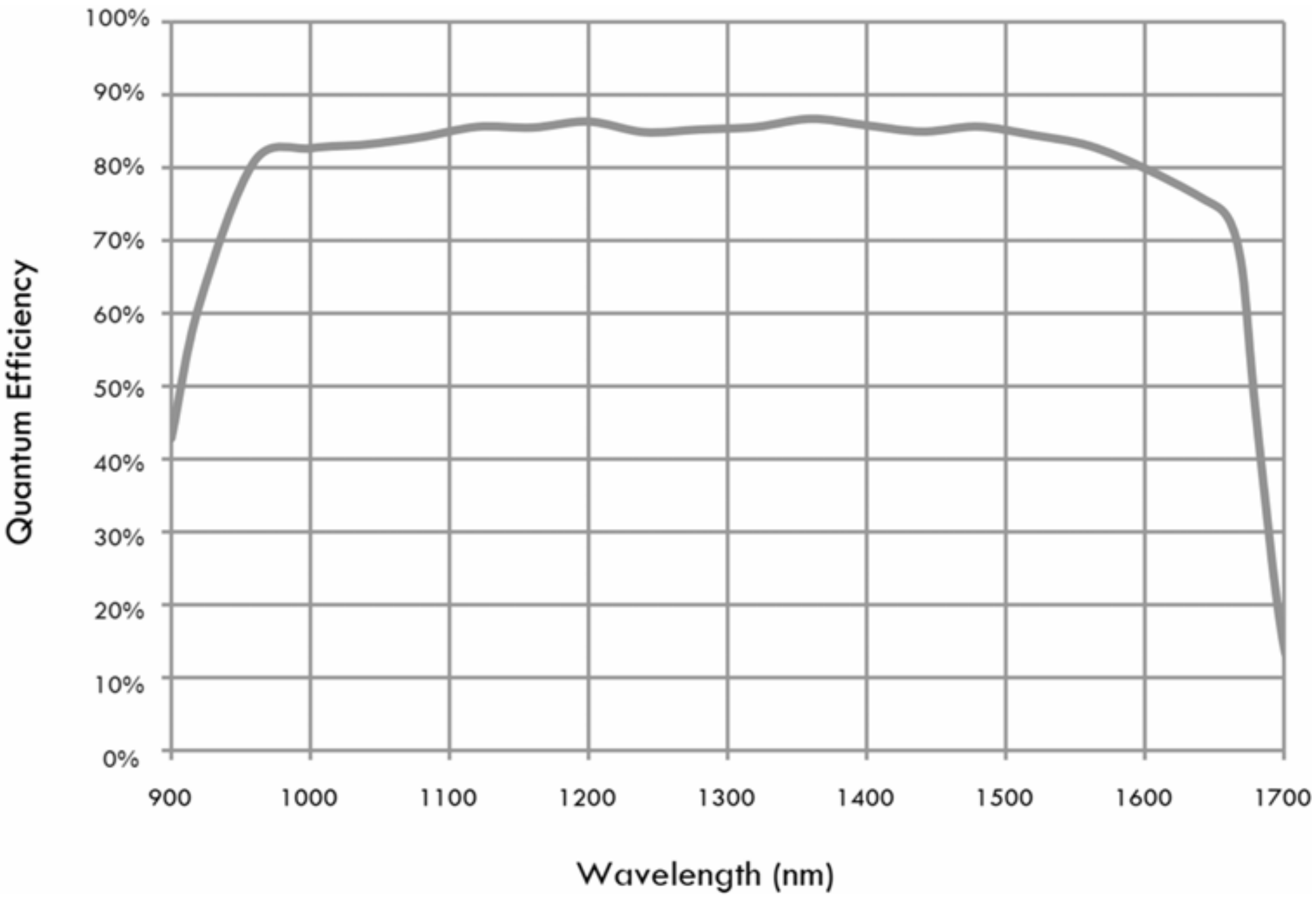

Figure. S64. Quantum efficiency of the NIRvana camera for imaging. The results showed the NIRvana HS exhibits a quantum efficiency of over 80% across an extraordinarily broad 950-1600 nm spectral band, thus enabling high-fidelity low-light imaging throughout the entire NIR-II/SWIR optical window.

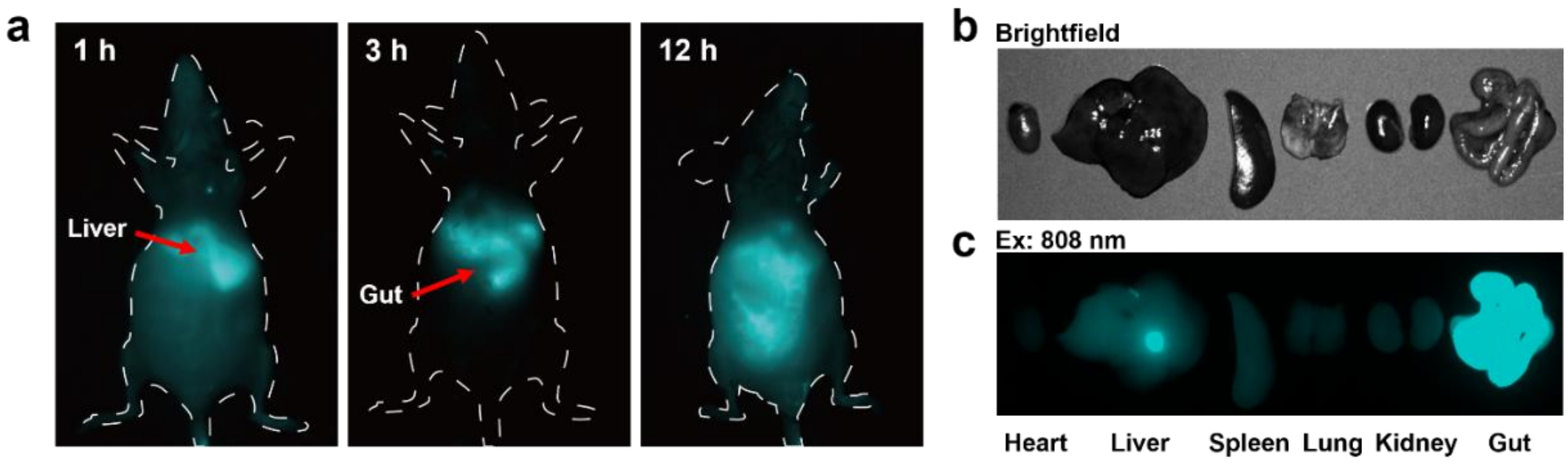

Figure S65. *In vivo* metabolism study. (a) The *in vivo* metabolic process of GQD micelles in mice and (b), (c) *ex vivo* imaging of tissues (1 mg mL^{-1}, 100 μL). After tail vein injection, GQD micelles gradually accumulated in the liver within 1 h and entered intestinal metabolism at 3 h. *Ex vivo* imaging showed that most of GQD micelles had accumulated in the intestinal region at 12 h post-injection (excitation: 808 nm, long-pass filter: 1100 nm).

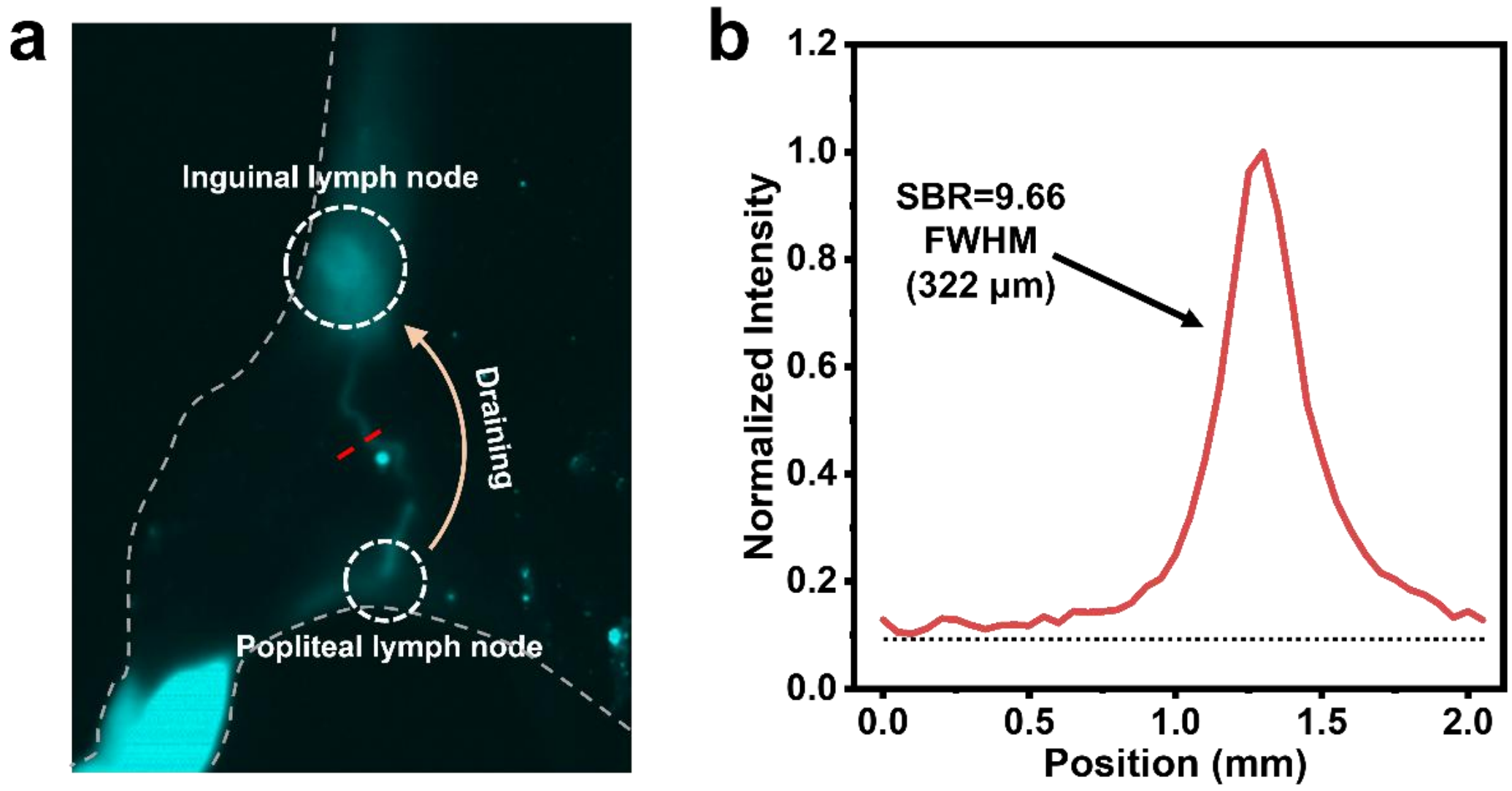

Figure S66. Lymphatic drainage imaging. (a) The Near-infrared fluorescence imaging of lymphatic drainage. (b) Fluorescence intensity profile along the dashed line in (a). Following injection into the footpad with gentle massage, the GQD micelles facilitate a marked drainage pathway from the popliteal to the inguinal lymph node, which is clearly visible under NIR fluorescence (excitation: 808 nm, long-pass filter: 1300 nm).

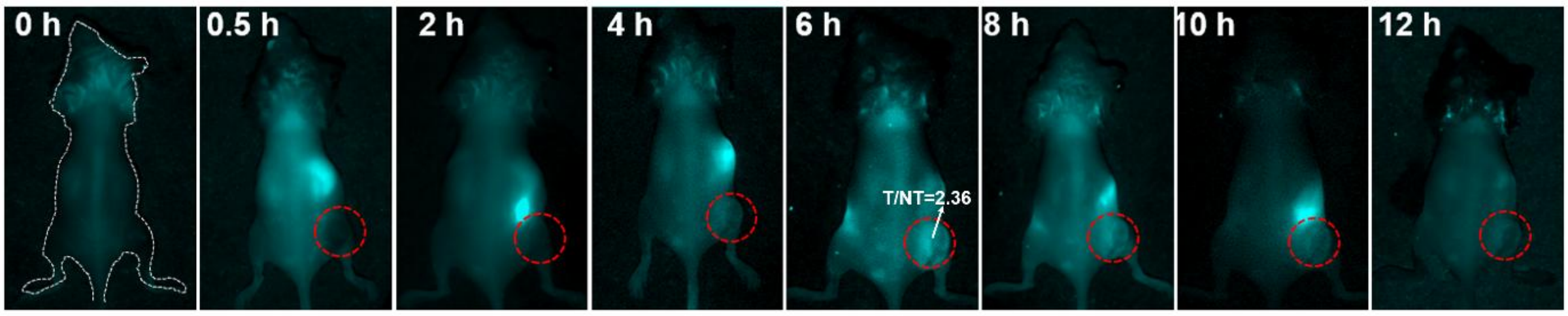

Figure S67. Fluorescence imaging of tumor accumulation. *In vivo* NIR-II fluorescence images of 4T1 tumor-bearing mouse after intravenous injection of GQD micelles in PBS (1 mg mL^{-1}, 100 μL) at time points of 0, 0.5, 2, 4, 6, 8, 10, and 12 h (excitation: 808 nm, long-pass filter: 1100 nm).

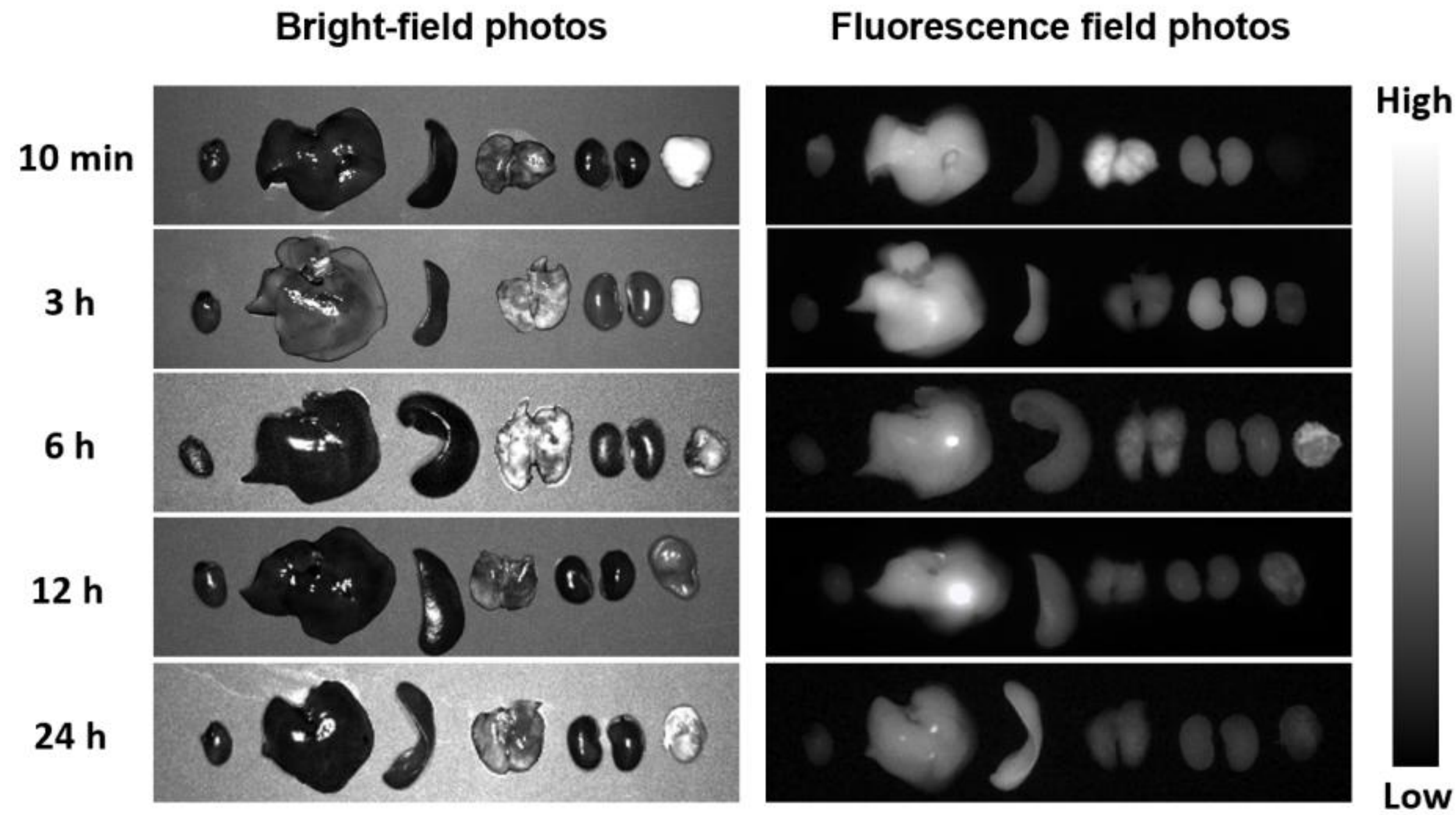

Figure S68. *In vivo* metabolism study of GQD micelles. Bright-field and fluorescence field photos of major mice organs at several time points before and after the intravenous injections of 100 μL GQD micelles solution (in PBS) at 1 mg mL^{-1} (excitation: 808 nm, long-pass filter: 1100 nm).

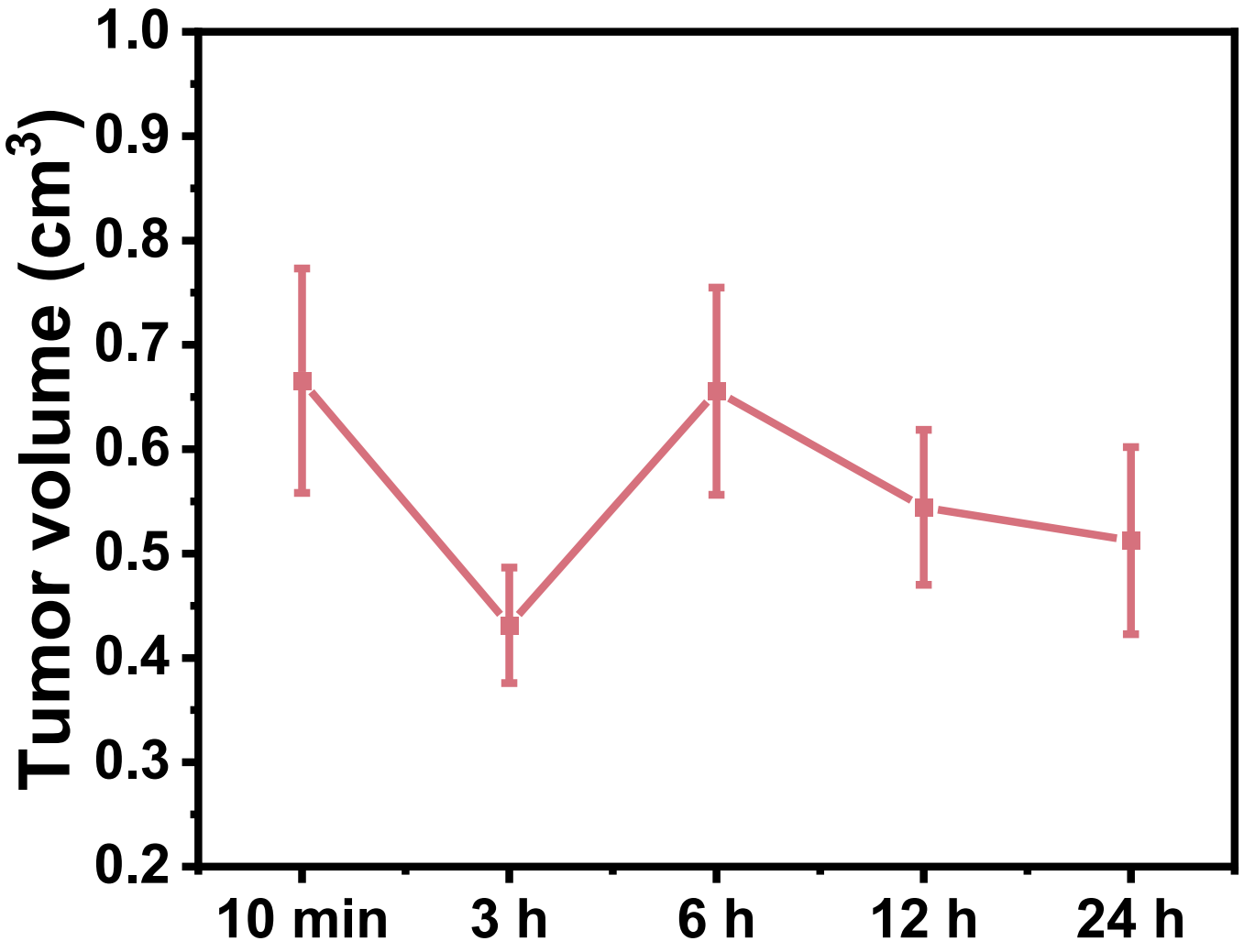

Figure S69. Time-dependent tumor volume after GQD micelles injection. Data are mean ± SD.

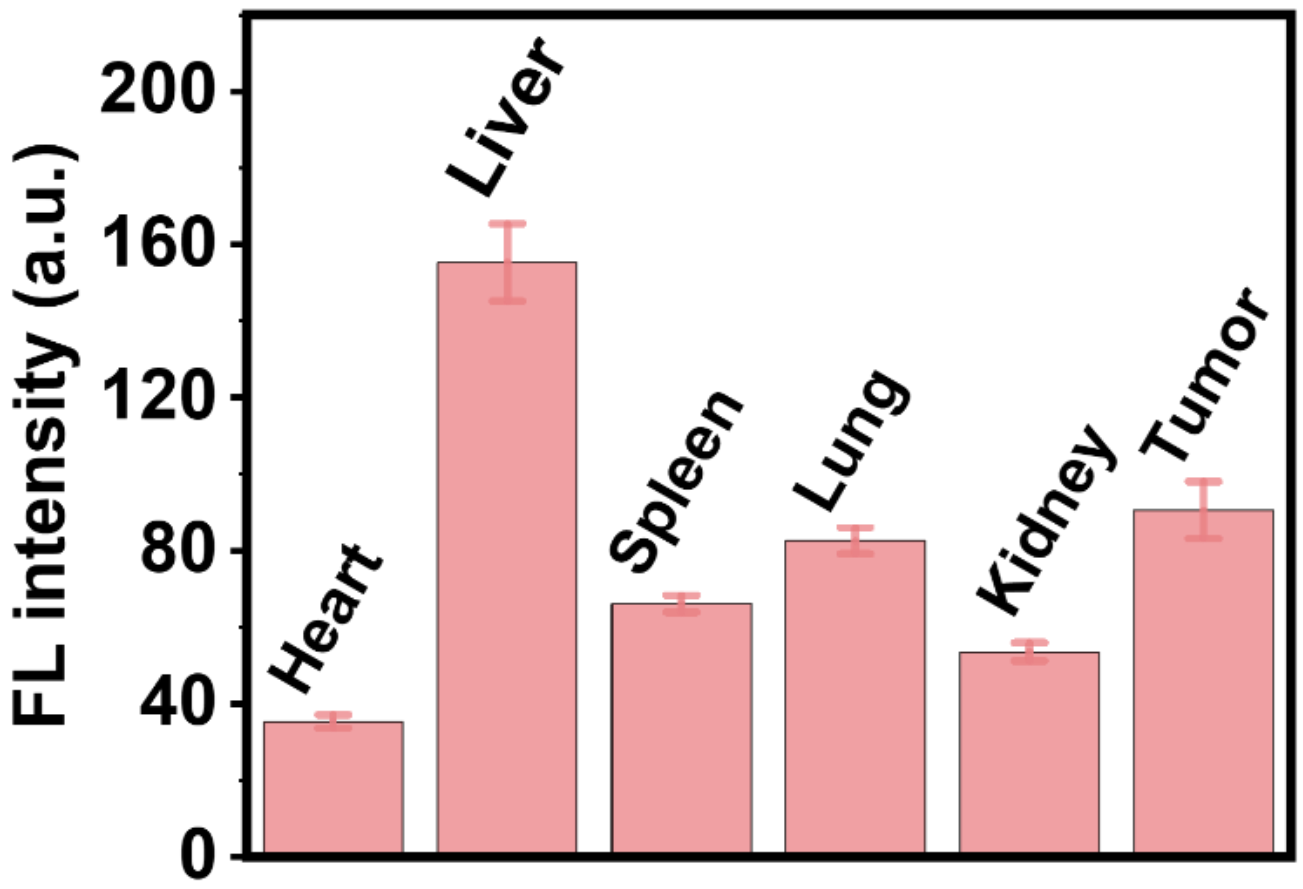

Figure S70. Quantification of fluorescence signals after 6 hours. Corresponding fluorescence intensities of different organs and tumors excised from 4T1 tumor-bearing mice at 6 h post-injection of GQD micelles.

Table S1. XPS fitting data of sample.

	C element	N element	O element	Cl element
CDs	72.72%	4.91%	14.59%	6.46%
GQDs	72.48%	7.52%	15.77%	4.23%
Rate of change	decrease of 0.23%	increase of 2.61%	increase of 1.18%	decrease of 2.23%

Table S2. Optical parameters of GQDs.

Excitation light	λ_{em} (nm)	ε ($cm^{-1}mol\ L^{-1}$)	PLQY (%)	Brightness (ε x Φ)
808 nm	855	$3.95x10^5$	1.8%	$7.1x10^3$

Table S3. Quantitative analysis of radical in GQDs.

	Weight (mg)	Molar mass (g mol^{-1})	EPR peak area	Purity(q)	Purity(s)
TEMPO	0.5	156.25	706.8	0.996	/
GQDs	5	1822.99	62188.5	0.754	0.647

The terms Purity(q) and Purity(s) denote the radical purity of GQDs calculated using qEPR and SpinCount software methodologies, respectively. The observed discrepancies between Purity(q) and Purity(s) likely originate from positional variations of the sample within the EPR resonator cavity during measurements and batch-to-batch heterogeneity in the GQD material.

Table S4. photoluminescence quantum yields in visible and NIR regions.

	Ex: 450 nm	Ex: 660 nm	Ex: 808 nm
CDs	21.0%	5.10%	0.30%
GQDs	0.09%	0.23%	1.77%

Table S5. Optical parameters of CDs and GQDs.

	PLQY	t_{ave}/ns	k_r/s	k_{nr}/s
CDs	21%	5.05	4.16×10^7	1.56×10^8
GQDs	0.09%	2.42	3.72×10^5	4.13×10^8

The rate constants for radiative decay (k_r) and non-radiative decay (k_{nr}) were calculated by the following equation:

$$k_r = \frac{\text{PLQY}}{\tau_{\text{ave}}} \qquad \text{Eq. (S16)}$$

$$k_{nr} = \frac{1 - \text{PLQY}}{\tau_{\text{ave}}} \qquad \text{Eq. (S17)}$$

Table S6. Double-exponential decay time constants of TCSPC.

sample	solution	λ_{em}	τ_1 (ns)	B_1	τ_2 (ns)	B_2	τ_{ave} (ns)
CDs	DMSO	540 nm	1.74 (7.06%)	0.19	5.31 (92.94%)	0.82	5.054
		600 nm	1.07 (5.57%)	0.22	4.99 (94.43%)	0.80	4.772
		700 nm	2.00 (6.00%)	0.15	5.53 (94.00%)	0.85	5.312
		800 nm	1.79 (11.58%)	0.29	5.43 (88.42%)	0.73	5.003
		850 nm	1.4 (9.92%)	0.30	5.30 (90.08%)	0.72	4.906
	DMSO + methanol	540 nm	1.69 (8.90%)	0.23	5.10 (91.10%)	0.78	4.796
		600 nm	1.54 (8.27%)	0.23	5.04 (91.73%)	0.78	4.753
		700 nm	2.07 (7.80%)	0.18	5.24 (92.20%)	0.84	4.995
		800 nm	1.50 (7.45%)	0.22	5.06 (92.55%)	0.81	4.799
		850 nm	1.40 (6.94%)	0.21	4.93 (93.06%)	0.80	4.675
GQDs	DMSO	540 nm	0.92 (57.98%)	0.93	4.43 (42.02%)	0.14	2.423
		600 nm	0.92 (18.67%)	0.51	4.17 (81.33%)	0.49	3.561
		700 nm	1.42 (16.49%)	0.43	5.07 (83.51%)	0.61	4.470
		800 nm	0.81 (32.04%)	0.77	4.41 (67.94%)	0.30	3.253
		850 nm	0.73 (44.18%)	0.89	4.32 (55.82%)	0.19	2.756
	DMSO + methanol	540 nm	1.46 (5.70%)	0.15	4.21 (94.30%)	0.86	4.052
		600 nm	1.01 (4.36%)	0.18	4.86 (95.64%)	0.82	4.689
		700 nm	0.83 (4.36%)	0.41	4.60 (89.34%)	0.62	4.202
		800 nm	0.71 (47.54%)	0.96	4.70 (52.46%)	0.16	2.792
		850 nm	0.65 (54.27%)	1.00	4.98 (45.73%)	0.11	2.611

The fluorescence lifetime spectra of the material were measured in different solvents. λ_{em} denotes the wavelength of the collected photoluminescence. τ_1 and τ_2 represent the fluorescence lifetimes of distinct decay components, while B_1 and B_2 correspond to the pre-exponential factors for each decay component. The average fluorescence lifetime (τ_{ave}) was calculated from these parameters. All values were derived from a biexponential decay fitting based on Eq. (S2) and Eq. (S3).

11.References

1. Frisch, M.J. et al. (Wallingford, CT; 2016).
2. Liu, Z., Lu, T. & Chen, Q. An sp-hybridized all-carboatomic ring, cyclo 18 carbon: Electronic structure, electronic spectrum, and optical nonlinearity. Carbon 165, 461-467 (2020).
3. Humphrey, W.F., Dalke, A. & Schulten, K.J.J.o.m.g. VMD: visual molecular dynamics. J. Mol. Graphics 14, 33-38 (1996).

4 Matsumoto, N., Itoh, N. & Yamasaki, K. Absolute Quantification of Pure Free Radical Reagents by Combination of the Effective Magnetic Moment Method and Quantitative Electron Paramagnetic Resonance Method. *Anal. Chem.* **94**, 12595–12603 (2022).

5 Liu, Z., Lu, T. & Chen, Q. An sp-hybridized all-carboatomic ring, cyclo 18 carbon: Electronic structure, electronic spectrum, and optical nonlinearity. *Carbon* **165**, 461-467 (2020).

6 Guo, H. *et al.* High stability and luminescence efficiency in donor-acceptor neutral radicals not following the Aufbau principle. *Nat. Mater.* **18**, 977-984 (2019).

7 Ghosh, P. *et al.* Decoupling excitons from high-frequency vibrations in organic molecules. *Nature* **629**, 355-362 (2024).

8 Zhou, Q., Guo, Y. & Zhu, Y. Photocatalytic sacrificial H2 evolution dominated by micropore-confined exciton transfer in hydrogen-bonded organic frameworks. *Nat. Catal.* **6**, 574-584 (2023).
